# Global research trends on the human exposome: a bibliometric analysis (2005–2024)

**DOI:** 10.1007/s11356-025-36197-7

**Published:** 2025-03-08

**Authors:** Pascal Petit, Nicolas Vuillerme

**Affiliations:** 1 AGEIS, Université Grenoble Alpes, 38000 Grenoble, France; 2 Institut Universitaire de France, Paris, France; 3 Laboratoire AGEIS, Université Grenoble Alpes, Bureau 315, Bâtiment Jean Roget, UFR de Médecine, Domaine de La Merci, 38706 La Tronche Cedex, France

**Keywords:** Exposome, Digital environmental health, Digital public health, Disease, Scientometrics, Bibliometrics

## Abstract

Exposome represents one of the most pressing issues in the environmental science research field. However, a comprehensive summary of worldwide human exposome research is lacking. We aimed to explore the bibliometric characteristics of scientific publications on the human exposome. A bibliometric analysis of human exposome publications from 2005 to December 2024 was conducted using the Web of Science in accordance with PRISMA guidelines. Trends/hotspots were investigated with keyword frequency, co-occurrence, and thematic map. Sex disparities in terms of publications and citations were examined. From 2005 to 2024, 931 publications were published in 363 journals and written by 4529 authors from 72 countries. The number of publications tripled during the last 5 years. Publications written by females (51% as first authors and 34% as last authors) were cited fewer times (13,674) than publications written by males (22,361). Human exposome studies mainly focused on air pollution, metabolomics, chemicals (e.g., per- and polyfluoroalkyl substances (PFAS), endocrine-disrupting chemicals, pesticides), early-life exposure, biomarkers, microbiome, omics, cancer, and reproductive disorders. Social and built environment factors, occupational exposure, multi-exposure, digital exposure (e.g., screen use), climate change, and late-life exposure received less attention. Our results uncovered high-impact countries, institutions, journals, references, authors, and key human exposome research trends/hotspots. The use of digital exposome technologies (e.g., sensors, and wearables) and data science (e.g., artificial intelligence) has blossomed to overcome challenges and could provide valuable knowledge toward precision prevention. Exposome risk scores represent a promising research avenue.

**Supplementary Information:**

The online version contains supplementary material available at 10.1007/s11356-025-36197-7.

## Introduction

**Fig. 1:**
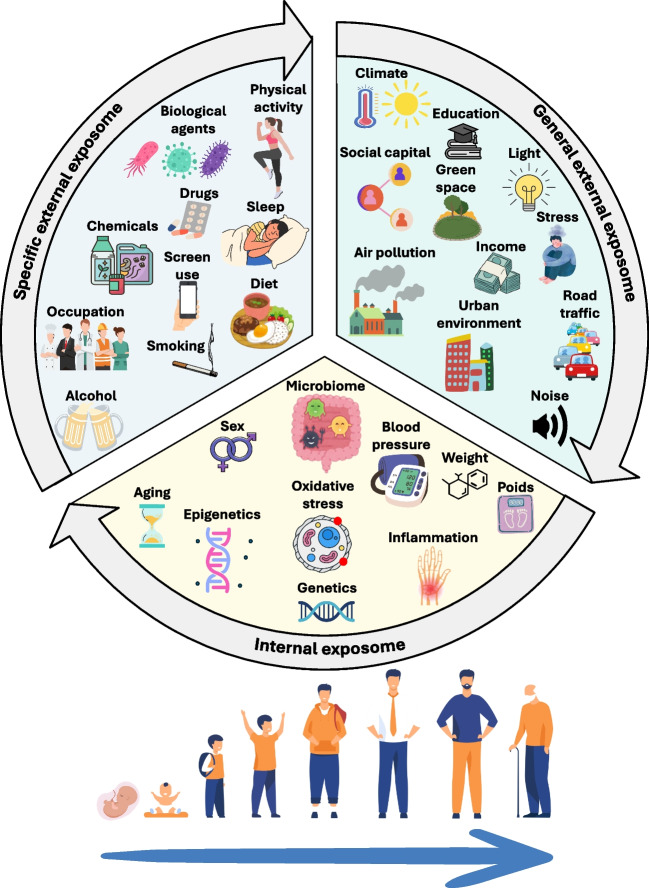
The exposome concept and its domains

Understanding and identifying the factors that contribute to the onset and development of diseases is crucial for public health in order to determine relevant and effective public health interventions that can promote health and well-being for all. Genetics is believed to account for about 10% of diseases, while environmental factors and gene-environment interactions appear to be the remaining causes (Zhang et al. 2021). Hence, environmental exposures throughout the life course play an important role in the onset and development of diseases. The exposome term/concept, first formulated in 2005 by Dr. Wild (Wild 2005), is a complement of the genome that can be defined as the holistic study of all the lifetime environmental exposures (physical, chemical, biological, psychosocial, social, behavioral, etc.) from conception to death (Barouki et al. 2022; Buck Louis et al. 2017; Dennis et al. 2017; Guillien et al. 2023; Juarez et al. 2014; Kim and Hong 2017; Maitre et al. 2022a; Miller and Jones 2014; Niedzwiecki et al. 2019; Price et al. 2022; Robinson and Vrijheid 2015; Siroux et al. 2016; Vermeulen et al. 2020; Wild 2005, 2012). In 2012, the exposome definition was refined with the classification of the exposome into three overlapping domains that can change over time: the internal exposome (e.g., aging, oxidative stress, metabolism, gut microbiome), the general external exposome (e.g., climate, built environment), and the specific external exposome (e.g., chemical exposure, lifestyle, occupations) (Haddad et al. 2019; Santos et al. 2020; Wild 2012) (Fig. 1).

Exposome is a relatively new, emerging, evolving field and mainstream concept that can be considered one of the hallmarks of environmental and public health research for the last decade (Abdelzaher et al. 2022; Barouki et al. 2022; Siroux et al. 2016). A deep understanding of the exposome is crucial for enabling both population-level and personalized (or precision) prevention efforts (Barouki and Lévi 2024; Bíró et al. 2018). The exposome has inspired numerous researchers worldwide and is expected to influence environmental and public health research, practices, and policies (Barouki et al. 2022). Numerous studies presenting, challenging, and favoring the exposome concept and utility have been published (Haddad et al. 2019). In addition, the number of publications and citations for “exposome” has increased over recent years (Haddad et al. 2019; Kiossoglou et al. 2017; Zhang et al. 2021); from six citations in PubMed in 2005 to 150 citations in 2010 and > 1600 citations in 2015 (Siroux et al. 2016).

Despite its increasing popularity and scientific importance, to our knowledge, no study has attempted to comprehensively summarize worldwide research trends/hotspots of the entire human exposome research field, with the exception of one study mainly focusing on the impact of the exposome concept in the field of dermatology (Molina-García et al. 2022). It is paramount to improve our understanding of the human exposome research field in order to advance the utility and societal impact of the exposome. To contribute to this effort, we conducted a bibliometric analysis that aims to present the state of knowledge and emerging trends of exposome from 2005 to December 2024 by providing an overview of the interdisciplinarity and connectedness (structural relationships between different research constituents) of the entire human exposome research field. Bibliometric analysis is a recognized and rigorous technique that uses a set of quantitative methods to measure, track, and analyze scholarly literature (Cobo et al. 2011; Donthu et al. 2021). Our secondary objectives were to (i) examine potential sex differences regarding scholarly impact and academic influence; (ii) investigate the impact of gross domestic product (GDP), population size, and the number of researchers in research and development (R&D) on scientific productivity; (iii) summarize and get a complete picture of health outcomes and potential risk factors investigated within the human exposome research field; and (iv) investigate trends regarding digital technologies involved in human exposome research.

## Materials and methods

A bibliometric approach was conducted to analyze the research trends on the human exposome by time and number of publications, countries of publications, most active journals, institutions, authors, funding bodies, subject areas, citations of publications, and keywords of publications. This work was conducted from January 2023 to July 2023 and updated in January 2025.

### Search strategy and selection criteria

To develop and validate the search strategy, a literature search was conducted to identify reviews on the human exposome. From this search, we identified one recent scoping review on human exposome studies (Haddad et al. 2019). In line with this scoping review, the following retrieval strategy using topic terms was carried out for identifying records on the human exposome: TS = (exposom*). A pilot run was conducted to validate the search strategy using the Web of Science Core Collection (WoSCC) database as a bibliometric data source. The validity of our search results was confirmed by making reference to the publications identified in the aforementioned scoping review (Haddad et al. 2019). The final literature search was conducted using WoSCC, according to the Preferred Reporting Items for Systematic Reviews and Meta-Analyses (PRISMA) guidelines (Tricco et al. 2018), following a single screening approach (i.e., one author performing the literature search and bibliometric analysis). Only the WoSCC database was used because for other bibliographic databases (e.g., PubMed), some metadata of interest for bibliometric analysis cannot be extracted (Aria and Cuccurullo 2017; Moral-Muñoz et al. 2020). To reduce the bias induced by daily database changes, all data collection (literature retrieval and data download) was conducted and completed on the same day, January 16, 2023. The search was repeated on January 14, 2025, to include publications from 2023 to 2024. The search was restricted to original peer-reviewed journal publications (i.e., articles, reviews, and peer-reviewed conference papers) published from January 1, 2005, (the year of the first exposome publication by Dr. Wild) (Wild 2005) to December 31, 2024. The only exception was the publication from Wild (2005), an editorial material. In order to guarantee the representativeness of the included publications, the search results were filtered by title and abstract to exclude irrelevant publications. Publications were included if they pertained to the human exposome (Table A.1 in the supplemental materials). Publications conducted solely in animals or in vitro, or publications not relevant to the exposome concept, were excluded. The protocol of this study was not registered.

### Data collection and processing

Several metadata were extracted, including keywords, authors’ names, country, institution, journal, publication date, funding bodies, and total citations. All of this data was manually checked, cleaned, and harmonized to lessen potential mistakes that could lead to an incorrect representation of the research field. For each record, the names of the primary (or first) and senior (or last) authors were collected, and then the online Genderize database was used to determine the author’s sex, as previously done in other works (Chatterjee and Werner 2021). For each author’s first name, sex was assigned based on how frequently (probability) it occurs in public social media profiles where the sex of the user can be verified (Wais 2006). For unknown sex or for probability under 0.9, a manual internet-based search of the author’s sex was conducted. The population size, number of researchers in R&D, and GDP of each country were obtained from the World Bank.

### Data analysis

All analyses were performed using R software 4.4.1® (R Core Team, Vienna, Austria) for Windows 10©. The bibliometric analysis was performed using the bibliometrix package version 4.3.0 (Aria and Cuccurullo 2017; Moral-Muñoz et al. 2020), following published guidelines on bibliometric analyses (Donthu et al. 2021; Montazeri et al. 2023) (supplementary materials). Seven bibliometric metrics were computed (supplementary materials) (Bienert et al. 2015; Bihari et al. 2023; Kumar and Kumar 2008; Shao et al. 2022; Wu et al. 2022a). A two-sided Wilcoxon Mann–Whitney rank sum test was used to examine sex differences in authorship (primary and senior) regarding the number of publications and citations of peer-reviewed publications. Differences in publication and citation counts between different sex combinations of primary and senior author pairs were investigated using a one-way, two-sided Kruskal–Wallis test. The effect size was calculated using Cohen’s d. Single-author publications were excluded from analyses of senior authorship and dominance factor calculation. To determine whether sex differences in citation could be associated with an impact journal effect (Chatterjee and Werner 2021), several sensitivity analyses were conducted focusing exclusively on high-impact journals. Three definitions of high-impact journals were used: those with an impact factor ≥ 5, ≥ 10, and journals ranked in the top tier (Q1). Journals’ impact factors and ranks were retrieved manually using the Journal Citation Report in January 2025. The gender package version 0.6.0 and the genderizeR package version 2.1.1 were used to assign sex to the authors’ first names (Mullen 2021; Wais 2006). Spearman correlations were calculated to examine the association between the number of publications and GDP, population size (Wang and Zhao 2018), as well as the number of researchers in R&D (per million people). Research trends/hotspots were investigated with keyword frequency, co-occurrence (counting of paired keywords for keywords with a frequency > 10), and co-word analysis (study of the strength of association between keywords using a thematic map). To summarize and get a complete picture of health outcomes investigated within the human exposome research field, the co-occurrence between potential risk factor keywords (PRFKs) and specific health outcome categories (e.g., cancers, reproductive disorders) was studied. Research trends related to digital tools/techniques were also investigated by focusing on keywords related to digital technology (e.g., wearable sensors) and analytics (e.g., machine learning).

## Results

### Main characteristics

After the exclusion of 1321 irrelevant records and three duplicates, 931 publications were included in the analysis (Fig. A.1 and Table A.2, multimedia appendix). The main characteristics of the included publications are presented in Table A.3. Most publications were articles (70%), followed by reviews (28%), conference papers (1.8%), and editorial materials (0.11%) (Fig. A.2). Most of the publications (68%) were mostly open access. The publication’s average age was 4.41 years. Publications were cited 26,623 times, with an average of 28.6 citations per publication. There were 4529 authors, with 7815 author appearances and a mean of eight authors per publication. There were 45 (4.83%) single-authored publications that were written by 34 authors. On average, there were 61 references per publication.

### Scientific productivity

**Fig. 2:**
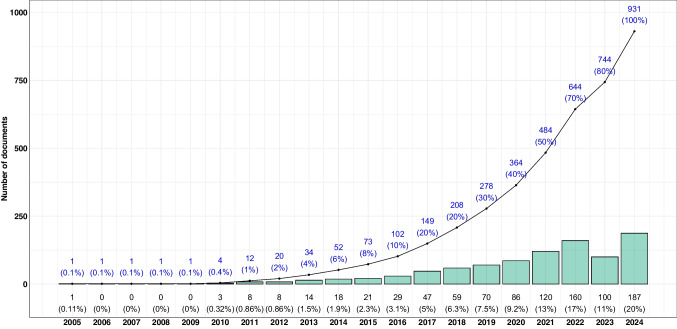
Scientific productivity—number of publications per year

There was a gap of 4 years between the publication from Wild (2005) and the second exposome publication accepted in 2009 and published online in 2010 (Fig. A.3) (Bonneterre et al. 2010). From 2010, there was a gradual and continuous increase in exposome publications, reaching a maximum of 187 (20.0%) in 2024 (Fig. 2), with an annual growth rate (AGR) that fluctuated over time, stabilizing around 20–30% in the last 5 years. In the last 10 years, the number of publications was multiplied by 20, while it tripled during the last 5 years. The average number of citations per year was 1331, with a maximum of 3060 citations in 2022.

### The most productive countries

Globally, 72 countries from all continents have contributed to human exposome research (Table A.4), with mostly multi-country publications. Europe was the most represented continent (*n* = 33 countries). Most publications originated from the United States (US) (*n* = 430, 46.2%), and four European countries (France, the United Kingdom (UK), Spain, and the Netherlands) (Fig. A.5–A.6). For these five countries, there was a gradual increase in the published research, reaching a maximum in 2024, with the exception of France reaching a maximum in 2022 (Fig. A.7–A.11). Czech Republic (86.7%), Canada (67.2%), and Spain (63.0%) had the highest mean AGR. There was a positive association between the number of publications and GDP (*ρ* = 0.68, *p* = 6.1e − 11), population size (*ρ* = 0.26, *p* = 0.03), and the number of researchers in R&D (*ρ* = 0.59, *p* = 1.4e − 7). Lithuania (6.7e − 10 publications/GDP), Suriname (5.5e − 10 publications/GDP), and Cyprus (4.2e − 10 publications/GDP) had the highest scientific production per capita, while Luxembourg (2.0e − 5 publications/inhabitants), Lithuania (1.7e − 5 publications/inhabitants), and Norway (1.2e − 5 publications/inhabitants) had the highest scientific production per population. The US (0.089), Nigeria (0.077), India (0.059), China (0.056), Spain (0.051), Columbia (0.045), Burkina Faso (0.042), and France (0.040) had the highest scientific production per researcher in R&D.

### The most cited publications

The most cited publications were open access, with the exception of reviews (Fig. A.12). The most cited publication was the paper from Wild (2005). The top 30 most-cited publications (Burbank et al. 2017; Celebi Sozener et al. 2022; Davis et al. 2023; Dennis et al. 2017; Gacesa et al. 2022; Go et al. 2015a,b; Janssen et al. 2015; Jones 2015; Jones et al. 2012; Kumar et al. 2020; Krutmann et al. 2017; Lochhead et al. 2015; Maitre et al. 2018; Miller and O’Callaghan 2015; Nikolich-Žugich 2018; Rappaport 2011, 2016; Rappaport et al. 2014; Ugai et al. 2022; Uppal et al. 2016; Vermeulen et al. 2020; Vineis et al. 2017; Vrijens et al. 2015; Vrijheid 2014; Vrijheid et al. 2014; Wild 2005, 2012; Wild et al. 2013; Wishart et al. 2015) were published in 26 different journals and had between 144 (0.54%) and 1457 (5.47%) citations (Table A.5). The most commonly studied topics among the top 30 most-cited publications included early-life exposures (*n* = 5), omics (*n* = 5), cancer (*n* = 4), allergies (*n* = 3), and metabolomics (*n* = 3).

### The most active journals

Publications were published in 363 journals, with 368 (39.5%) that were published in the top 20. Environment International, Environmental Research, Environmental Health Perspectives, and Environmental Science & Technology were the most active journals for publishing human exposome research, with 58 (6.23%), 35 (3.76%), 32 (3.51%), and 32 (3.44%) publications, respectively (Table A.6).

### The most prolific authors

The top 30 of the most prolific authors contributed to at least 16 (1.72%) publications (Table A.7). Half of them were females. Among the top 30, M. Vrijheid was the most productive in terms of total publications (70, 7.52%). D.P. Jones was the most productive in terms of fractionalized frequency (6.93), followed by M. Vrijheid (5.38) and P. Vineis (3.60). D. A. Sarigiannis was the most dominant in terms of first author among the top 30, with a dominance factor (DF) of 26.7%, followed by P. Vineis (21.4) and D.I. Walker (13.6). B. Warth was the most dominant in terms of last author among the top 30, with a DF of 72.2%, followed by D. P. Jones (62.5) and D. A. Sarigiannis (33.3). M. Vrijheid (30), R. Slama (24), and X. Basagana (24) had the highest h-indexes. M. Vrijheid (53), R. Slama (44), and L. Chatzi (41) had the highest g-indexes. J. Wright (2.75), L. Maitre (2.62), and M. Vafeiadi (2.50) had the highest m-indexes. R. Slama (70.7%), B. Heude (39.5%), and X. Basagana (37.4%) had the highest mean AGR among the top 30. Among the top 30 most prolific authors, there were an equal number of females (*n* = 15) and males (*n* = 15). The most frequently studied exposures by leading authors included chemicals, air pollution, smoking, pesticides, per- and polyfluoroalkyl substances (PFAS), endocrine-disrupting chemicals (EDCs), and phthalates (Fig. A.13). The most common health outcomes were reproductive disorders, neurodevelopmental disorders, and respiratory diseases (Fig. A.14).

### Sex differences in authorship

Among the 931 publications, 471 (50.5%) had females as primary authors (Table A.3). Of 897 multi-authored publications, 318 (35.4%) had females as senior authors. Overall, female primary and senior authors had fewer publications and were cited less compared to males (Table A.3). This pattern was not consistent across each year included in the study period (Table A.8 and Fig. A.15–A.16). Publications with female senior authors were cited the fewest times (6294 citations), whereas publications with males as senior authors (15631 citations) or both primary and senior authors (8554 citations) were cited the most. Publications with males as primary authors and females as senior authors had fewer citations (2404 citations) than publications with females as primary authors and males as senior authors (7077 citations) (Table A.9). Publications with females as both primary and senior authors (*n* = 201, 22.4%) were cited fewer times (3890 citations) than publications with males as both primary and senior authors (*n* = 309, 34.4%, 8554 citations). No evidence of a high-impact journal effect was found, as sex-based citation disparities persisted even when focusing on top-tier journals (Tables A.10–A.15 and Fig. A.17–A.22).

### The most active institutions

The top 20 of the most active institutions contributed to 35 (3.76%) or more publications during the study period (Table A.16). Among them, there were eight countries represented, with six institutions from the US, five from France, and three from Spain and the Netherlands. INSERM (France) was the institution with the highest number of publications (*n* = 132, 14.2%), followed by CIBER (Spain) (*n* = 98, 10.5%) and Pompeu Fabra University (Spain) (*n* = 95, 10.2%). INSERM (*n* = 5038, 18.9%), CIBER (*n* = 3096, 11.6%), and CNRS (France) (*n* = 2720, 10.2%) were cited the most. INSERM (37), CIBER (29), and CNRS (27) had the highest *h*-index. Icahn School of Medicine at Mount Sinai (US) (93%), Utrecht University (The Netherlands) (86%), and INSERM (72%) had the highest mean AGR among the top 20.

### The most active funding bodies

Funding sources were not reported for 177 (19.0%) publications. The top 20 of the most active funding bodies contributed to 22 (2.36%) or more publications (Table A.17). The National Institutes of Health (NIH) (*n* = 183, 19.7%), National Institute of Environmental Health Sciences (NIEHS) (*n* = 140, 15.0%), and European Commission (*n* = 101, 10.8%) funded the highest number of publications, had the highest number of citations, and had the highest citation indexes. The European Union (161%), French National Research Agency (ANR) (116%), and Norwegian Ministry of Health and Care Services (105%) had the highest mean AGR among the top 20.

### Trends in research topics

The research area with the highest number of publications was environmental sciences (*n* = 284, 30.5%), followed by public, environmental, and occupational health (*n* = 222, 23.8%), toxicology (*n* = 93, 9.99%), biochemistry and molecular biology (*n* = 66, 7.09%), engineering environmental (*n* = 43, 4.62%), chemistry analytical (*n* = 42, 4.51%), and multidisciplinary sciences (*n* = 41, 4.40%) (Fig. A.23).

### Keyword analysis

**Fig. 3:**
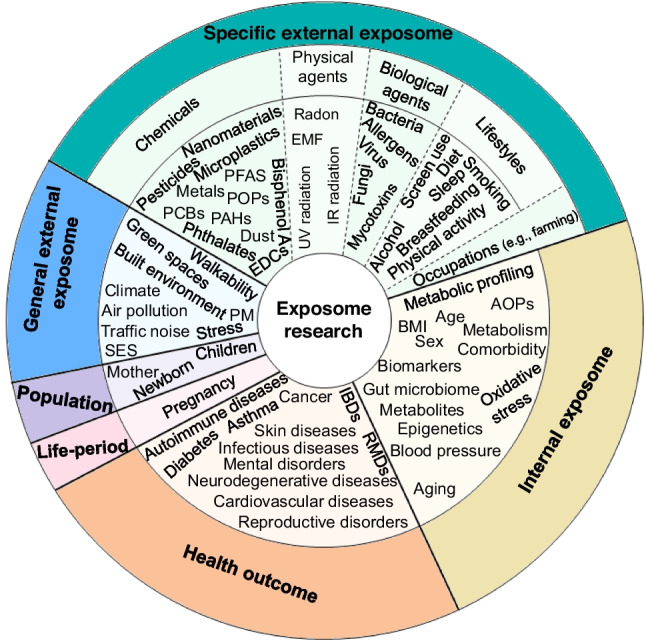
Most frequent keywords by exposome domains. AOP adverse outcome pathway, BMI body mass index, EDCs endocrine-disrupting chemicals, EMF electromagnetic field, IBD inflammatory bowel disease, IR infrared, PAH polycyclic aromatic hydrocarbon, PCB polychlorinated biphenyls PFAS per- and polyfluoroalkyl substances, POP persistent organic pollutant, PM particulate matter, RMD rheumatic and musculoskeletal disease, SES socio-economic status, UV ultraviolet

The human exposome publication keywords reflect the research conducted in the three exposome domains, with most research conducted in the internal and specific external domains (Fig. 3).

#### Most frequent keywords

A word cloud of the most frequent keywords is presented in Fig. A.24. The top 40 of the most frequent keywords were mentioned in at least 26 publications (2.79%) (Table A.18). The “exposome” was the most frequent keyword (57.9%), followed by “environment” (20.0%), “chemicals” (15.0%), “exposure” (13.7%), and “metabolomics” (10.8%). The frequency of the top five keywords increased drastically this past decade (Fig. A.25). “Exposome,” “exposure,” “environment,” “biomarkers,” “epidemiology,” “chemicals,” “metabolomics,” “reproductive disorders,” and “children” were the most frequent keywords that were mentioned in at least one publication for 12 years (not necessarily consecutive) (Fig. A.26). “Amyotrophic lateral sclerosis,” “risk factors,” “road traffic,” “extracellular vesicles,” “high-resolution mass spectrometry (HRMS),” and “epigenetics” were the keywords with the highest mean AGR (Fig. A.27).

The most frequently mentioned components of the exposome were the chemical exposome (*n* = 64, 6.9%), the internal exposome (*n* = 21), the urban exposome (*n* = 20), the public health exposome (*n* = 11), the social exposome (*n* = 11), the skin exposome (*n* = 9), the working life exposome (*n* = 9), the pregnancy exposome (*n* = 8), the blood exposome (*n* = 7), the personal exposome (*n* = 6), the endogenous exposome (*n* = 5), the farming exposome (*n* = 4), the eco-exposome (*n* = 3), the placental exposome (*n* = 3), the gero-exposome (*n* = 2), the athlete’s exposome (*n* = 2), the psychoexposome (*n* = 1), the environmental exposome (*n* = 1), the space exposome (*n* = 1), the viral exposome (*n* = 1), the fungal exposome (*n* = 1), the drug exposome (*n* = 1), the in utero exposome (*n* = 1), and the metal exposome (*n* = 1). Early-life exposures, including pregnancy, were the most commonly reported life stage (*n* = 206, 22.1%). In contrast, only 22 publications focused on working life and 12 on late-life exposures. Only one study considered digital exposure (e.g., length of mobile phone use) (Camacho et al. 2024).

Of the 389 (41.8%) publications reporting only mono-exposure keywords, most were related to chemicals (*n* = 226, 58.1%) (e.g., PFAS), followed by internal (*n* = 47, 12.1%), social (*n* = 41, 10.5%), biological (*n* = 21, 5.40%), dietary (*n* = 18, 4.62%), and lifestyle exposures (*n* = 18, 4.62%). In contrast, 309 (33.2%) publications addressed multi-exposure keywords, often combining multiple exposure domains. The most common combinations were chemical/chemical (*n* = 78), chemical/internal (*n* = 24), chemical/diet (*n* = 16), chemical/lifestyle (*n* = 13), and chemical/social factor (*n* = 13). Other combinations included diet/internal (*n* = 11), social factor/internal (*n* = 9), social factor/lifestyle (*n* = 7), chemical/biological agent (*n* = 7), chemical/urban environment (*n* = 7), chemical/physical agent (*n* = 8), chemical/pharmaceutical (*n* = 8), and social factor/urban environment (*n* = 7). Six studies focused on exposure mixtures using Bayesian kernel machine regression (BKMR) (Brydges et al. 2023; Guillien et al. 2021; Li et al. 2024; Papadopoulou et al. 2021; Sun et al. 2024; Wu et al. 2022b).

A total of 49 (5.26%) publications mentioned “questionnaire,” with only ten using validated ones (Dypås et al. 2023; Guo et al. 2024; Jedynak et al. 2021; Li et al. 2024; Prieto-Botella et al. 2022; Rejek and Misiak 2024a,b; Romera-Vilchez et al. 2022; Stratakis et al. 2020; van der Sloot et al. 2021).

Among top authors and funding bodies, keywords showing the most important positive growth over time included chemicals, metabolomics, cancer, air pollution, reproductive disorders, respiratory diseases, mental health disorders, omics, exposomics, and AI (Fig. A.28 and 29).

#### Keyword co-occurrence

Four clusters of keyword co-occurrence were identified using multiple correspondence analyses. One cluster (in green) centered on respiratory diseases, autoimmune diseases, skin diseases, microbiome, and climate change, while another cluster (in blue) focused on air pollution, physical factors, urban exposome, physical activity, stress, and cardiovascular diseases. Another cluster (in purple) centered on the chemical exposome and its associated analytical techniques, while the fourth cluster (in red) focused on various topics such as prenatal and neonatal exposures, reproductive disorders, mental health disorders, and statistical techniques (e.g., artificial intelligence (AI), modeling) (Fig. A.30).

**Fig. 4:**
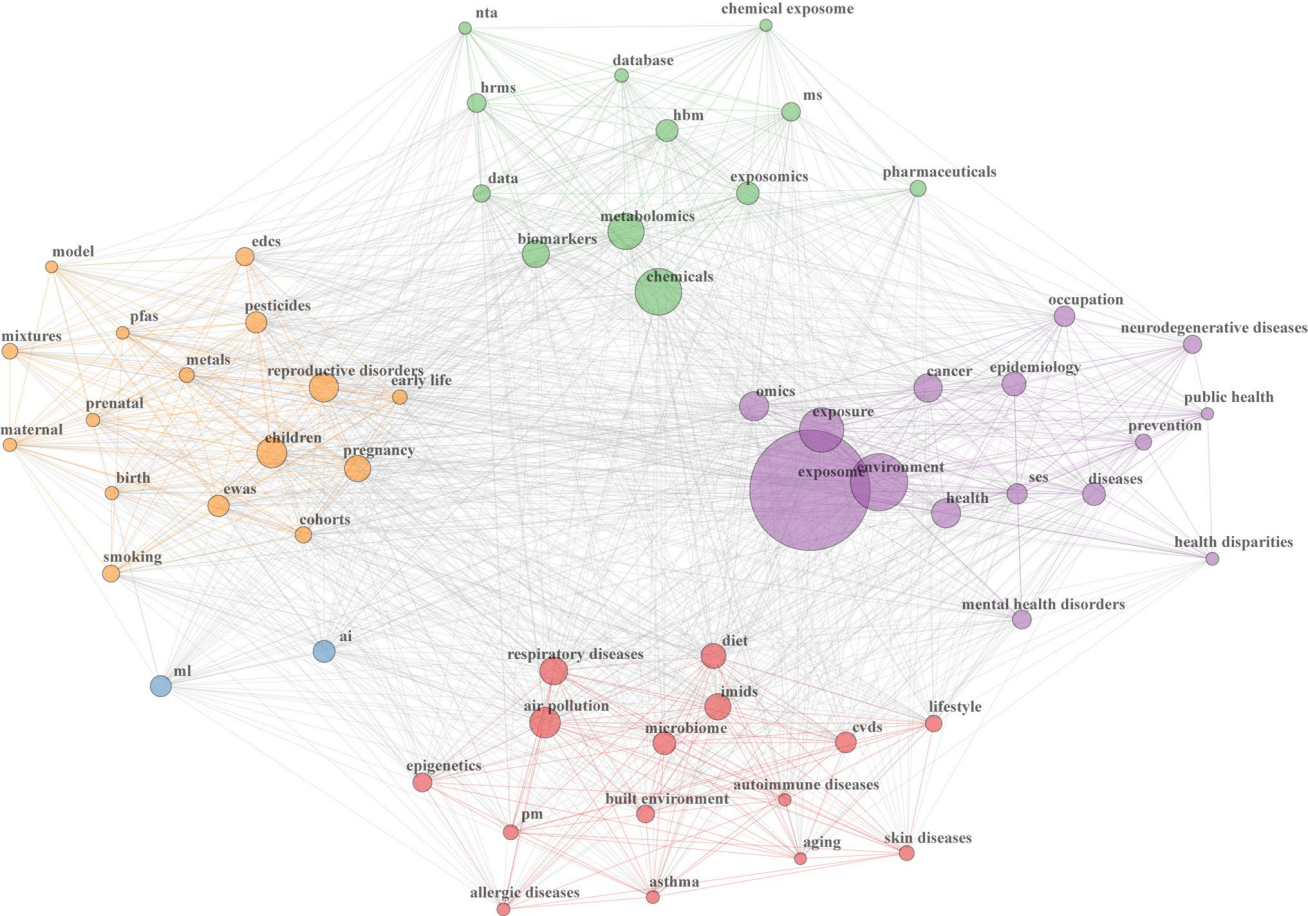
Keyword co-occurrence network. AI artificial intelligence, CVDs cardiovascular diseases, EDCs endocrine-disrupting chemicals, EWAS exposome-wide association study, HBM human biomonitoring, HRMS high-resolution mass spectrometry, IMIDs immune-mediated inflammatory diseases, ML machine learning, MS mass spectrometry, NTA non-targeted analysis, PFAS per- and polyfluoroalkyl substances, PM particulate matter, SES socio-economic status

A keyword co-occurrence network was also constructed, with node size corresponding to the frequency of keywords (Fig. 4). Five distinct clusters were identified: one (in red) on microbiome, air pollution, diet, lifestyle, built environment, epigenetics, aging, respiratory, skin, cardiovascular, and autoimmune diseases; one (in orange) on prenatal and neonatal exposure, reproductive disorders, pesticides, EDCs, metals, mixtures, and PFAS; one (in blue) on AI-related topics; one (in green) on the chemical exposome, exposomics, metabolomics, biomarkers, and other analytical techniques; and one (in purple) on cancer, neurodegenerative diseases, mental health disorders, occupation, prevention, epidemiology, and omics.

#### Thematic mapping—research hotspots

**Fig. 5:**
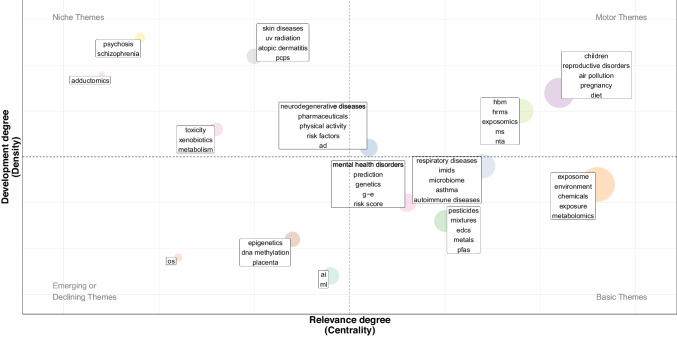
Thematic mapping. AD Alzheimer’s disease, AI artificial intelligence, DNA deoxyribonucleic acid, EDCs endocrine-disrupting chemicals, G-E gene-environment interaction, HBM human biomonitoring, HRMS high-resolution mass spectrometry, IMIDs immune-mediated inflammatory diseases, ML machine learning, MS mass spectrometry, NTA non-targeted analysis, OS oxidative stress, PCPs personal care products, PFAS per- and polyfluoroalkyl substances, UV ultraviolet

Figure 5 presents a thematic map (results of co-word analysis), where each theme (clusters of keywords) is characterized by density (the internal strength of a research theme) and centrality (the degree of interaction of a theme with other themes in the map) (Fig. 5).

There were three central and developed themes (motor themes in the upper-right quadrant), with one related to “exposomics” and analytical techniques (e.g., mass spectrometry), one to “reproductive disorders,” “children,” “pregnancy,” “diet” and “air pollution,” and one to “neurodegenerative diseases,” “pharmaceuticals,” “risk factors,” and “physical activity.”

Four transversal and underdeveloped themes were identified (basic themes in the lower-right quadrant): one focused on chemicals (e.g., pesticides, mixtures, EDCs, metals, PFAS); another on metabolomics; a third on microbiome, respiratory, and autoimmune diseases; and a fourth on mental health disorders, prediction, risk score, gene-environment interaction, and genetics.

Four peripheral but more developed themes were also found (niche themes in the upper-left quadrant): one on adductomics; another on skin diseases, UV radiation, and personal care products; a third on metabolism, toxicity, and xenobiotics; and a fourth on schizophrenia and psychosis.

Finally, three emerging or declining themes were identified (weakly developed and marginal themes in the lower-left quadrant): one on oxidative stress, one on epigenetics and DNA methylation, and one on AI and machine learning, which is close to becoming a basic theme.

#### Co-occurrence between PRFKs and specific health outcome

**Fig. 6:**
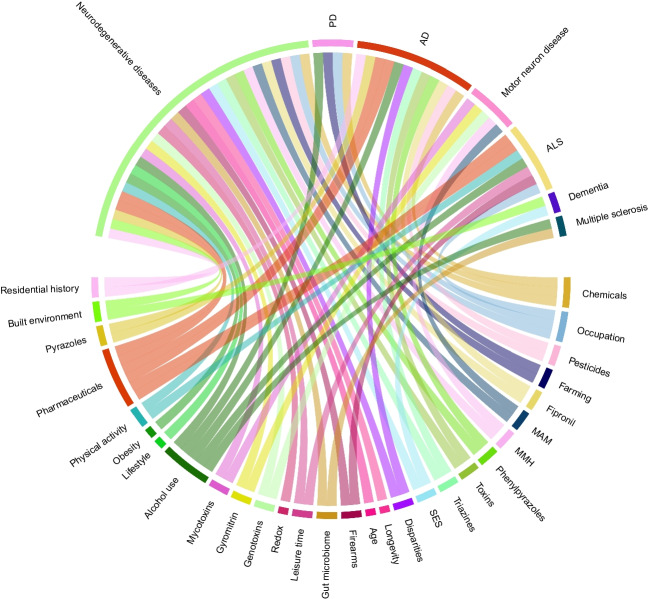
Chord diagram of keyword co-occurrence between potential risk factor and neurodegenerative disease keywords. AD Alzheimer’s disease, ALS amyotrophic lateral sclerosis, MAM methylazoxymethanol, MMH monomethylhydrazine, PD Parkinson’s disease, SES socio-economic status

There were 12 different types of cancer (e.g., leukemia) that co-occurred in publications with between one and 44 PRFKs (e.g., pesticides, air pollution) (Fig. A.31–A.33). Regarding neurodegenerative diseases (e.g., Alzheimer’s disease), there were seven disorders that co-occurred with between 2 and 30 PRFKs (Figs. 6 and A.34–A.35). Ten keywords related to mental disorders (e.g., depression) co-occurred with between 1 and 38 PRFKs (Fig. A.36–A.38), while nine keywords related to autoimmune diseases (e.g., Crohn’s disease) co-occurred with between 2 and 45 PRFKs (Fig. A.39–A.41). Six keywords related to cardiovascular diseases (e.g., stroke) co-occurred with between 2 and 42 PRFKs (Fig. A.42–A.44), while 14 keywords related to gastrointestinal diseases (e.g., enteropathy) co-occurred with between 1 and 18 PRFKs (Fig. A.45–A.47). Twelve keywords related to respiratory diseases (e.g., asthma) co-occurred with between 1 and 50 PRFKs (Fig. A.48–A.50), while 16 keywords related to reproductive disorders (e.g., infertility) co-occurred with between 1 and 52 PRFKs (Fig. A.51–A.53), and 15 keywords related to skin diseases (e.g., skin aging) co-occurred with between 1 and 34 PRFKs (Fig. A.54–A.56). Figure A57 presents the results for other health events (e.g., hospital admissions, mortality).

#### Research trends on exposome digital tools

Figure A.58 presents the top ten of the most frequent keywords related to digital tools by total number of years of appearance. Keywords were mainly related to statistical and computational techniques (e.g., BKMR, regression, spatial analysis), software (e.g., Python, R), AI (e.g., machine learning, deep learning, prediction, SHAP—Shapley Additive Explanations), and data (e.g., databases, sensors, wearables, smartphone data). Digital tool-related keywords were most commonly associated with studies on reproductive disorders, respiratory diseases, mental health issues, breast cancer, diabetes, and cardiovascular diseases (Fig. A.59).

## Discussion

### Main findings

This study provides a bibliometric analysis of human exposome publications from 2005 to 2024. The number of publications over time reflects the amount of interest that the human exposome research field is receiving and whether this field is growing over time. The exposome represents a critical driver of human health and disease, as shown by the increasing scholarly interest in the human exposome and related issues since 2010, which is consistent with published data (Haddad et al. 2019; Kiossoglou et al. 2017; Molina-García et al. 2022; Zhang et al. 2021). Consistent with previous studies, most human exposome studies were conducted in European countries (Haddad et al. 2019). Developed countries represented by the US, France, and the UK have undertaken most of the human exposome research (top tier), with close international cross-institutional collaboration. This is not surprising because most projects are funded by US and European sources (Haddad et al. 2019). However, countries reported in the publications only refer to the countries of affiliation of the authors, which does not necessarily correspond to where the research has been conducted. Research in low-income countries should be promoted and supported, as this could enable a better understanding of the human exposome and improve global health.

The most prolific authors were mostly from institutions in developed countries. Most of these authors were/are involved in one or several large-scale/international projects (Benjdir et al. 2021; Buck Louis et al. 2017; Guillien et al. 2023; Martinez et al. 2021; Ronsmans et al. 2022; Ruiz-Castell et al. 2023), such as the Human Early-Life Exposome (HELIX) (Maitre et al. 2022b; Vrijheid et al. 2014), Advancing Tools for Human Early Lifecourse Exposome Research and Translation (ATHLETE) (Vrijheid et al. 2021), EXPOsOMICS (Canali and Leonelli 2022; Vineis et al. 2017), Exposome Project for Health and Occupational Research (EPHOR) (Pronk et al. 2022; Schlünssen et al. 2023), or EXposome-Powered tools for healthy living in urbAN Settings (EXPANSE) (Gruzieva et al. 2022; Vlaanderen et al. 2021). HELIX is a European longitudinal population-based birth cohort study that aims to characterize the early-life exposome (Maitre et al. 2022b; Vrijheid et al. 2014). ATHLETE is devoted to developing a toolbox of exposome tools and a Europe-wide exposome cohort (Vrijheid et al. 2021). EXPOsOMICS aims to develop a novel approach to the assessment of exposure to high-priority environmental pollutants by characterizing the external and internal components of the exposome (Canali and Leonelli 2022; Vineis et al. 2017). EPHOR aims to advance knowledge on the complex working life exposome (Pronk et al. 2022; Schlünssen et al. 2023). EXPANSE is devoted to studying the impact of the urban exposome on the major contributors to Europe’s burden of disease (Gruzieva et al. 2022; Vlaanderen et al. 2021). These projects have led and will continue to lead to numerous publications, including many authors. This could explain why the authors working on these projects were among the most prolific and why some of them had a relatively low dominance factor and fractionalized frequency.

There were sex disparities between publications. As already reported in other fields, females were less likely to be senior authors (Chatterjee and Werner 2021; Sugimoto et al. 2019; Uribe-Bohorquez et al. 2023). Regarding primary authorship, there was almost equality between both sexes. Publications with female primary and senior authors had, most of the time, fewer citations than those written by males. This observation is consistent with previous works that reported lower levels of visibility and amplification for females in academic science compared with males (Chatterjee and Werner 2021; Sugimoto et al. 2019; Uribe-Bohorquez et al. 2023). These sex disparities were not explained by an impact journal effect. In addition, the human exposome is a broad field, so we cannot exclude that females wrote publications on topics that have smaller audiences and are less frequently cited. Other potential disparities regarding potential sociodemographic imbalances (e.g., race, ethnicity) could warrant further research. Similar to previous studies, the total number of publications was positively associated with GDP and population size (Wang and Zhao 2018).

Given the holistic aspect of the exposome concept, human exposome publications have been published in many journals, mainly in the environmental health, public health, exposure science, epidemiology, toxicology, and medical fields. Journals identified in this work are not dedicated primarily or specifically to the exposome but rather sponsored special issues or sections on this topic, which do not necessarily provide a tailored environment to facilitate dialogue on an international scale. There is one journal, named “Exposome,” that was launched in 2021 to focus specifically on the human exposome field. However, given its recent age, at the time of this work, it did not have an impact factor and was not indexed in WoSCC, which explains why no publication from this journal was identified in this work.

### Exposome genesis

Historically, the exposome term was first formulated in 2005 (Wild 2005). Other terms have sometimes been used to refer to the exposome, in particular ‘‘envirome’’, and ‘‘environome’’ (Anthony et al. 1995; Eaton 2002; Eaton and Harrison 1998; Kane et al. 2021; Price et al. 2022; Rose 2001). These terms were first coined before 2005. Anthony et al. (1995) defined the “envirome” as a complement to the human genome that refers to the “total ensemble of environments, both current and in earlier life, that affect the occurrence of psychiatric disturbances” (Anthony et al. 1995). Since then, the term “envirome” has been used in other publications, with several that were published before the work from Wild (2005) (Eaton 2002; Eaton and Harrison 1998; Rose 2001; Wild 2005). In 2002, Dr. William W. Eaton gave a broader definition of the “envirome” to include the entire ensemble of environmental factors (air pollution, water pollution, infections, and social life) over the life span (Eaton 2002). More recently, Price et al. (2022) proposed reattributing the exposome concept to “exclusively represent the totality of contact with external factors that a biological entity experiences.” They also introduced the term “functional exposomics” to describe the systematic study of exposure-phenotype interactions. Safarlou et al. (2024) suggested replacing the term exposome with “exposomics” to clarify the distinction between environment and exposome. They defined exposomics as “a research program in environmental health aimed at enabling a comprehensive and discovery-driven approach to identifying environmental determinants of human health.”

### Exposome concepts/components

According to Haddad et al. (2019), there is a lack of commonly accepted exposome language. Indeed, since 2005, many concepts have emerged within the human exposome framework to explore and focus on some specific components of the exposome (Buck Louis et al. 2017).

The “public health exposome” integrates information about endogenous and exogenous exposure in relation to health outcomes at both the individual and population health levels (Juarez et al. 2014). The “eco-exposome” is defined as the bidirectional influences between ecosystems and human exposures (Barouki et al. 2022; Juarez et al. 2014). The “endo-exposome” is the study of the inward effects arising from exposure to the biological receptor of an individual (Juarez et al. 2014; Nakamura et al. 2014; Valero et al. 2022). The “adductome” encompasses chemical exposures leading to the formation of DNA adducts contributing to carcinogenesis (Ananthakrishnan 2015). The “plasma exposome” provides a comprehensive capture of the systemic inflammatory network (Schisler et al. 2015). The “blood exposome” and “serum exposome” focus on blood and serum to characterize the exposome (Rappaport 2012a; Rappaport et al. 2014).

Some exposome components are restricted to specific exposure stressors. The “chemical exposome” focuses on chemical stressors/exposures in relation to health outcomes (Misra 2020; Petit 2022; Price et al. 2022). The “mineral exposome” encompasses dust exposure from environmental and occupational settings (Catinon et al. 2018). The “infectome” is used to measure lifetime exposure to infections (Ananthakrishnan 2015). The “pollutome” is the study of pollution effects on human health (Pini et al. 2023). The “social exposome” or “socio-exposome” studies the role of social environmental health determinants (Deguen et al. 2022; Gudi-Mindermann et al. 2023; Neufcourt et al. 2022). The “digital exposome” refers to the impact of exposure to digital technologies on human health (Hong 2024; Lopez-Campos et al. 2017; Martin-Sanchez et al. 2020; Mumtaz et al. 2023; Pagliaccio et al. 2024).

Other exposome components are restricted to specific exposure environments. The “occupational exposome” or “working life exposome” focuses on occupational stressors/exposures (Bonneterre et al. 2010; Faisandier et al. 2011; Pronk et al. 2022), along with the “spectrosome” which refers to the network/spectrum of multi-occupational exposures related to potential health outcomes (Bosson-Rieutort et al. 2018). The “farming exposome” or “agricultural exposome” focuses on all farming and related nonoccupational exposures (Petit and Vuillerme 2025; Petit et al. 2024a,b, 2025). The “urbanome” or “urban exposome” focuses on urban stressors (e.g., built environment) that can have an impact on human health (Morawska et al. 2019; Novak et al. 2023). This concept can be extended or generalize beyond urban environments to any location (“locusome,” from the Latin word locus, which means location). The “space exposome” refers to the effects of spaceflight on human health (Marchal et al. 2024).

Some exposome components are restricted to specific life periods, with, for instance, the “pregnancy exposome,” which refers to the study of environmental contaminants during pregnancy and continuing into early life (Chen et al. 2022; Robinson and Vrijheid 2015), or the “gero-exposome” for late-life exposures (Finch and Haghani 2021; Finch 2023).

Other exposome components focus on specific diseases (Celebi Sozener et al. 2022; Guillien et al. 2023; Hall et al. 2023). The “psychoexposome” considers psychosocial environmental exposures in the context of psychopathology (Gudi-Mindermann et al. 2023). The “gastrointestinal exposome” considers the integration of all elements (e.g., xenobiotics, host-derived endogenous elements) affecting the host’s health during the lifespan (Moon 2016). The “AD exposome” pertains to endogenous and exogenous environmental factors related to Alzheimer’s disease (Finch and Kulminski 2019), while the “neurol exposome” focuses on non-genetic factors that influence neurological disorders, including those that may be reflected in epigenetic changes (Tamiz et al. 2022). Finally, there is an exposome component restricted to skin health (Khmaladze et al. 2020; Krutmann et al. 2017; Molina-García et al. 2022).

The above-mentioned exposome components are not exhaustive but provide a plethora of evidence that there is a need for further refining of the human exposome framework and utility (Haddad et al. 2019) and that the exposome has inspired numerous researchers worldwide and is expected to influence environmental and public health research, practices, and policies (Barouki et al. 2022). The wide range of exposome components reflects the complexity of fully characterizing the exposome, which is complicated by the diversity and temporal and spatial variability of environmental factors (Barouki and Lévi 2024). However, these exposome divisions may sometimes represent the rebranding of existing approaches (Safarlou et al. 2024). While dividing the exposome into components can be valuable for developing specialized exposomics tools and methods, this approach must adhere to a discovery-driven methodology or include plans for integrating these tools into broader strategies for comprehensive exposure assessment (Safarlou et al. 2024).

### Research hotspots/trends

As previously reported, only a subset of the entire human exposome framework has been studied (Haddad et al. 2019). Keyword analysis revealed that human exposome studies mainly focused on air pollution, the environment, biomarkers, metabolomics, chemicals (e.g., PFAS, pesticides, EDCs, metals), early-life exposure, diet, non-targeted analysis (e.g., high-resolution mass spectrometry), and the microbiome. The most popular/frequent health outcomes investigated were cancer, respiratory diseases, cardiovascular diseases, reproductive diseases, and mental health disorders. Adductomics, skin diseases, metabolism, and psychosis were niche themes, while oxidative stress, epigenetics, DNA methylation, and AI represented emerging/declining themes, with AI close to becoming a basic theme. Social factors, physical agents, biological agents, occupational exposures, digital exposure (e.g., screen use), multi-exposure, and late-life exposure have received less attention, representing significant gaps in the literature that need to be addressed. However, recent years have seen growing interest in lifestyle, social, and built environment factors (Andrianou et al. 2021; Deguen et al. 2022; Gudi-Mindermann et al. 2023; Haddad et al. 2022; Morawska et al. 2019; Neufcourt et al. 2022), as well as in multi-exposure studies (Barbey et al. 2024). Climate change remains an understudied area in exposome research (Do et al. 2024). Additionally, there has been limited investigation into certain health outcomes, such as autoimmune diseases, neurodegenerative diseases, and musculoskeletal disorders. Few studies have explored the impact of policies on the exposome, such as public health measures during the COVID-19 pandemic (Andrianou et al. 2021; Matus et al. 2024). Notably, there is a lack of exposome-based public health intervention studies (Pearson et al. 2020), although several exposome-related studies have proposed policy recommendations (Andrianou et al. 2021; Matus et al. 2024; Sonnenschein et al. 2022; Vineis et al. 2023).

### Digital exposome toolbox

**Fig. 7:**
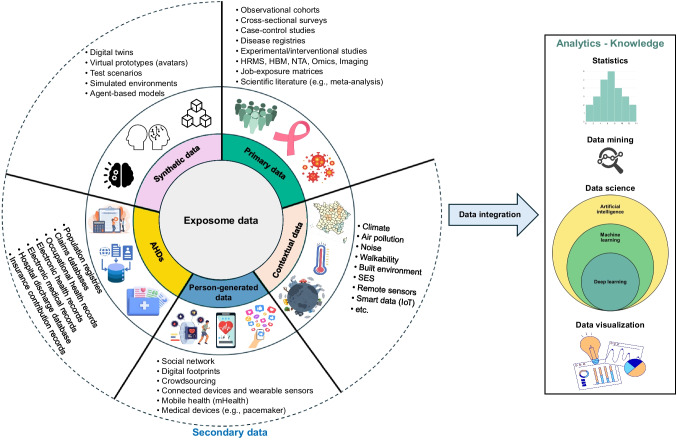
Digital exposome toolbox. AHDs administrative health databases, HBM human biomonitoring, HRMS high-resolution mass spectrometry, IoT Internet of Things, NTA non-targeted analysis, SES socio-economic status. Primary data are specifically collected and tailored for a particular public health research purpose and can be used either once or multiple times to achieve the same objective. They form the foundation of traditional public health policy and decision-making. In contrast, secondary data are not originally gathered for public health purposes, but they can complement primary data to enrich analysis and insights

Exposome data is currently dispersed across hard-to-find and difficult-to-access sources, highlighting the urgent need for openly accessible resources (Vrijheid et al. 2021). However, only four studies have specifically referred to FAIR (findable, accessible, interoperable, reusable) data principles (Ghosh et al. 2023; Martinez et al. 2021; Petit 2022; Vrijheid et al. 2021), underscoring the importance of adopting FAIR practices in exposome research (Chung et al. 2024; Schmitt et al. 2023). As the volume, variety, and veracity of exposome data increase (Fig. 7), the data becomes highly heterogeneous and high-dimensional, posing major challenges for management and analysis (Ghosh et al. 2023; Haddad et al. 2019; Maitre et al. 2022a; Marín et al. 2024; Vrijheid et al. 2021). Consequently, the traditional tools and methods used to integrate, manage, analyze, and visualize these vast and heterogeneous data sources may prove insufficient, requiring the adoption and application of new technologies and innovative statistical methods (Fig. 7). Therefore, there is a growing need for advanced statistical methods and data science approaches, such as BKMR and AI (Chung et al. 2024; Isola et al. 2024; Maitre et al. 2022a; Zhu et al. 2024). The integration of data science into public health is at the heart of an emerging field called “digital public health,” which seeks to use new types of data sources and technologies (Fig. 7) to provide the right intervention to the right population at the right time (Dolley 2018; Iyamu et al. 2024; Odone et al. 2019; Petit and Vuillerme 2025; Wienert et al. 2022).

To address the logistical and analytical challenges posed by exposome data—such as its heterogeneous nature (Fig. 7 and Fig. A.61) and the development of appropriate statistical techniques—digital health technologies and analytics have proliferated. These include sensors, mobile applications, wearables, electronic health records, databases, omics, non-targeted analysis, big data, and AI (Abdelzaher et al. 2022; Barouki et al. 2022; Buck Louis et al. 2017; Dennis et al. 2017; Descatha and Fadel 2023; Guillien et al. 2023; Haddad et al. 2019; Juarez et al. 2014; Kane et al. 2021; Kuijpers et al. 2021; Maitre et al. 2022a; Marín et al. 2024; Petit 2022; Pronk et al. 2022; Safarlou et al. 2023; Santos et al. 2020; Schmitt et al. 2023; Vineis et al. 2017; Vlaanderen et al. 2021; Vrijheid et al. 2021; Zhang et al. 2021). Exposome assessment primarily relies on objective approaches, such as passive data collection through sensor technology and non-targeted analyses (Kalia et al. 2022; Lai et al. 2024; Manz et al. 2023; Nguyen et al. 2022; Turner et al. 2017). However, many studies rely on self-reported questionnaire data, which is an inexpensive and effective approach to collecting data from a large population (Turner et al. 2017). Only four studies have reported using an exposome questionnaire (Akhtari et al. 2023; Claros et al. 2023; Gabriel et al. 2020; Lee et al. 2022), such as in the Personalized Environment and Genes Study (Lee et al. 2022; Lloyd et al. 2024a,b), though none are currently validated. The lack of validated questionnaires may stem from the challenge of assessing the vast array of potentially causative exposures (Rappaport 2012b). As previously emphasized, there is a pressing need for improved questionnaire data (Vineis et al. 2020), such as a validated and standardized exposome questionnaire.

The use and analysis of routinely collected real-world data, including contextual, person-generated, and clinical data (Fig. 7 and Fig. A.61), hold major potential to advance human health. These data can provide real-time insights that support health surveillance and guide future precision prevention and medicine strategies (Kalia et al. 2022; Schmitt et al. 2023; Subramanian et al. 2020; Tagliaferro et al. 2024; Zhang et al. 2021). Several studies have developed and/or used sensors (Doherty et al. 2021; Pronk et al. 2022), such as a personal exposure monitoring kit composed of a smartphone, accelerometer, UV dosimeter, and air pollution monitors (Donaire-Gonzalez et al. 2019), or a flexible individual sensor device combining environmental and biomarker toolkits (Benjdir et al. 2021).

Among the 40 (4.30%) publications utilizing AI, 12 focused on identifying predictors of health outcomes, including type 2 diabetes (Atehortúa et al. 2023; Ohanyan et al. 2022a), mental health (van Kamp et al. 2021), body mass index (BMI) (Ohanyan et al. 2022b, 2024), physical activity (Lee et al. 2023), amyotrophic lateral sclerosis (Rotem et al. 2024), self-perceived health (Hoekstra et al. 2023; Loef et al. 2022), healthy aging (Loef et al. 2023), dementia (Camacho et al. 2024), and childhood outcomes (Lester et al. 2024). Random forest and XGBoost (extreme gradient boosting) were the most commonly applied machine learning algorithms, with SHAP (Lundberg et al. 2020; Zhu et al. 2024) and partial dependence plots (PDP) (Hoekstra et al. 2023; Zhu et al. 2024) being the preferred methods for model interpretation (explainable AI). As previously reported, most authors applied supervised or unsupervised ML models, while few used deep learning (Isola et al. 2024). The application of AI to identify exposomic risk factors for health outcomes represents a promising and innovative research avenue (Isola et al. 2024). However, major challenges remain and need to be addressed to facilitate AI application and advance its reliability and impact, in particular regarding data integration, standardization, and the appropriate choice of the AI model (Isola et al. 2024). The integration and application of AI and data science in exposome research hold great promise and have the potential to support the development of informed, evidence-based, effective, practical, and personalized prevention, decision, and intervention tools and strategies (Isola et al. 2024).

Exposome Risk Scores (ERS), which summarize the effects of multiple exposomic risk factors for a specific health outcome at the individual level, are another emerging area of study (Guimbaud et al. 2024; Hu et al. 2023; Pries et al. 2019, 2021a, b). Inspired by polygenic risk scores (Torkamani et al. 2018), ERS allow for the identification of modifiable risk factors crucial for prevention and healthcare decision-making. Twenty-four studies (2.58%) have reported the calculation or use of ERS, predominantly for mental health disorders such as schizophrenia and psychosis (Cuesta et al. 2023, 2024; Erzin et al. 2021, 2023; Fusar-Poli et al. 2023; Mas et al. 2020; Paquin et al. 2023; Pries et al. 2019, 2020a,b, 2021a,b, 2022; Rejek and Misiak 2024a,b; Smith et al. 2023) or depression (Farré et al. 2024). ERS have also been applied, to a lesser extent, for diabetes (Akhtari et al. 2023), cardiovascular disorders (Atehortúa et al. 2023; Ferrari et al. 2022), well-being (van de Weijer et al. 2024), and childhood outcomes (Guimbaud et al. 2024; Healy et al. 2024; Smith et al. 2023; Visoki et al. 2024). AI-based ERS computation has been explored in two studies on schizophrenia (Pries et al. 2019, 2021a) and one on child outcomes (Guimbaud et al. 2024).

The environmental impact of data-driven research is a growing concern, as computational processes contribute to greenhouse gas emissions and climate change, which in turn affect health (Amini et al. 2024; Barouki 2024; Di Napoli et al. 2022; Filippini et al. 2024; Jabakhanji et al. 2022; Rocque et al. 2021). This calls for a reevaluation of research methodologies to reduce environmental footprints (Attari et al. 2016; Capet and Aumont 2024; Feigin et al. 2023; Gratiot et al. 2023; Mariette et al. 2022; Schrems and Upham 2020). Despite its increasing importance in public health, ecologically responsible research (e.g., data reuse, green AI) in the exposome domain remains largely underdeveloped (Aleixandre-Benavent et al. 2020; Anthony et al. 2020; Capet and Aumont 2024; Durgan et al. 2023; Feigin et al. 2023; Gratiot et al. 2023; Henderson et al. 2020). No studies have adopted environmentally sustainable research approaches, such as green AI, in exposome research. Green AI, which seeks to generate results without increasing—and ideally reducing—computational costs (Chaperon et al. 2024; Moyano-Fernández et al. 2024; Schwartz et al. 2019; Ueda et al. 2024), remains an underdeveloped field.

While advances have been made, ethical concerns exist, and standardization remains lacking in exposome data, health outcome assessments, and analytical methods and tools (e.g., personal sensors, wearables, remote sensors, and harmonized job exposure matrix) (Haddad et al. 2019; Isola et al. 2024; Pronk et al. 2022; Safarlou et al. 2023; Schmitt et al. 2023; Turner et al. 2018; Zhang et al. 2021). Issues such as geographical, sex, and ethnic disparities further complicate data availability, analysis, and interpretation (Bucher et al. 2023; Fayet et al. 2024; Nwanaji-Enwerem et al. 2021). Addressing these challenges requires efforts to promote data integration and reuse (Martinez et al. 2021; Zhang et al. 2021). Numerous initiatives have emerged to tackle some of these limitations, including data challenges to address high-dimensionality data (Maitre et al. 2022a); R packages for exposome data management, analysis, and visualization (Chung et al. 2024; He and Patel 2023; Hernandez-Ferrer et al. 2019); seminars on data analysis (Grady et al. 2023); the development of bioinformatics tools (Ronsmans et al. 2022; Vrijheid et al. 2021); and the development of a data management plan and platform (Ghosh et al. 2023; Martinez et al. 2021). In addition, the EXPOsOMICS project consultation with stakeholders highlighted the need for standardized methodologies, improved data sharing, and integration to demonstrate the added value of exposome science in addressing key policy questions (Turner et al. 2018). Collaborative efforts involving stakeholders, researchers, and communities are critical to ensure that exposome research aligns with public health priorities (Lebow-Skelley et al. 2022; Pearson et al. 2020; Stingone et al. 2023; van Kamp et al. 2021). For example, the HERCULES Exposome Research Center aimed to engage local communities in Atlanta by incorporating community environmental health and exposome-related concerns into scientific research. This was achieved through the creation of a community grant program (Pearson et al. 2020). The program successfully identified local environmental health issues while delivering tangible benefits to both the grantees and the communities they serve (Pearson et al. 2020). Research that actively involves the community has been shown to build trust, increase the relevance and applicability of findings, address equity and social justice concerns, and enhance both community knowledge and capacity (Pearson et al. 2020). Governments and institutions must also play an active role in supporting evidence-based policy-making (Barouki and Lévi 2024; Whitsel et al. 2019). Ultimately, interdisciplinary collaboration and international cooperation are essential to guide effective public health interventions (Cui et al. 2016; Fayet et al. 2024; Pearson et al. 2020). For instance, the Human Exposome Assessment Platform (HEAP) seeks to “enable global collaborative research on exposure to cost-effective health interventions” (Martinez et al. 2021). In addition, there is a need to integrate the concept of the exposome into the daily practices of healthcare professionals, as well as to raise awareness among patients and citizens to encourage both local and individualized prevention, especially since the concept of the exposome remains largely unknown to the general public and many healthcare professionals (Barouki and Lévi 2024; Bíró et al. 2018). Integrating the exposome into clinical practice aims to equip healthcare professionals with the necessary knowledge to enhance primary and secondary prevention through “6P” medicine: personalized, preventive, predictive, participatory, evidence-based, and patient-centered (Barouki and Lévi 2024; Eigenmann 2023). In this context, the development of technological innovations, such as decision-support tools, is crucial (Muhiyaddin et al. 2020). The goal of such technological innovations is to promote early interventions and guide toward exposomes that are most favorable to health and well-being.

The digital exposome toolbox (Fig. 7) holds great potential to inform precision decision-making and underpin evidence-based, cost-effective public health policies, ensuring the right interventions for the right populations at the right time (Bosward et al. 2022; Canfell et al. 2022a, b, c; Dolley 2018; Iyamu et al. 2021; Kalia et al. 2022; Khoury et al. 2016; Odone et al. 2019; Schmitt et al. 2023; Velmovitsky et al. 2021; Wienert et al. 2022). These technologies could also foster green (i.e., environmentally sustainable) innovations, such as the secondary use (or reuse) of data, to scale up effective interventions and ensure long-term population health benefits.

### Strengths and limitations

To our knowledge, no bibliometric analysis has been conducted to comprehensively describe the entire human exposome body of the literature so far. Only one bibliometric analysis was conducted on the exposome, focusing mainly on the skin exposome (Molina-García et al. 2022). However, the exposome research field was only briefly described based on 910 documents published from 2005 to September 30, 2021 (Molina-García et al. 2022). In our analysis, we included almost the same number of publications as Molina-García et al. (2022). However, compared to the work from Molina-García et al. (2022), we considered three additional years, and we screened publication titles and abstracts following PRISMA guidelines to ensure search accuracy. There should be fewer potential errors inherent to the extraction and nature of bibliometric data than in traditional bibliometric analyses because all terms (e.g., authors, keywords) were checked, cleaned, and harmonized (e.g., use of one unique term for a given entity) before conducting the analysis. While this approach is time-consuming, it allows for a more accurate analysis. Indeed, if the same entity had several denominations, it could lead to possible underestimates (e.g., number of publications).

Several limitations of the study warrant careful consideration. One of the main limitations is the use of a single bibliographic database (WoSCC). However, WoSCC has a broader coverage than other databases, with more consistent, reliable, high-quality, and standardized metadata, and is the most widely used database for bibliometric analysis (Lu and Ni 2022; Yin et al. 2022). Because of the existence of a broad range of exposome concepts/components, along with the non-systematic use of the exposome term in exposome studies (Haddad et al. 2019), we cannot exclude the possibility that publications with a methodology that abides by the exposome definition have been excluded, as previously acknowledged (Haddad et al. 2019). However, our search strategy was similar to other reviews on the human exposome (Haddad et al. 2019; Neufcourt et al. 2022; Safarlou et al. 2023). Only one author conducted the bibliometric data search, retrieval, management, and analysis. Therefore, it is possible that some human exposome studies may have been missed. Two independent reviewers may have decreased the chance of systemic error and bias. Sex was assigned using an external database, which may have resulted in misclassification. To limit this bias, sex was assigned manually for a low probability of accuracy. Last authorship may not perfectly match senior authorship. Shortcomings inherent to bibliometric analysis cannot be excluded. Indeed, a potential bias regarding authorship may occur if different authors have the same name or if authors use different names over time in their publications. A solution to overcome this limitation would be to use a unique author identifier (e.g., ORCID number), which was not available. Co-authorship (e.g., primary and senior) could not be identified, which is a limitation. Self-citation also represents a blind spot for citation analysis. We cannot exclude a potential Matthew effect that could affect publication citation (Uribe-Bohorquez et al. 2023). Despite the aforementioned limitations, we are confident that our findings can provide an effective perspective on the human exposome from a global insight, with a complete picture of what has been published until now.

## Conclusions

Exposome research is a pivotal component of digital public health, offering a pathway to developing accessible, affordable, integrated, scalable, and sustainable solutions that promote health and well-being across populations, regardless of geographic location or socio-economic status. Our findings provide a framework for scientists, policymakers, administrators, funding bodies, and other stakeholders to enhance their understanding and chart the future course of exposome research. The exposome will continue to inspire research across disciplines, shaping environmental and public health policies and practices as they transition from curative approaches to precision prevention and 6P medicine.

## Supplementary Information

Below is the link to the electronic supplementary material.

Supplementary file1 (DOCX 11549 KB)

Supplementary file2 (XLSX 96 KB)

## Data Availability

The datasets generated and/or analyzed during the current study are available from the corresponding author upon reasonable request.

## Abbreviations

AD: Alzheimer’s disease
AGEIS: Autonomy, Gerontology, E-health, Imaging & Society (Autonomie, Gérontologie, E-santé, Imagerie et Société in French)
AGR: Annual growth rate
AI: Artificial intelligence
ALS: Amyotrophic lateral sclerosis
ANR: French National Research Agency (Agence Nationale de la Recherche in French)
AOPs: Adverse outcome pathways
ATHLETE: Advancing Tools for Human Early Lifecourse Exposome Research and Translation
BKMR: Bayesian kernel machine regression
BMI: Body mass index
CIBER: Spanish Biomedical Research Center in Epidemiology and Public Health (Centro de Investigación Biomédica en Red in Spanish)
CNRS: Centre National de la Recherche Scientifique (French National Centre for Scientific Research)
CVD: Cardiovascular disease
DF: Dominance factor
DNA: Deoxyribonucleic acid
EDCs: Endocrine-disrupting chemicals
EMF: Electromagnetic field
EPHOR: Exposome project for health and occupational research
ERS: Exposome risk score
EXPANSE: Exposome-powered tools for healthy living in urban settings
EWAS: Exposome-wide association study
FAIR: Findable, accessible, interoperable, reusable
GDP: Gross domestic product
GIS: Geography information system
GWAS: Genome-wide association study
HBM: Human biomonitoring
HELIX: Human early-life exposome
HRMS: High-resolution mass spectrometry
IBD: Inflammatory bowel diseases
ICMJE: International Committee of Medical Journal Editors
IMIDs: Immune-mediated inflammatory diseases
INSERM: French National Institute of Health and Medical Research (Institut National de la Santé et de la Recherche Médicale in French)
IR: Infrared
MAM: Methylazoxymethanol
MIAI: Multidisciplinary Institute in Artificial Intelligence
ML: Machine learning
MMH: Monomethylhydrazine
MS: Mass spectrometry
NIEHS: National Institute of Environmental Health Sciences
NIH: National Institutes of Health
NTA: Non-targeted analysis
ORCID: Open researcher and contributor ID
OS: Oxidative stress
PAHs: Polycyclic aromatic hydrocarbons
PCB: Polychlorinated biphenyls
PD: Parkinson’s disease
PDP: Partial dependence plot
PFAS: Per- and polyfluoroalkyl substances
POPs: Persistent organic pollutants
PM: Particulate matter
PRFK: Potential risk factor keyword
PRISMA: Preferred reporting items for systematic reviews and meta-analyses
R&D: Research and development
RMD: Rheumatic and musculoskeletal disease
SES: Socioeconomic status
SHAP: Shapley additive explanations
UGA: Université Grenoble Alpes
UK: United Kingdom
US: United States
UV: Ultraviolet
WoSCC: Web of Science core collection
XGBoost: Extreme gradient boosting
