# Supplementary material for: Global research trends on the human exposome: a bibliometric analysis (2005–2024)
Source: Environ Sci Pollut Res Int. 2025 Mar 8;32(13):7808–33. doi: 10.1007/s11356-025-36197-7 (PMC11953191; doi:10.1007/s11356-025-36197-7)
Supplement: Supplementary file 1 — Supplementary file1 (DOCX 11549 KB) [file 11356_2025_36197_MOESM1_ESM.docx]

**Supplementary Information**

**Title**

Global research trends on the Human Exposome: a bibliometric analysis (2005-2024)

**Authors**

Pascal Petit^1,*^, PhD; Nicolas Vuillerme^1,2^, PhD

^1^ Univ. Grenoble Alpes, AGEIS, 38000 Grenoble, France

^2^ Institut Universitaire de France, Paris, France

**Correspondence (present address)**

⁎ Corresponding author at: Pascal Petit. Laboratoire AGEIS – Université Grenoble Alpes. Bureau 315. Bâtiment Jean Roget. UFR de Médecine. Domaine de La Merci. 38706 La Tronche Cedex, France.

E-mail address: [pascal.petit@univ-grenoble-alpes.fr](mailto:pascal.petit@univ-grenoble-alpes.fr) (P. Petit). Telephone: + 33 4 76 63 71 04.

**Table of contents**

[A.1. Preferred Reporting Items for Systematic reviews and Meta-Analyses extension for Scoping Reviews (PRISMA-ScR) Checklist. 4](#_Toc187922322)

[A.2. Bibliometric index metrics 5](#_Toc187922323)

[A.3. The BIBLIO checklist for reporting the bibliometric reviews of the biomedical literature 6](#_Toc187922324)

[Fig.A.1: PRISMA flow chart depicting the literature search and the evaluation process for finding relevant records 7](#_Toc187922325)

[Fig.A.2: Number of publications per document type 8](#_Toc187922326)

[Fig.A.3: Timeline of the first 10 publications published on the human exposome 9](#_Toc187922327)

[Fig.A.5: Map of the number of publications per country 10](#_Toc187922328)

[Fig.A.6: The most productive countries 11](#_Toc187922329)

[Fig.A.7: Scientific productivity – number of publications per year for the US 12](#_Toc187922330)

[Fig.A.8: Scientific productivity – number of publications per year for France 13](#_Toc187922331)

[Fig.A.9: Scientific productivity – number of publications per year for the UK 14](#_Toc187922332)

[Fig.A.10: Scientific productivity – number of publications per year for Spain 15](#_Toc187922333)

[Fig.A.11: Scientific productivity – number of publications per year for the Netherlands 16](#_Toc187922334)

[Fig.A.12: Number of average citations per document and subscription types 17](#_Toc187922335)

[Fig.A.13: Most investigated exposures by the top 20 most prolific authors 18](#_Toc187922336)

[Fig.A.14: Most investigated outcomes by the top 20 most prolific authors 19](#_Toc187922337)

[Fig.A.15: Total number of publications per year, authorship, and sex 20](#_Toc187922338)

[Fig.A.16: Total number of citations per year, authorship, and sex 21](#_Toc187922339)

[Fig.A.17: Total number of publications per year, authorship, and sex (considering only rank 1 journals) 22](#_Toc187922340)

[Fig.A.18: Total number of citations per year, authorship, and sex (considering only rank 1 journals) 23](#_Toc187922341)

[Fig.A.19: Total number of publications per year, authorship, and sex (considering only journals with impact factor ≥ 5) 24](#_Toc187922342)

[Fig.A.20: Total number of citations per year, authorship, and sex (considering only journals with impact factor ≥ 5) 25](#_Toc187922343)

[Fig.A.21: Total number of publications per year, authorship, and sex (considering only journals with impact factor ≥ 10) 26](#_Toc187922344)

[Fig.A.22: Total number of citations per year, authorship, and sex (considering only journals with impact factor ≥ 10) 27](#_Toc187922345)

[Fig.A.23: Tree map of the top 20 research topics/areas 28](#_Toc187922346)

[Fig.A.24: Word cloud of the most frequent keywords 29](#_Toc187922347)

[Fig.A.25: Top 5 of the most frequent keywords – growth trends 30](#_Toc187922348)

[Fig.A.26: Top ten of the most popular keywords by total number of years of appearance 31](#_Toc187922349)

[Fig.A.27: The top 40 keywords with the highest mean annual growth rate and their total number of citations 32](#_Toc187922350)

[Fig.A.28: Keywords with the most important positive growth over time among top authors, countries, funding bodies, institutions, and journals 33](#_Toc187922351)

[Fig.A.29: Exposure and health outcome-related keywords with the most important positive growth over time among top funding bodies 34](#_Toc187922352)

[Fig.A.30: Conceptual structure map of the most frequent keywords made with multiple correspondence analysis 35](#_Toc187922353)

[Fig.A.31: Chord diagram of keyword co-occurrence between potential risk factor and cancer keywords 36](#_Toc187922354)

[Fig.A.32: Heatmap of keyword co-occurrence between potential risk factor and cancer keywords 37](#_Toc187922355)

[Fig.A.33: Keyword co-occurrence network between potential risk factor and cancer keywords 38](#_Toc187922356)

[Fig.A.34: Heatmap of keyword co-occurrence between potential risk factor and neurodegenerative disease keywords 39](#_Toc187922357)

[Fig.A.35: Keyword co-occurrence network between potential risk factor and neurodegenerative disease keywords 40](#_Toc187922358)

[Fig.A.36: Chord diagram of keyword co-occurrence between potential risk factor and mental disorder keywords 41](#_Toc187922359)

[Fig.A.37: Heatmap of keyword co-occurrence between potential risk factor and mental disorder keywords 42](#_Toc187922360)

[Fig.A.38: Keyword co-occurrence network between potential risk factor and mental disorder keywords 43](#_Toc187922361)

[Fig.A.39: Chord diagram of keyword co-occurrence between potential risk factor and autoimmune disease keywords 44](#_Toc187922362)

[Fig.A.40: Heatmap of keyword co-occurrence between potential risk factor and autoimmune disease keywords 45](#_Toc187922363)

[Fig.A.41: Keyword co-occurrence network between potential risk factor and autoimmune disease keywords 46](#_Toc187922364)

[Fig.A.42: Chord diagram of keyword co-occurrence between potential risk factor and cardiovascular disease keywords 47](#_Toc187922365)

[Fig.A.43: Heatmap of keyword co-occurrence between risk potential factor and cardiovascular disease keywords 48](#_Toc187922366)

[Fig.A.44: Keyword co-occurrence network between potential risk factor and cardiovascular disease keywords 49](#_Toc187922367)

[Fig.A.45: Chord diagram of keyword co-occurrence between potential risk factor and gastrointestinal disease keywords 50](#_Toc187922368)

[Fig.A.46: Heatmap of keyword co-occurrence between potential risk factor and gastrointestinal disease keywords 51](#_Toc187922369)

[Fig.A.47: Keyword co-occurrence network between potential risk factor and gastrointestinal disease keywords 52](#_Toc187922370)

[Fig.A.48: Chord diagram of keyword co-occurrence between potential risk factor and respiratory disease keywords 53](#_Toc187922371)

[Fig.A.49: Heatmap of keyword co-occurrence between potential risk factor and respiratory disease keywords 54](#_Toc187922372)

[Fig.A.50: Keyword co-occurrence network between potential risk factor and respiratory disease keywords 55](#_Toc187922373)

[Fig.A.51: Chord diagram of keyword co-occurrence between potential risk factor and reproductive disease keywords 56](#_Toc187922374)

[Fig.A.52: Heatmap of keyword co-occurrence between potential risk factor and reproductive disease keywords 57](#_Toc187922375)

[Fig.A.53: Keyword co-occurrence network between potential risk factor and reproductive disease keywords 58](#_Toc187922376)

[Fig.A.54: Chord diagram of keyword co-occurrence between potential risk factor and skin disease keywords 59](#_Toc187922377)

[Fig.A.55: Heatmap of keyword co-occurrence between potential risk factor and skin disease keywords 60](#_Toc187922378)

[Fig.A.56: Keyword co-occurrence network between potential risk factor and skin disease keywords 61](#_Toc187922379)

[Fig.A.57: Chord diagram of keyword co-occurrence between potential risk factor and several health event keywords 62](#_Toc187922380)

[Fig.A.58: Top 10 of the most frequent keyword by periods of time 63](#_Toc187922381)

[Fig.A.59: Most frequent health outcome-related keywords that co-occurred with digital tool-related keywords 64](#_Toc187922382)

[Fig.A.60: Strengths and limitations of exposome data 65](#_Toc187922383)

[Table A.1: Criteria used for the publication selection 66](#_Toc187922384)

[Table A.2: List of all publications included 67](#_Toc187922385)

[Table A.3: Main characteristics of the included publications 68](#_Toc187922386)

[Table A.4: The most productive countries in the human exposome research field 69](#_Toc187922387)

[Table A.5: Top 30 of the most cited publications in the human exposome research field 71](#_Toc187922388)

[Table A.6: Top 20 of the most active journals in the human exposome research field 72](#_Toc187922389)

[Table A.7: Top 30 of the most prolific authors in the human exposome research field 73](#_Toc187922390)

[Table A.8: Number of publications and citations over time, stratified by authorship and sex 74](#_Toc187922391)

[Table A.9: Total number of publications and citations by primary and senior authorship pairs 74](#_Toc187922392)

[Table A.10: Number of publications and citations over time, stratified by authorship and sex, considering only rank 1 journals 75](#_Toc187922393)

[Table A.11: Total number of publications and citations by primary and senior authorship pairs, considering only rank 1 journals 75](#_Toc187922394)

[Table A.12: Number of publications and citations over time, stratified by authorship and sex, considering only journals with impact factor ≥ 5 76](#_Toc187922395)

[Table A.13: Total number of publications and citations by primary and senior authorship pairs, considering only journals with impact factor ≥ 5 76](#_Toc187922396)

[Table A.14: Number of publications and citations over time, stratified by authorship and sex, considering only journals with impact factor ≥ 10 77](#_Toc187922397)

[Table A.15: Total number of publications and citations by primary and senior authorship pairs, considering only journals with impact factor ≥ 10 77](#_Toc187922398)

[Table A.16: Top 20 of the most active institutions in the human exposome research field 78](#_Toc187922399)

[Table A.17: Top 20 of the most active funding bodies in the human exposome research field 79](#_Toc187922400)

[Table A.18: Top 40 of the most frequent keywords in the human exposome publications 80](#_Toc187922401)

[References 81](#_Toc187922402)

# **A.1. Preferred Reporting Items for Systematic reviews and Meta-Analyses extension for Scoping Reviews (PRISMA-ScR) Checklist.**

| **SECTION** | **ITEM** | **PRISMA-ScR CHECKLIST ITEM** | **REPORTED ON PAGE #** |
| --- | --- | --- | --- |
| **TITLE** | | | |
| Title | 1 | Identify the report as a scoping review. | 1 |
| **ABSTRACT** | | | |
| Structured summary | 2 | Provide a structured summary that includes (as applicable): background, objectives, eligibility criteria, sources of evidence, charting methods, results, and conclusions that relate to the review questions and objectives. | 2 |
| **INTRODUCTION** | | | |
| Rationale | 3 | Describe the rationale for the review in the context of what is already known. Explain why the review questions/objectives lend themselves to a scoping review approach. | 6-8 |
| Objectives | 4 | Provide an explicit statement of the questions and objectives being addressed with reference to their key elements (e.g., population or participants, concepts, and context) or other relevant key elements used to conceptualize the review questions and/or objectives. | 8 |
| **METHODS** | | | |
| Protocol and registration | 5 | Indicate whether a review protocol exists; state if and where it can be accessed (e.g., a Web address); and if available, provide registration information, including the registration number. | 9 |
| Eligibility criteria | 6 | Specify characteristics of the sources of evidence used as eligibility criteria (e.g., years considered, language, and publication status), and provide a rationale. | 8-9 |
| Information sources* | 7 | Describe all information sources in the search (e.g., databases with dates of coverage and contact with authors to identify additional sources), as well as the date the most recent search was executed. | 8-11 |
| Search | 8 | Present the full electronic search strategy for at least 1 database, including any limits used, such that it could be repeated. | 8 |
| Selection of sources of evidence† | 9 | State the process for selecting sources of evidence (i.e., screening and eligibility) included in the scoping review. | 8-11 |
| Data charting process‡ | 10 | Describe the methods of charting data from the included sources of evidence (e.g., calibrated forms or forms that have been tested by the team before their use, and whether data charting was done independently or in duplicate) and any processes for obtaining and confirming data from investigators. | 9-11 |
| Data items | 11 | List and define all variables for which data were sought and any assumptions and simplifications made. | 9-11 |
| Critical appraisal of individual sources of evidence§ | 12 | If done, provide a rationale for conducting a critical appraisal of included sources of evidence; describe the methods used and how this information was used in any data synthesis (if appropriate). | NA |
| Synthesis of results | 13 | Describe the methods of handling and summarizing the data that were charted. | 10, 11 |
| **RESULTS** | | | |
| Selection of sources of evidence | 14 | Give numbers of sources of evidence screened, assessed for eligibility, and included in the review, with reasons for exclusions at each stage, ideally using a flow diagram. | 11, supplementary materials |
| Characteristics of sources of evidence | 15 | For each source of evidence, present characteristics for which data were charted and provide the citations. | 11-24, supplementary materials |
| Critical appraisal within sources of evidence | 16 | If done, present data on critical appraisal of included sources of evidence (see item 12). | NA |
| Results of individual sources of evidence | 17 | For each included source of evidence, present the relevant data that were charted that relate to the review questions and objectives. | 11-24, supplementary materials |
| Synthesis of results | 18 | Summarize and/or present the charting results as they relate to the review questions and objectives. | 11-24, supplementary materials |
| **DISCUSSION** | | | |
| Summary of evidence | 19 | Summarize the main results (including an overview of concepts, themes, and types of evidence available), link to the review questions and objectives, and consider the relevance to key groups. | 25-27 |
| Limitations | 20 | Discuss the limitations of the scoping review process. | 34, 35 |
| Conclusions | 21 | Provide a general interpretation of the results with respect to the review questions and objectives, as well as potential implications and/or next steps. | 35 |
| **FUNDING** | | | |
| Funding | 22 | Describe sources of funding for the included sources of evidence, as well as sources of funding for the scoping review. Describe the role of the funders of the scoping review. | 1, 36 |

JBI = Joanna Briggs Institute; PRISMA-ScR = Preferred Reporting Items for Systematic reviews and Meta-Analyses extension for Scoping Reviews.

* Where *sources of evidence* (see second footnote) are compiled from, such as bibliographic databases, social media platforms, and Web sites.

† A more inclusive/heterogeneous term used to account for the different types of evidence or data sources (e.g., quantitative and/or qualitative research, expert opinion, and policy documents) that may be eligible in a scoping review as opposed to only studies. This is not to be confused with *information sources* (see first footnote).

‡ The frameworks by Arksey and O’Malley (6) and Levac and colleagues (7) and the JBI guidance (4, 5) refer to the process of data extraction in a scoping review as data charting*.*

§ The process of systematically examining research evidence to assess its validity, results, and relevance before using it to inform a decision. This term is used for items 12 and 19 instead of "risk of bias" (which is more applicable to systematic reviews of interventions) to include and acknowledge the various sources of evidence that may be used in a scoping review (e.g., quantitative and/or qualitative research, expert opinion, and policy document).

*From:* Tricco AC, Lillie E, Zarin W, O'Brien KK, Colquhoun H, Levac D, et al. PRISMA Extension for Scoping Reviews (PRISMAScR): Checklist and Explanation. Ann Intern Med. 2018;169:467–473. [doi: 10.7326/M18-0850](http://annals.org/aim/fullarticle/2700389/prisma-extension-scoping-reviews-prisma-scr-checklist-explanation).

# **A.2. Bibliometric index metrics**

- **h index**

The h index attempts to measure both the productivity and citation impact of the published body of work of an entity (e.g., author, institution, journal) [1,2]. It refers to the total number of publications by a particular entity with at least the same number of citations.

- **m index**

The m index is calculated by dividing the h index by the number of years of an entity’s productive life (e.g., researcher) [1].

- **g index**

The g index of an entity corresponds to the largest number g such that the top g publications have at least g^2^ or more citations together [2].

- **Y index**

The Y index refers to the sum of both the total number of first-authored publications and the total number of corresponding-author publications [3].

- **Dominance factor**

The dominance factor (DF) refers, for a particular researcher, to the proportion of multi-authored publications as specific author’s rank to the total number of multi-authored publications [4].

The dominance factor for being a first/primary author (DF_first_) refers to the proportion of multi-authored publications as a first author to the total number of multi-authored publications. It is calculated as follows:

$${DF}_{first}=\frac{number of multi-authored papers as first author}{total number of multi-authored papers}\times100$$

The dominance factor for being a last/senior author (DF_last_) refers to the proportion of multi-authored publications as a last author to the total number of multi-authored publications. It is calculated as follows:

$${DF}_{last}=\frac{number of multi-authored papers as last author}{total number of multi-authored papers}\times100$$

- **Fractionalized frequency**

The fractional frequency (FF) intends to reflect/measure an author’s contribution. For one publication, it is calculated as follows:

${FF}_{i}=\frac{1}{total number of authors in the {paper}_{i}}$

For a given researcher, the FF corresponds to:

${FF}_{author}=\sum_{i=1}^{n} {FF}_{i}$, where n refers to the total number of publications for which a researcher was an author.

- **Annual growth rate**

The annual growth rate refers to the variable’s change in percentage as a year-over-year statistic [5], and is calculated as follows:

$$Annual Growth Rate=\frac{\left( end value-first value \right)}{first value}\times100$$

# **A.3. The BIBLIO checklist for reporting the bibliometric reviews of the biomedical literature**

| **Section/Topic** | **Item No.** | **Checklist item** | **Reported on**  **page No.** |  |
| --- | --- | --- | --- | --- |
| **Title** |  |  |  |  |
| Identification | 1 | Identify the report as a bibliometric review in the title. | 1 |  |
| Issues/topics | 2 | Indicate the key issues/topics under investigation and coverage of time period. | 1 |  |
| **Abstract** |  |  |  |  |
| Structured summary | 3 | Structured summary including (as applicable): background, methods, results (key findings) and conclusions. | 2 |  |
| **Introduction/ Background** |  |  |  |  |
| Justification/ Rationale/ Explanation | 4 | Present review of existing knowledge and epidemiological information. | 6-8 |  |
| Objectives | 5 | Statement of the objective (s) or question (s). | 8 |  |
| **Methods** |  |  |  |  |
| Search engines (data sources) | 6 | Describe all information sources (such as electronic databases, contact with study authors, trial registers or other grey literature sources). | 8, 9 |  |
| Search strategy | 7 | Keywords and systematization criteria (date of search, language, type of document) for the search. | 8, 9 |  |
| Time period | 8 | The period that the review covers and the justification. | 8, 9 |  |
| Eligibility criteria | 9 | Describe all inclusion and exclusion criteria; languages; study design, type of publication and time period. | 8, 9 |  |
| Data refinement (data selection procedure) | 10 | Remove the irrelevant articles; inspection to eliminate duplicate and unrelated articles (after evaluation of the title, abstract and content). | 8, 9 |  |
| Quality assessment (optional) | 11 | Assessment of papers by three authors and the use of assessing checklists. | NA |  |
| Data synthesis | 12 | Describe the methods used for summarizing, handling, synthesis, tabulations or schematic displays. Describe how the data were analyzed. | 9-11 |  |
| **Results** |  |  |  |  |
| Descriptive findings (statistics) | 13 | - Provide details of the search and selection process in a flow diagram.  - Number of citations retrieved (number of publication, year of publication, type of documents, country of publication, articles with the highest impact, most impactful authors, most impactful articles, authors with the highest production, top journals, top institutions, …) | 11-16, supplementary materials |  |
| Schematic map and trend | 14 | Summarize and/or present the schematic maps and trends using an appropriate software to present citations, journals, authors, top journals, time trends, emerging literature, and any relevant indicators (as applicable) [1-5]. | 16-24, supplementary materials |  |
| Tabulation and summarizing the findings | 15 | General recommendation: Studies under consideration could be summarized and organized by different subtitles and different scenarios. Regardless, results need to be presented in separate tables covering each subtitle. The followings are some options that could help to summarize the findings.  *Option 1:*  - Start the presentation with a historical view [when and who first published on the topic].  - Report on review papers. The result should be listed in a separate table. Also, specify the review type (scoping review, narrative review, systematic review, and meta-analysis).  - Summarize the findings according to the study designs and main study types.  *Option 2:*  - Start the presentation with a historical view [when and who first published on the topic].  - Report on review papers. The result should be listed in a separate table. Also, indicate the review type (scoping review, narrative review, systematic review, and meta-analysis) should be specified.  - Summarize the findings according to outcome measures or populations. For example, see [6].  *Option 3:*  - Start the presentation with a historical view [when and who first published on the topic].  - Report on review papers. The result should be listed in a separate table. Also, specify the review type (scoping review, narrative review, systematic review, and meta-analysis).  - Summarize the findings according to concept [7].  *Option 4.*  - Start the presentation with a historical view [when and who first published on the topic].  - Report on review papers. The result should be listed in a separate table, and also specify the review type (scoping review, narrative review, systematic review, and meta-analysis).  - Summarize the findings according to different subtitles relevant to the main topic [8]. | 11-24, supplementary materials |  |
| Synthesis of findings | 16 | Synthesize the findings as much as possible, find the gap, and propose a model, hypothesis, etc. (if applicable). | 11-24, supplementary materials |  |
| **Discussion** |  |  |  |  |
| Summary of evidence | 17 | Summarize the main findings. The findings should be presented in more "general" or "accessible" terms. | 25-27 |  |
| Interpretation | 18 | Include interpretation consistent with results. Explanations for observed outcomes, similarities, and differences reported would be essential. | 27-34 |  |
| Strengths and limitations | 19 | Discuss the strengths and limitations. | 34-35 |  |
| Conclusion(s) | 20 | Provide a general interpretation of the results with respect to the review questions and objectives, as well as potential implications. | 35 |  |
| 1. McDougal L, Dehingia N, Cheung WW, Dixit A, Raj A. COVID-19 burden, author affiliation and women's well-being: A bibliometric analysis of COVID-19 related publications including focus on low-and middle-income countries. eClinicalMedicine 2022; 52: 101606.  2. Henstock L, Wong R, Tsuchiya A, Spencer A. Behavioral theories that have influenced the way health state preferences are elicited and interpreted: A bibliometric mapping analysis of the ttime trade-off method with VOSviewer visualization. Front Health Serv 2022; 2: 848087.  3. Bodea F, Bungau SG, Negru AP, Radu A, Tarce AG, Tit DM, et al. Exploring new therapeutic avenues for ophthalmic disorders: Glaucoma-related molecular docking evaluation and bibliometric analysis for improved management of ocular diseases. Bioengineering 2023; 10(8): 983.  4. Sang XZ, Wang CQ, Chen W, Rong H, Hou LJ. An exhaustive analysis of post-traumatic brain injury dementia using bibliometric methodologies. Front Neurol 2023; 14: 1165059.  5. Ramli MI, Hamzaid NA, Engkasan JP, Usman J. Respiratory muscle training: a bibliometric analysis of 60 years’ multidisciplinary journey. Biomed Eng Online 2023; 22(1): 50.  6. Akosman I, Kumar N, Mortenson R, Lans A, De La Garza Ramos R, Eleswarapu A,et al. Racial differences in perioperative complications, readmissions, and mortalities after elective spine surgery in the United States: A systematic review using AI-assisted bibliometric analysis. Glob Spine J 2023: 21925682231186759.  7. Tavousi M, Mohammadi S, Sadighi J, Zarei F, Kermani RM, Rostami R, Montazeri A. Measuring health literacy: A systematic review and bibliometric analysis of instruments from 1993 to 2021. Plos One 2022; 17(7): e0271524.  8. Montazeri A. Health-related quality of life in breast cancer patients: A bibliographic review of the literature from 1974 to 2007. J Exp Clin Cancer Res 2008; 27: 32. | | | | |

**Rights and permissions:** The original source of the checklist is: Montazeri A, Mohammadi S, M.Hesari P, Ghaemi M, Riazi H, Sheikhi‑Mobarakeh Z. Preliminary guideline for reporting bibliometric reviews of the biomedical literature (BIBLIO): a minimum requirements. *Systematic Reviews* 2023; 12: 239. doi.org/10.1186/s13643-023-02410-2 The article is licensed under a Creative Commons Attribution 4.0 International License (<http://creativecommons.org/licenses/by/4.0/>). A changes was made to the original checklist to add in full references to the cited sources.


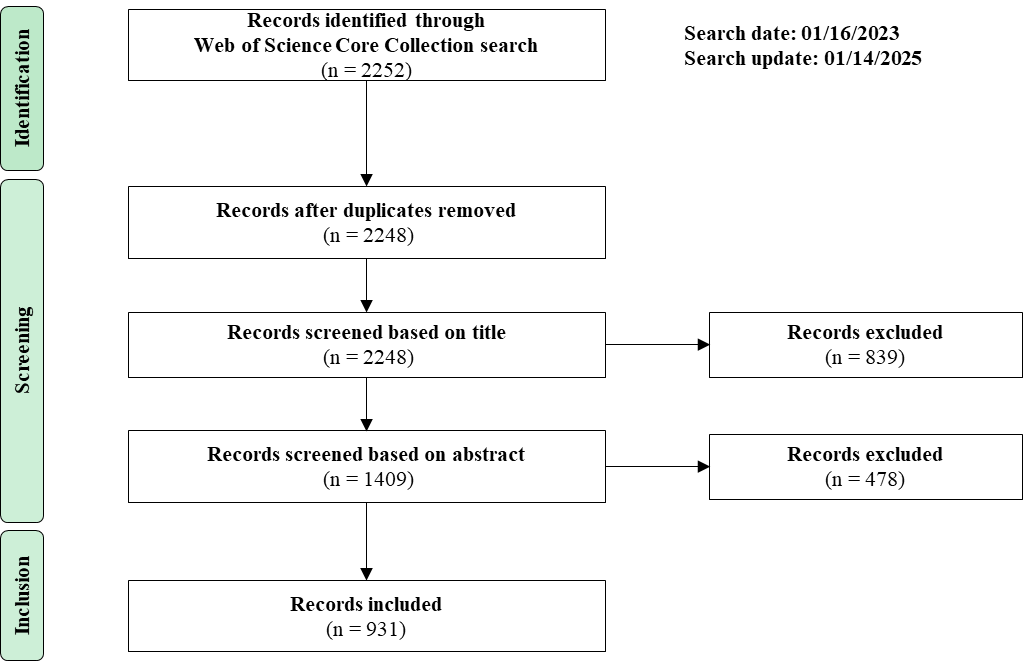


# **Fig.A.1: PRISMA flow chart depicting the literature search and the evaluation process for finding relevant records**


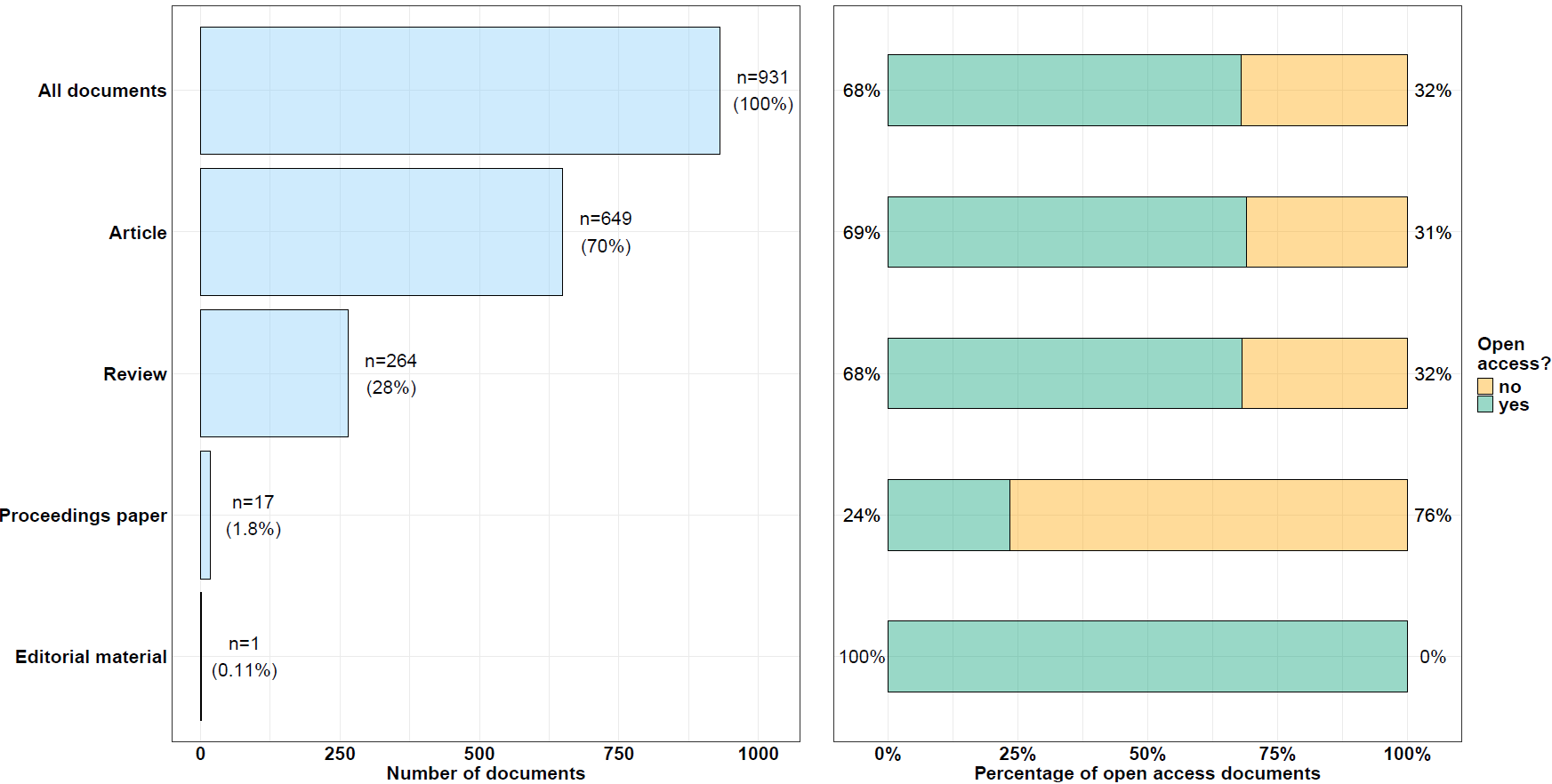


# **Fig.A.2: Number of publications per document type**


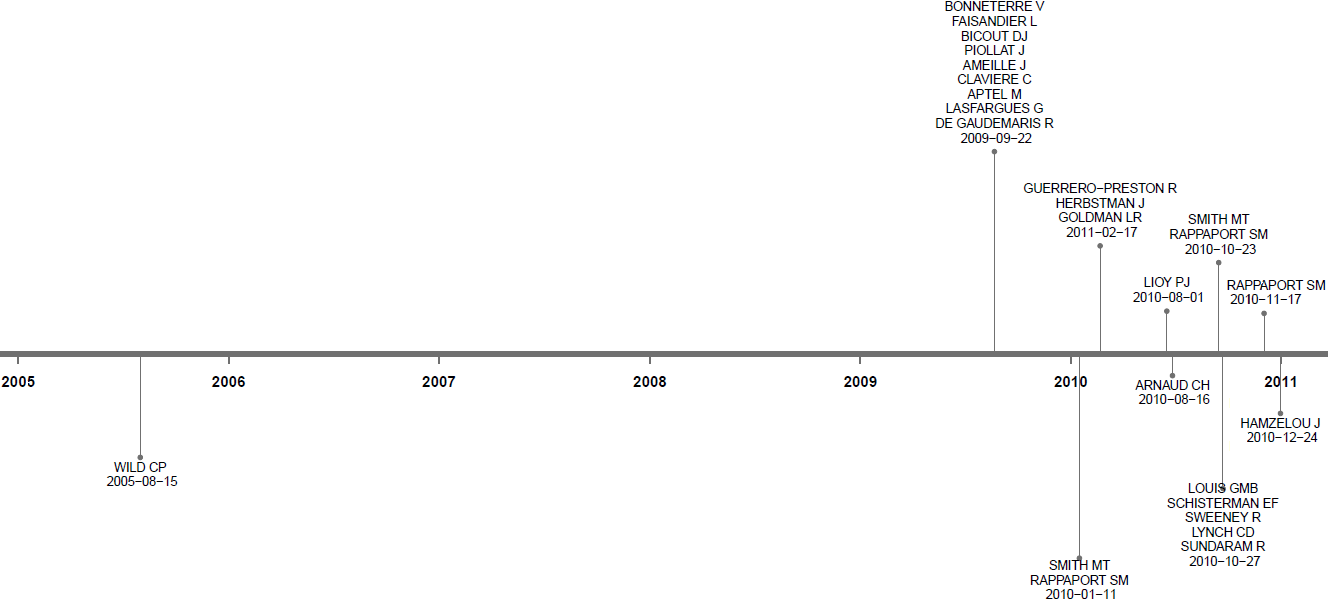


# **Fig.A.3: Timeline of the first 10 publications published on the human exposome**

**
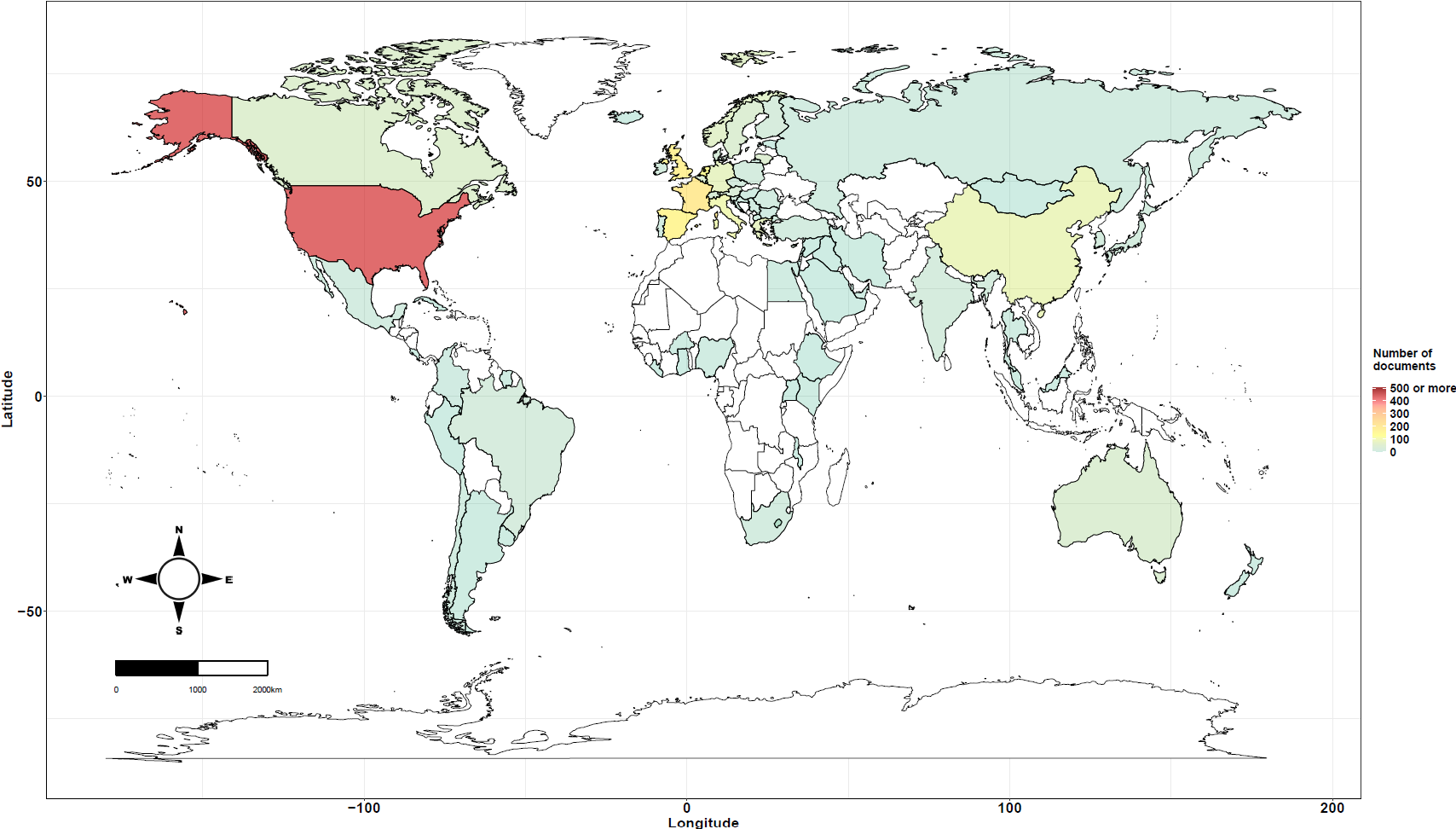
**

# **Fig.A.5: Map of the number of publications per country**

**
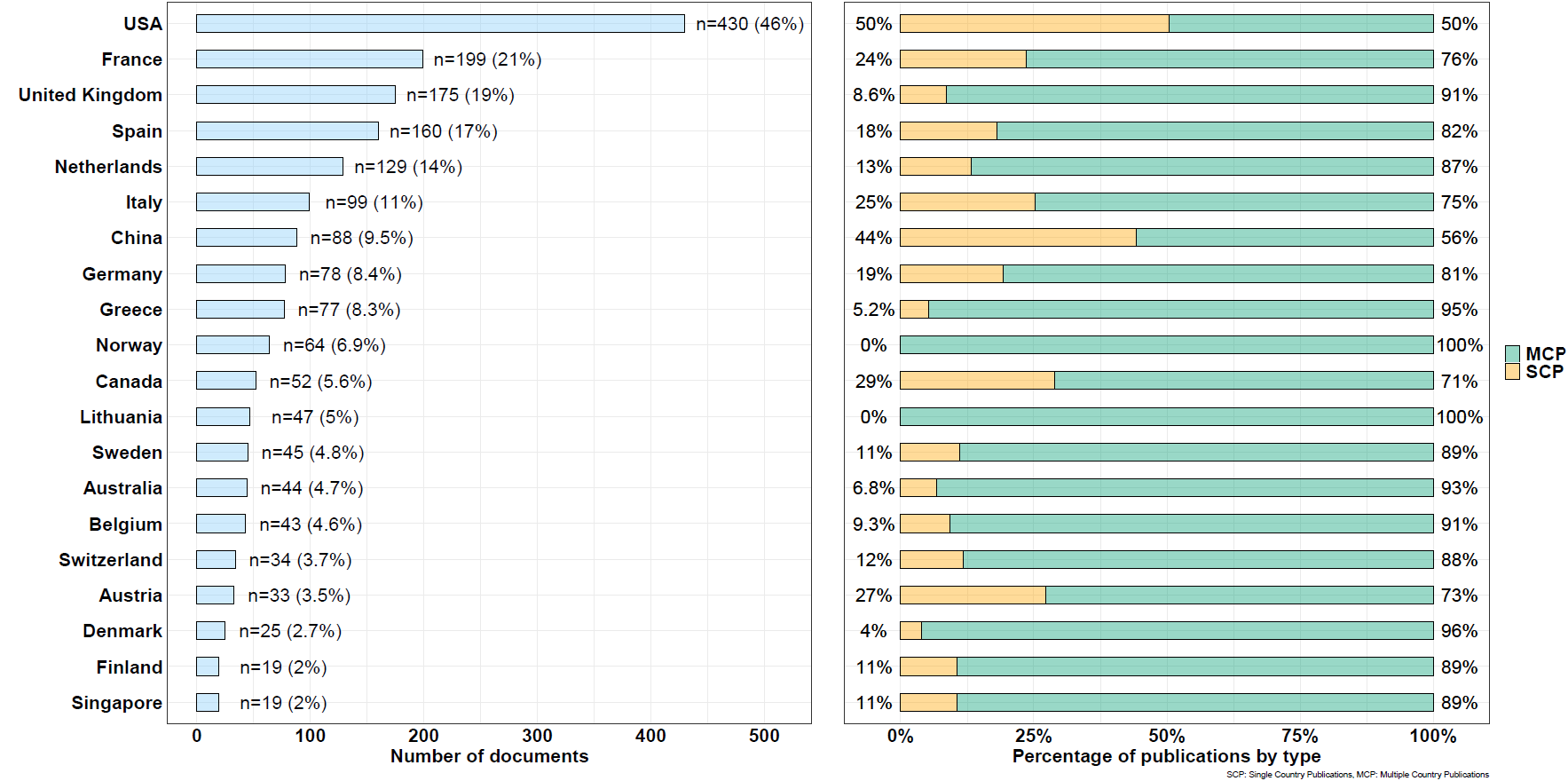
**

# **Fig.A.6: The most productive countries**

**
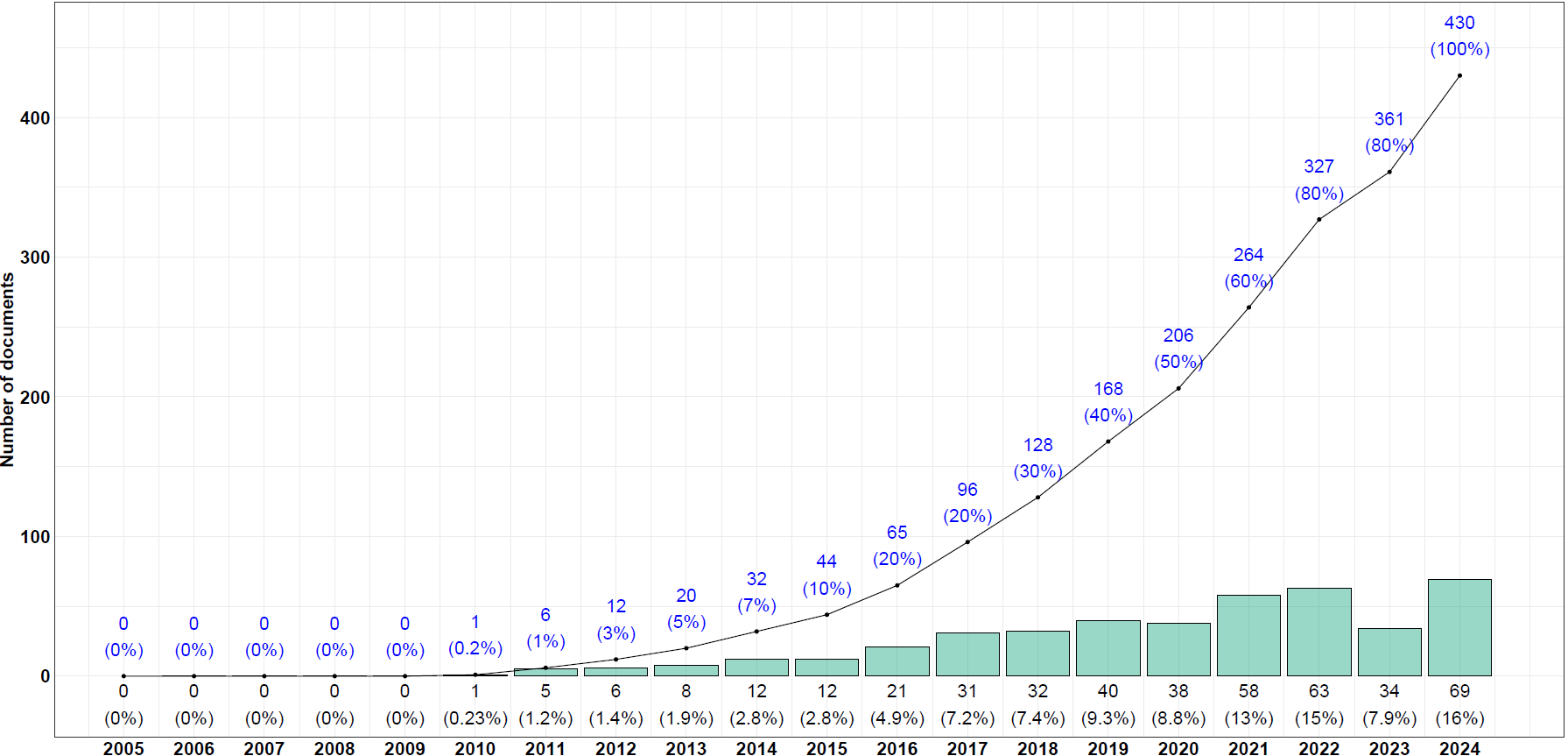
**

# **Fig.A.7: Scientific productivity – number of publications per year for the US**


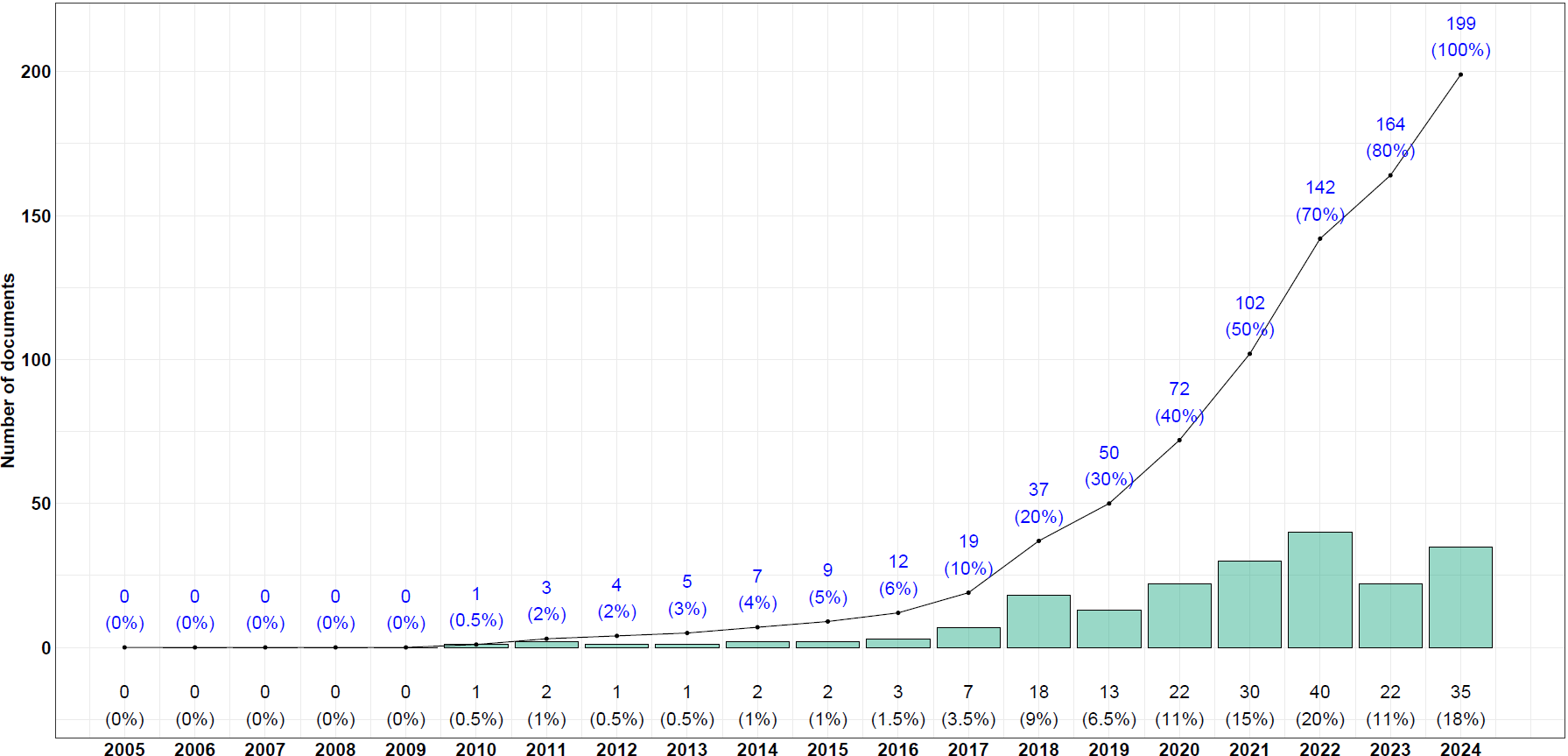


# **Fig.A.8: Scientific productivity – number of publications per year for France**


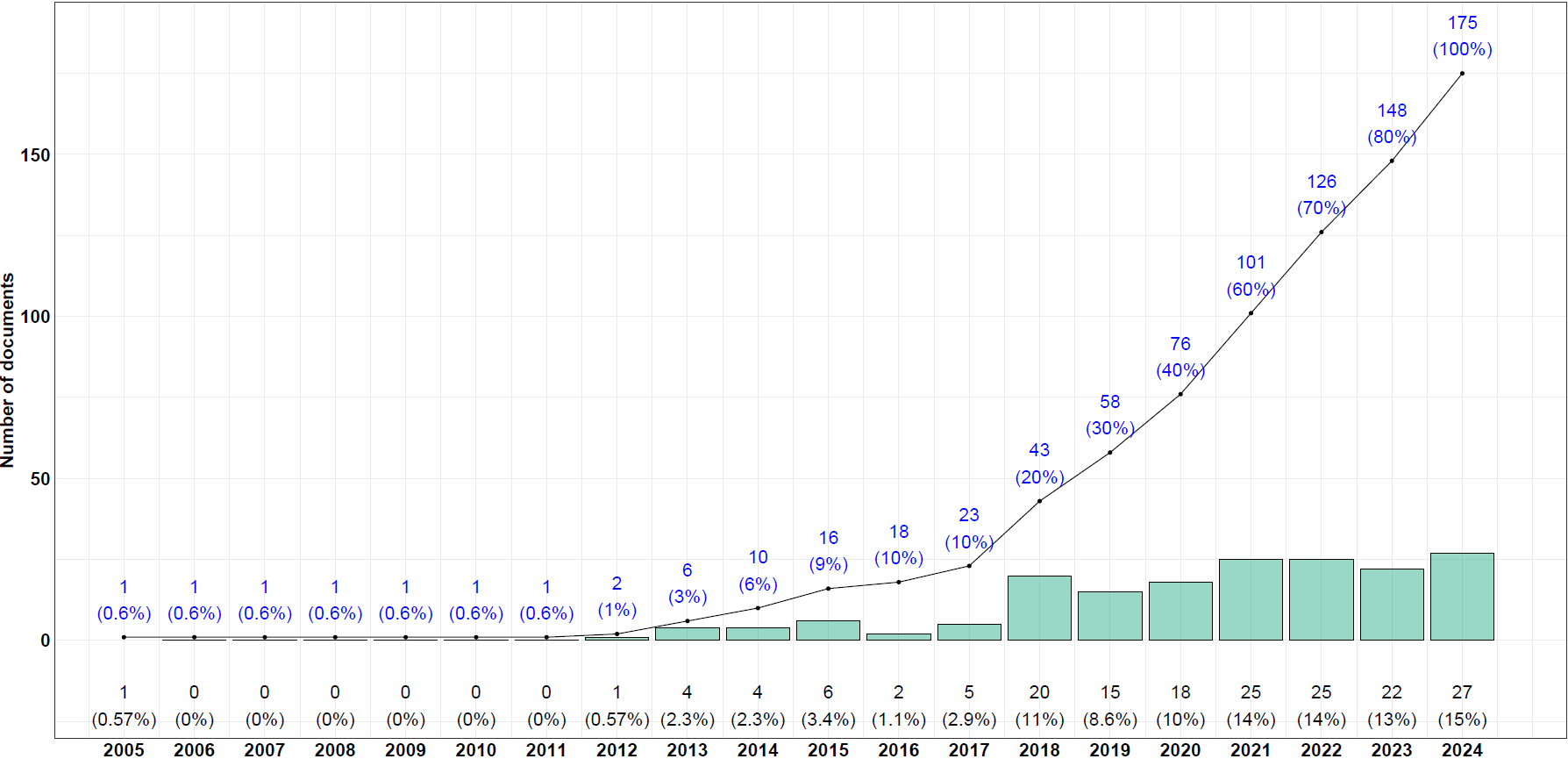


# **Fig.A.9: Scientific productivity – number of publications per year for the UK**


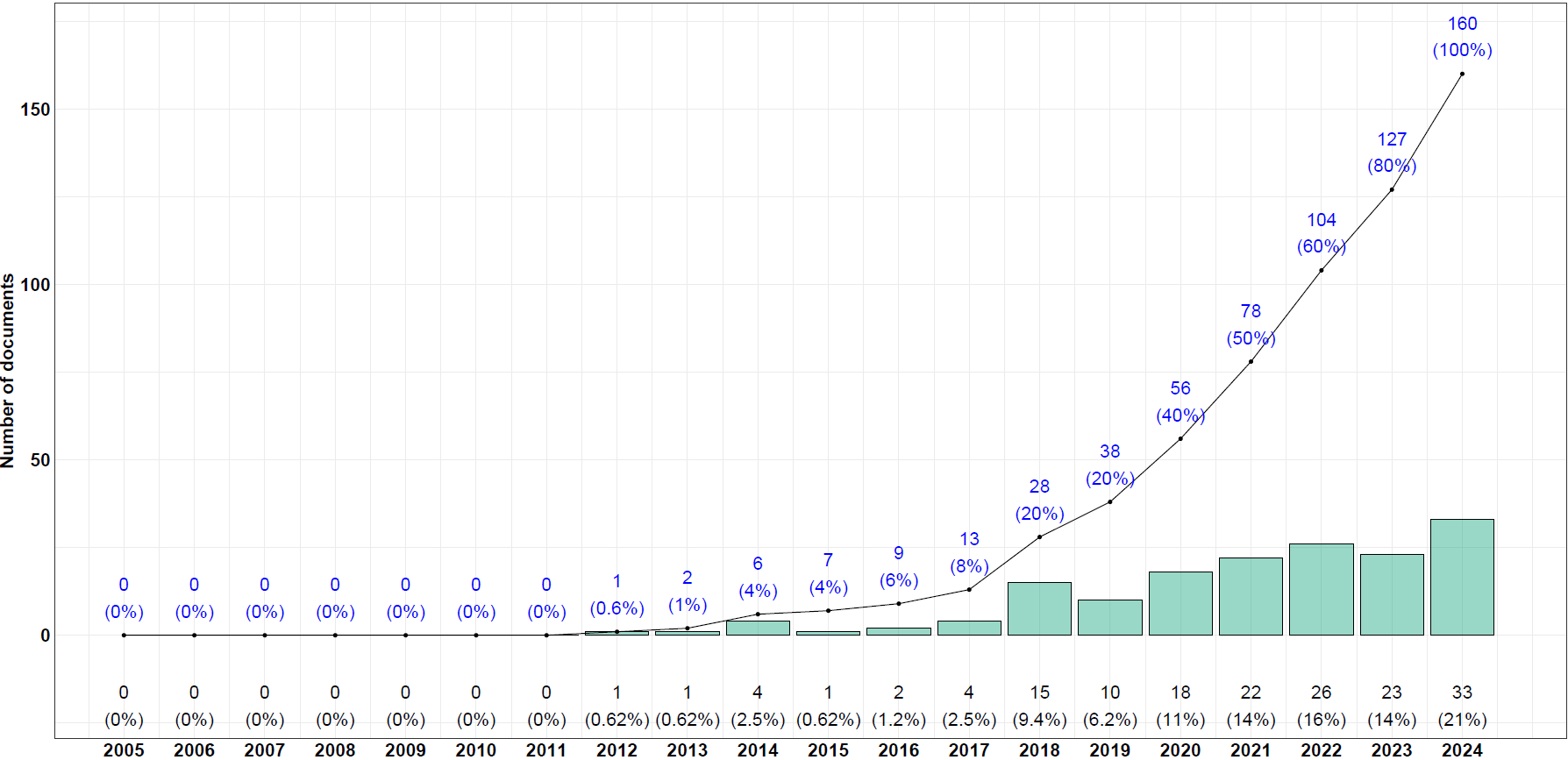


# **Fig.A.10: Scientific productivity – number of publications per year for Spain**


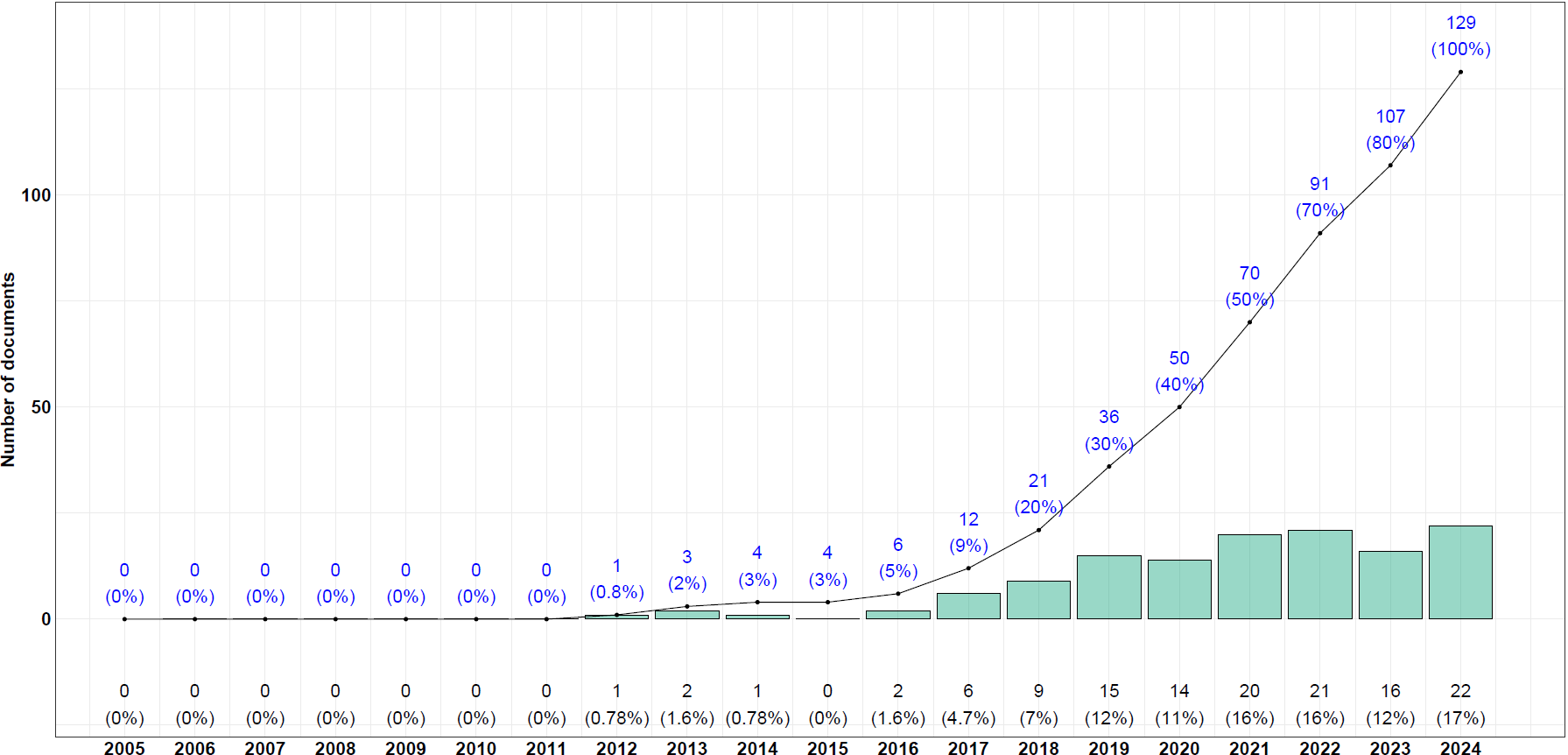


# **Fig.A.11: Scientific productivity – number of publications per year for the Netherlands**


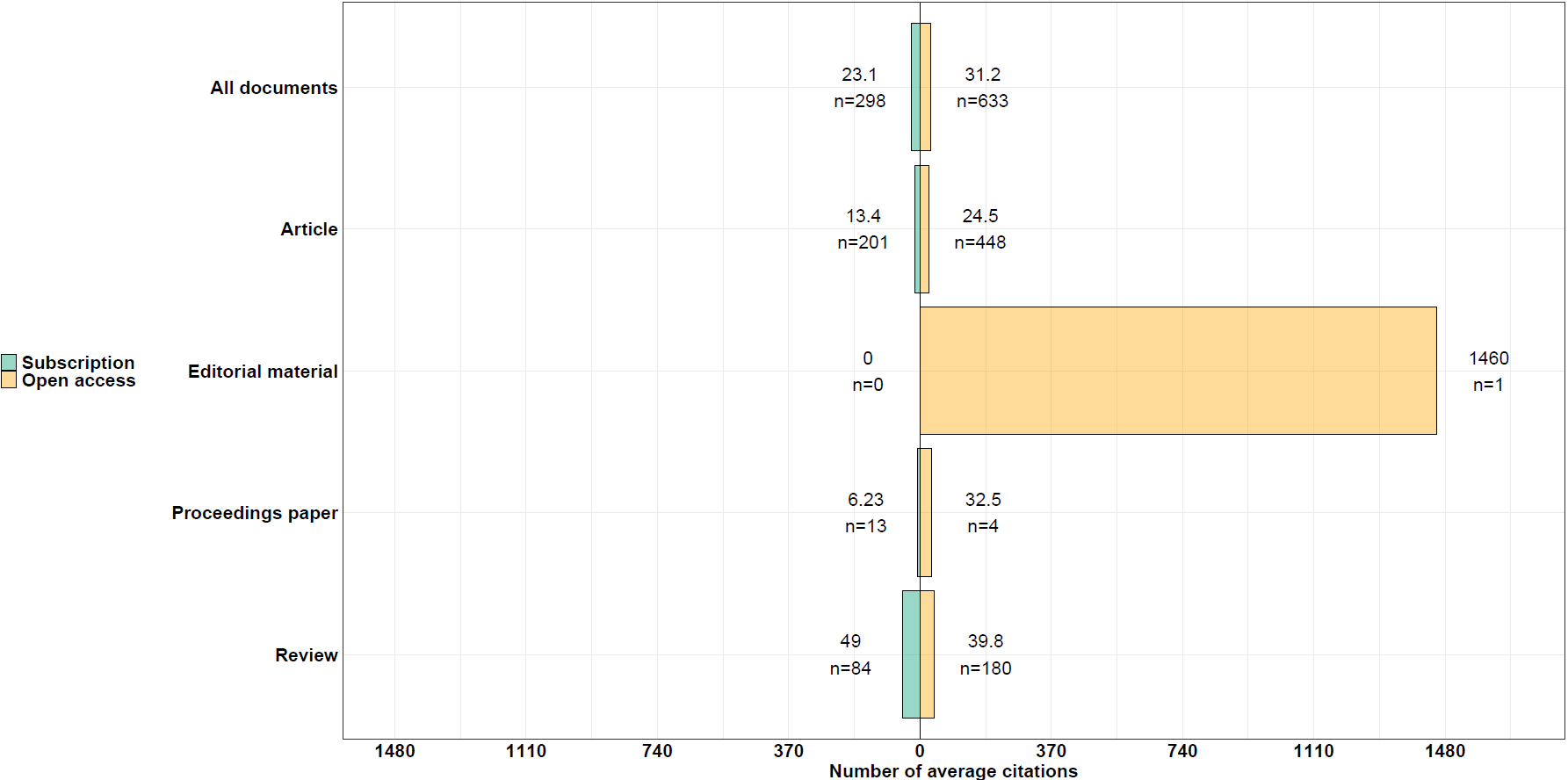


# **Fig.A.12: Number of average citations per document and subscription types**


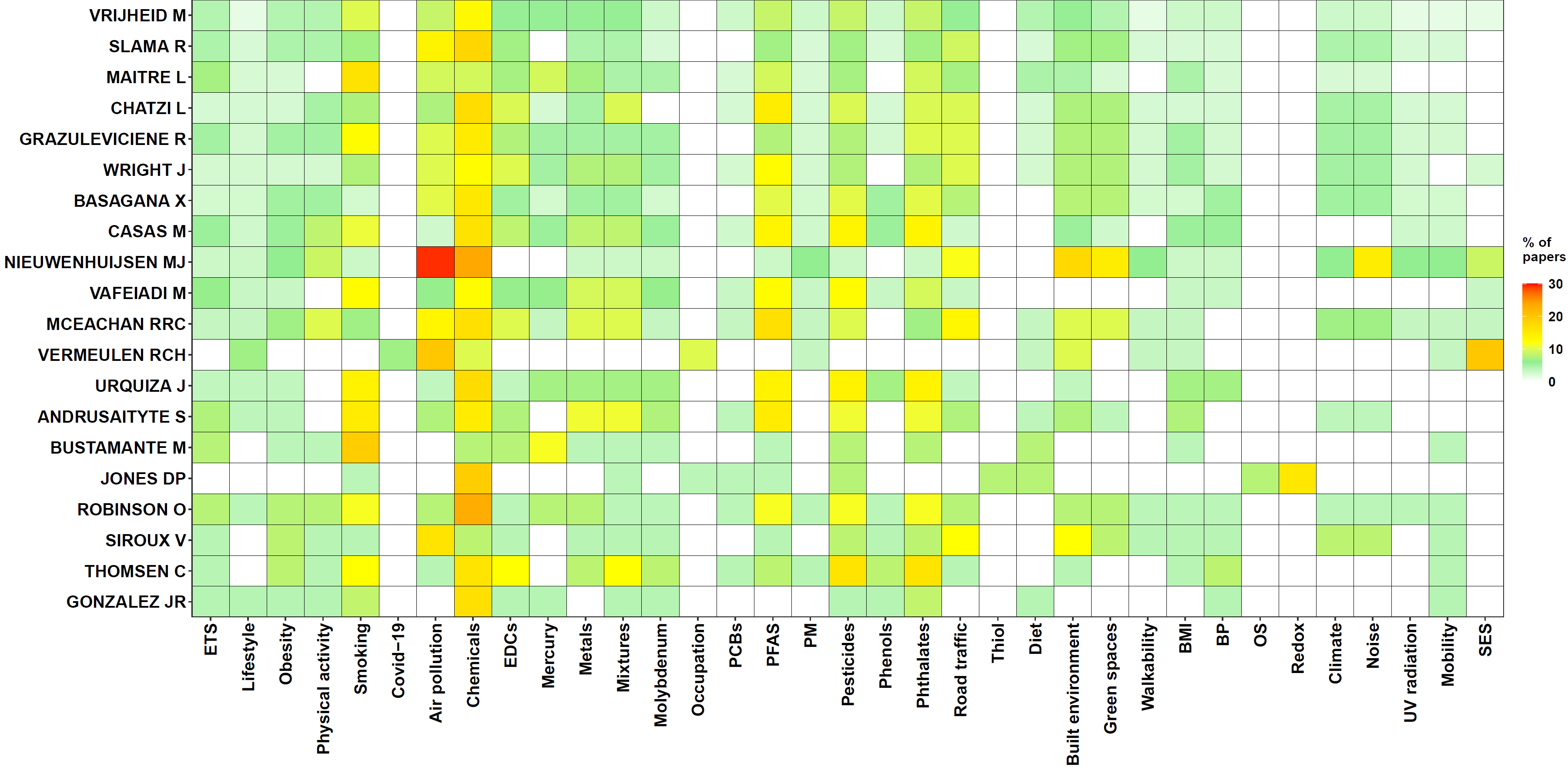


# **Fig.A.13: Most investigated exposures by the top 20 most prolific authors**


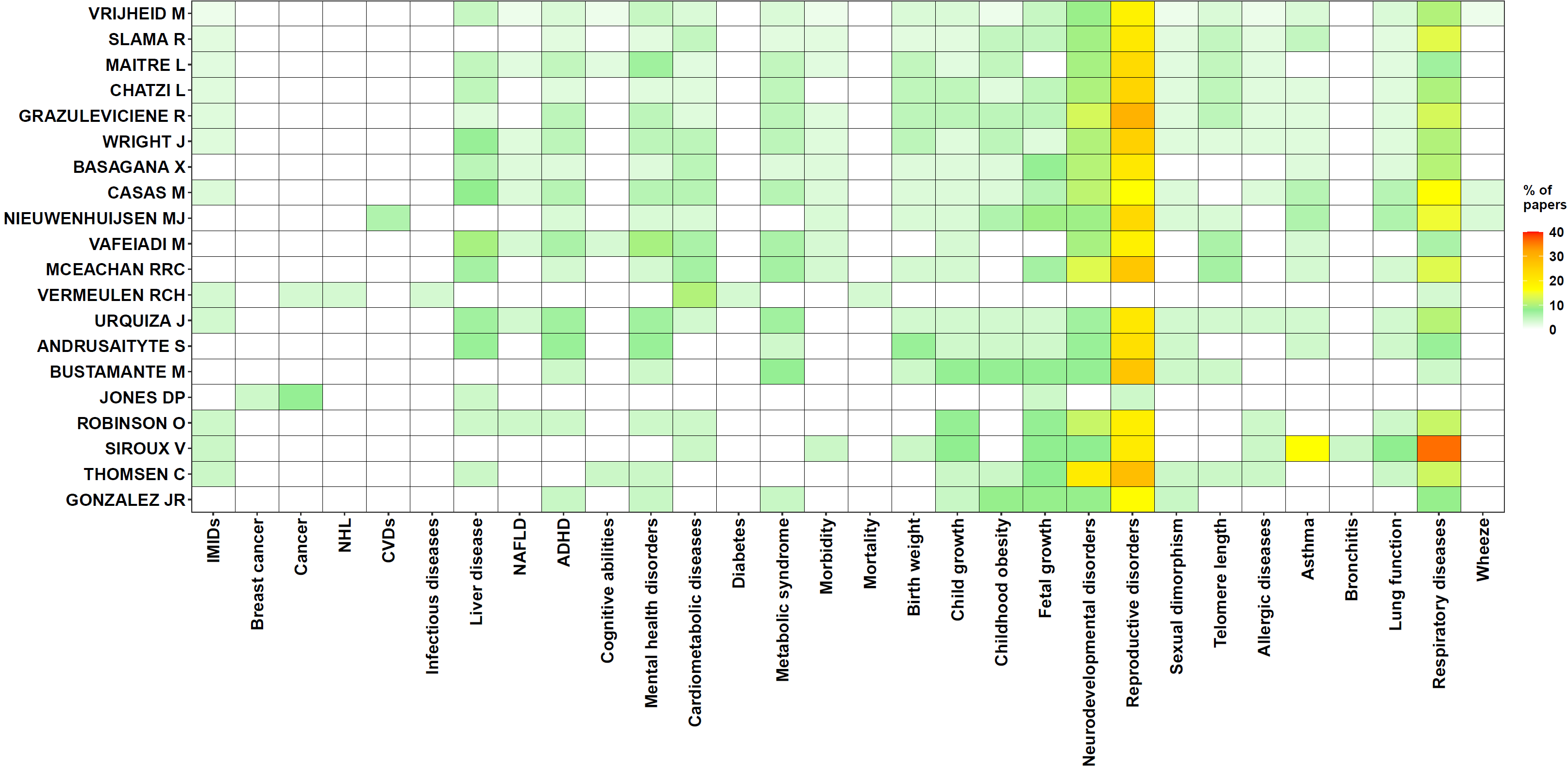


# **Fig.A.14: Most investigated outcomes by the top 20 most prolific authors**


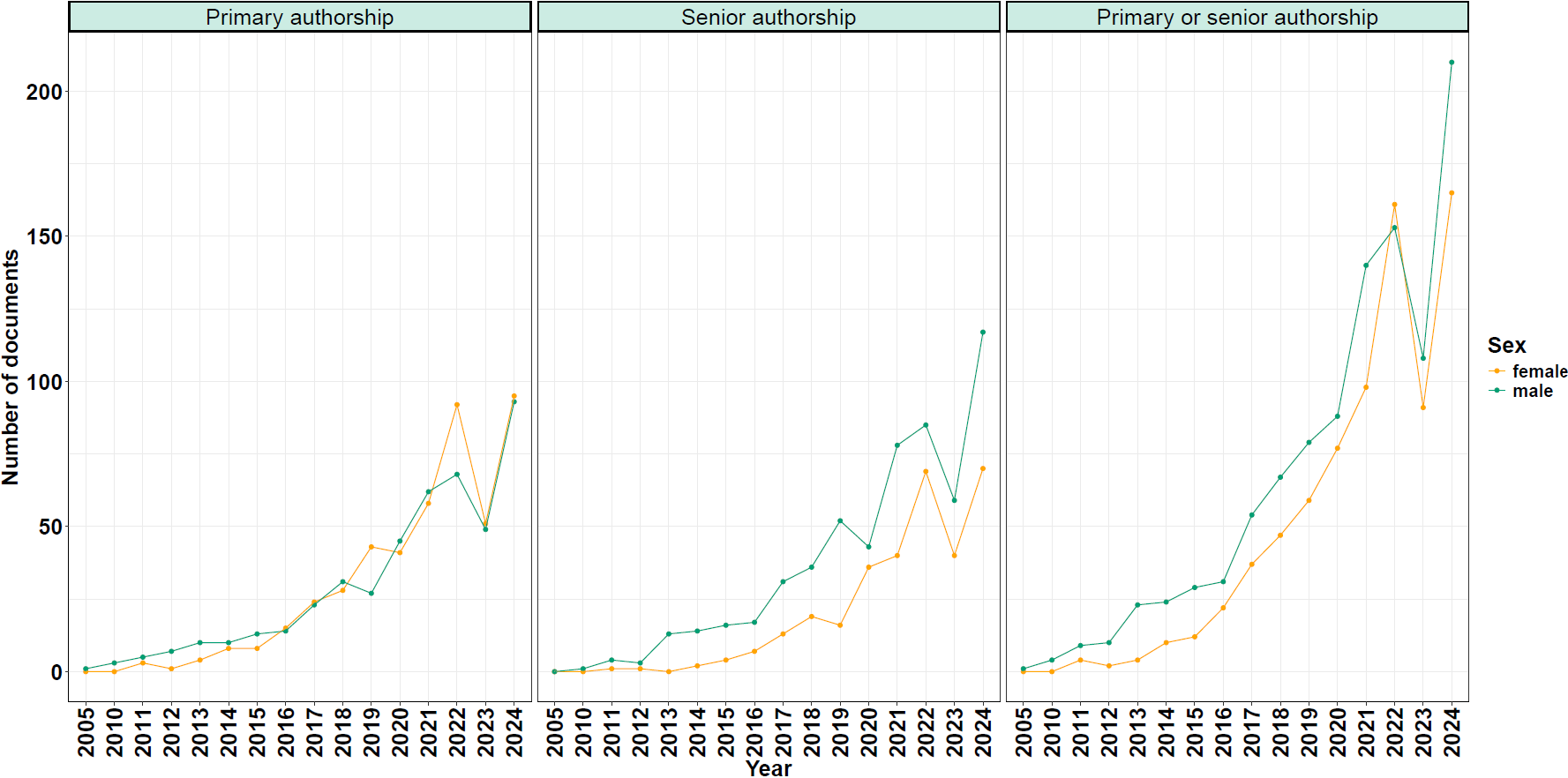


# **Fig.A.15: Total number of publications per year, authorship, and sex**


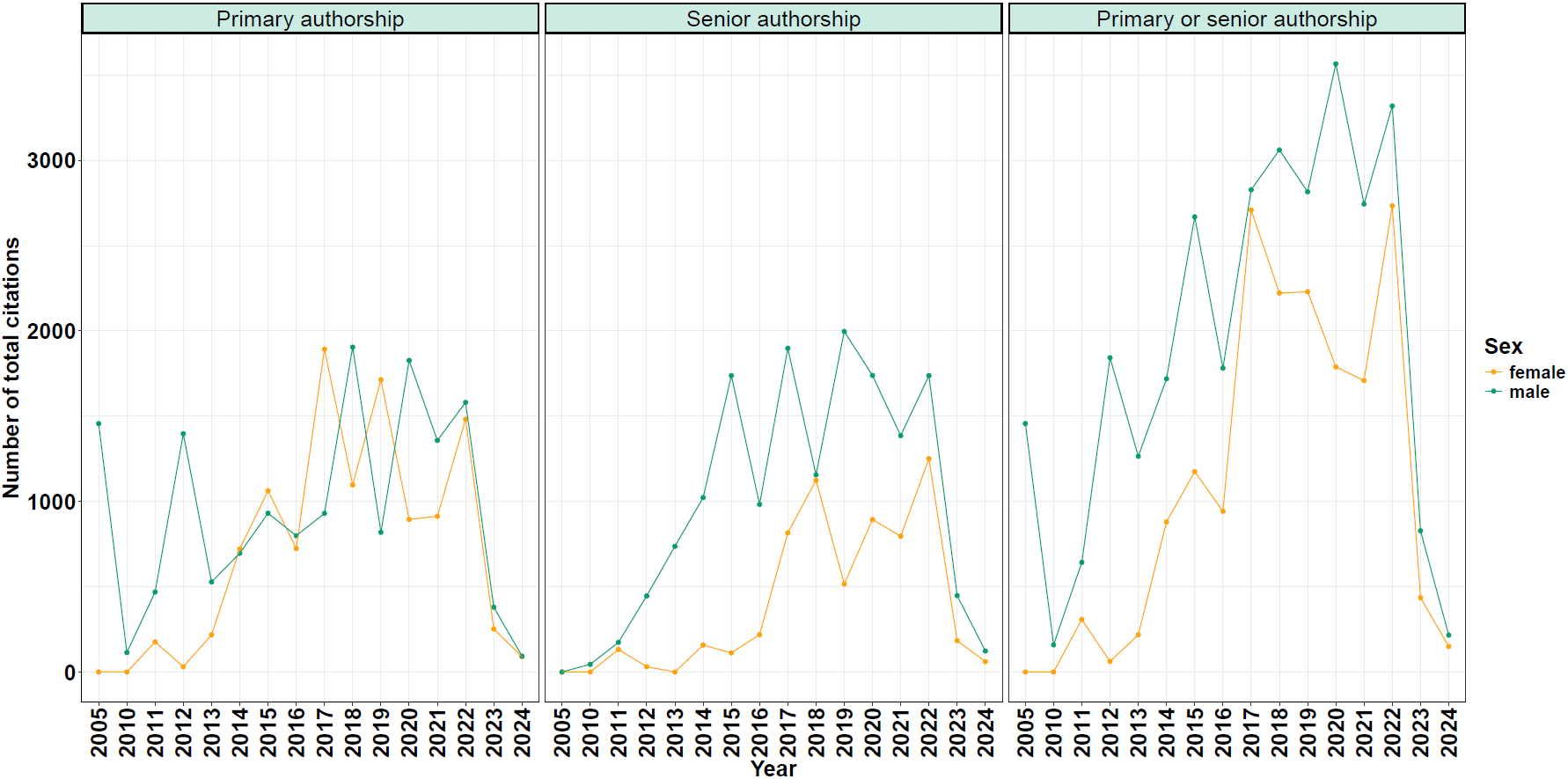


# **Fig.A.16: Total number of citations per year, authorship, and sex**


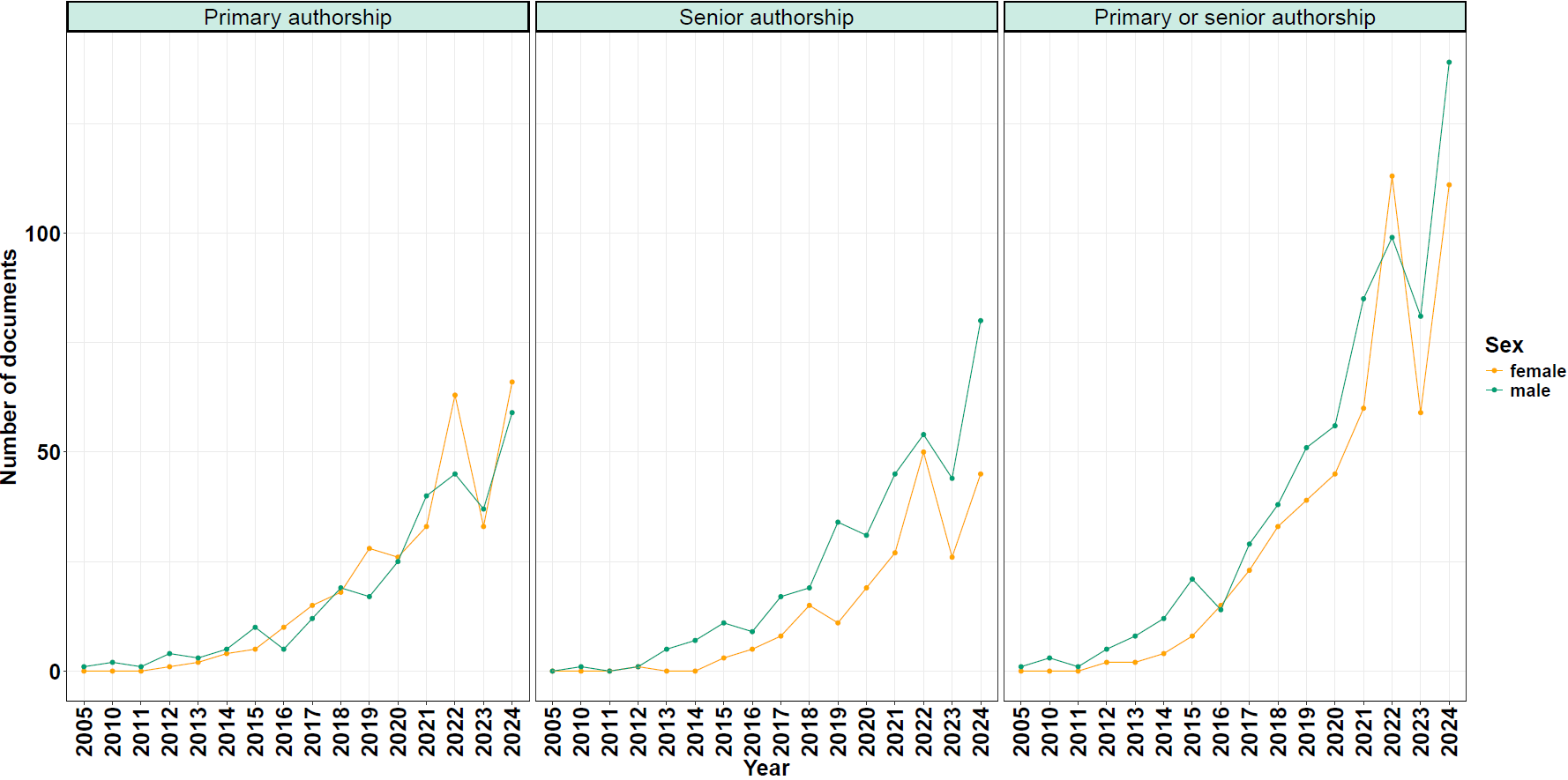


# **Fig.A.17: Total number of publications per year, authorship, and sex (considering only rank 1 journals)**


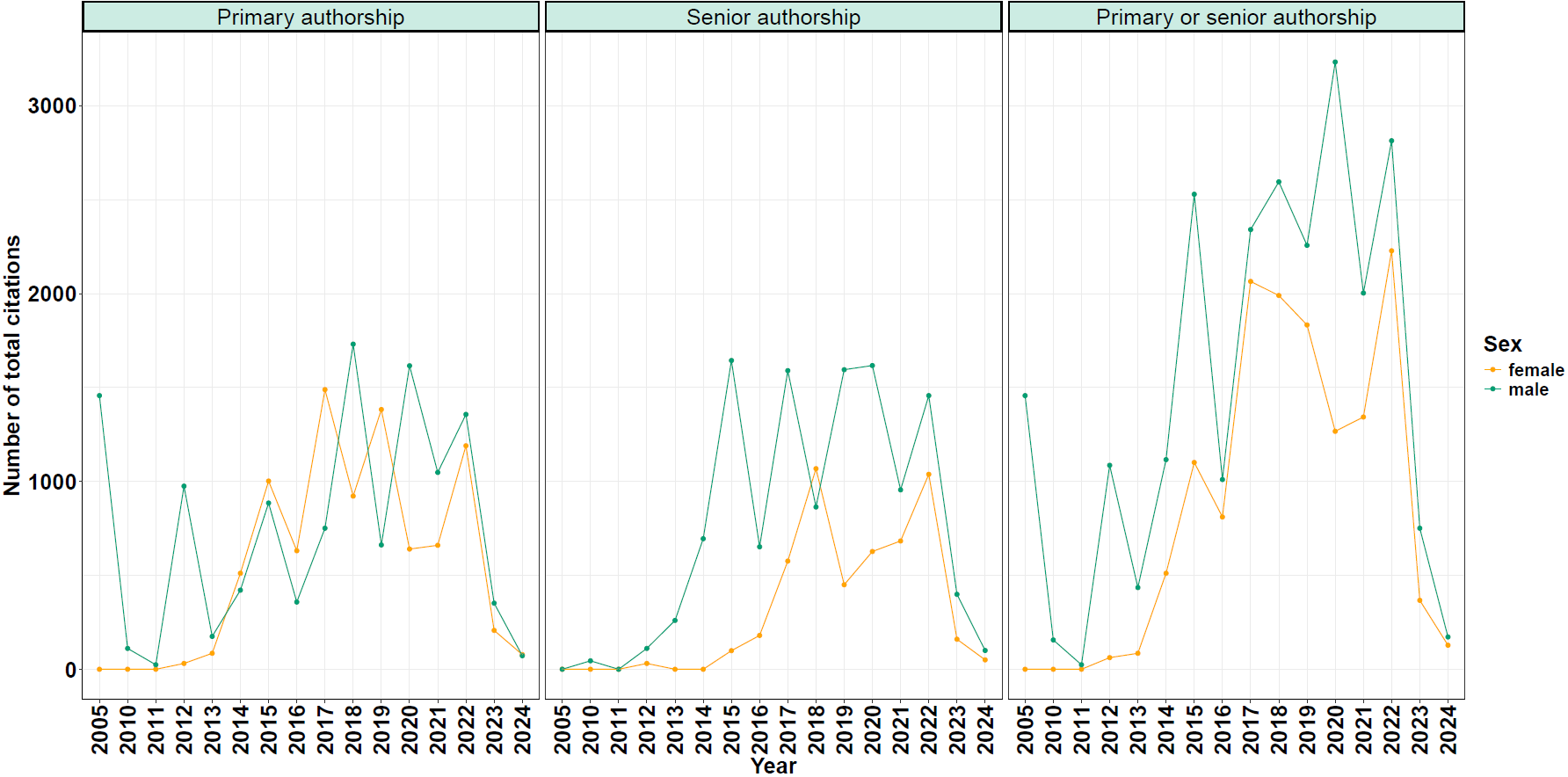


# **Fig.A.18: Total number of citations per year, authorship, and sex (considering only rank 1 journals)**


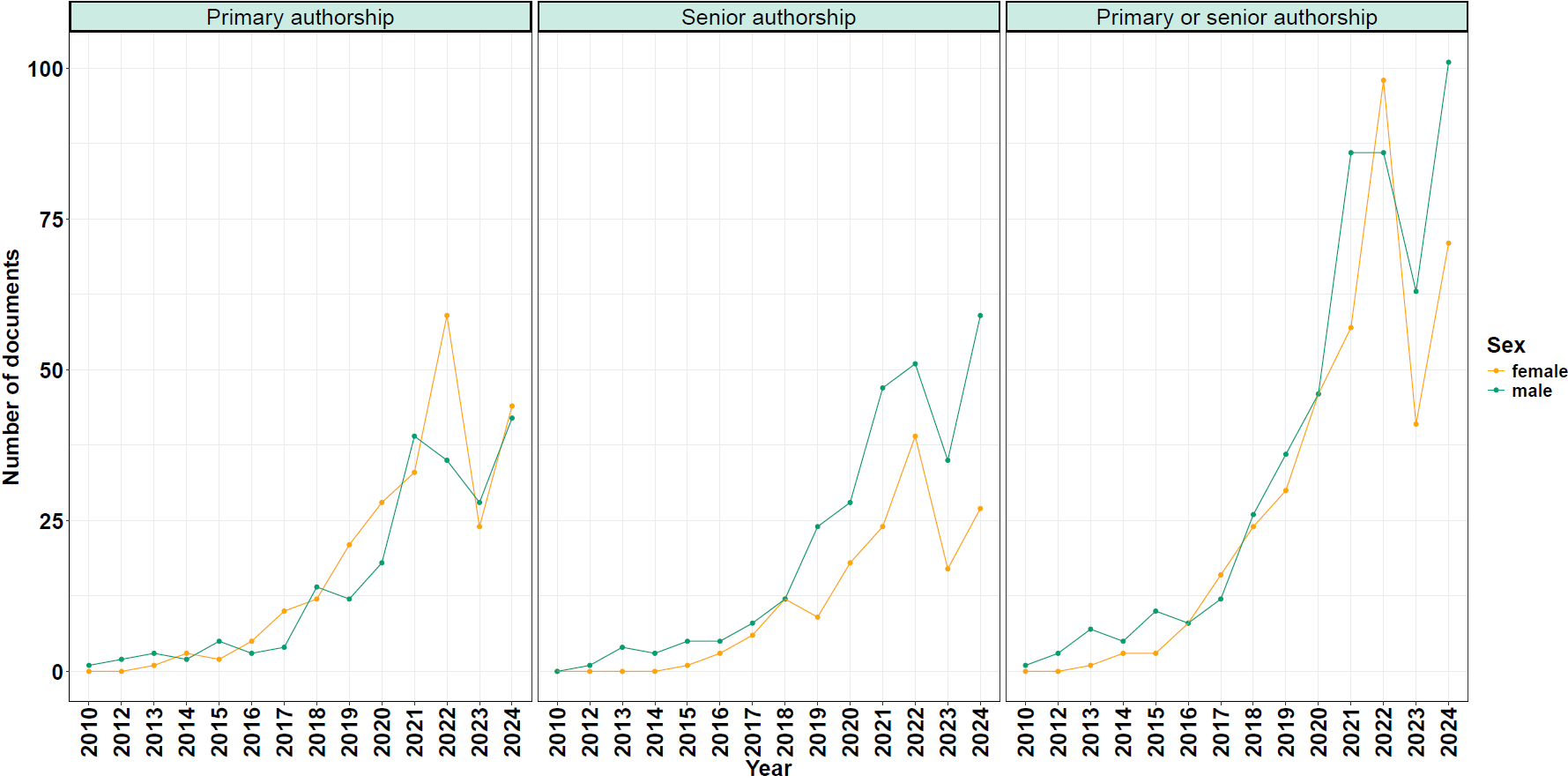


# **Fig.A.19: Total number of publications per year, authorship, and sex (considering only journals with impact factor ≥ 5)**


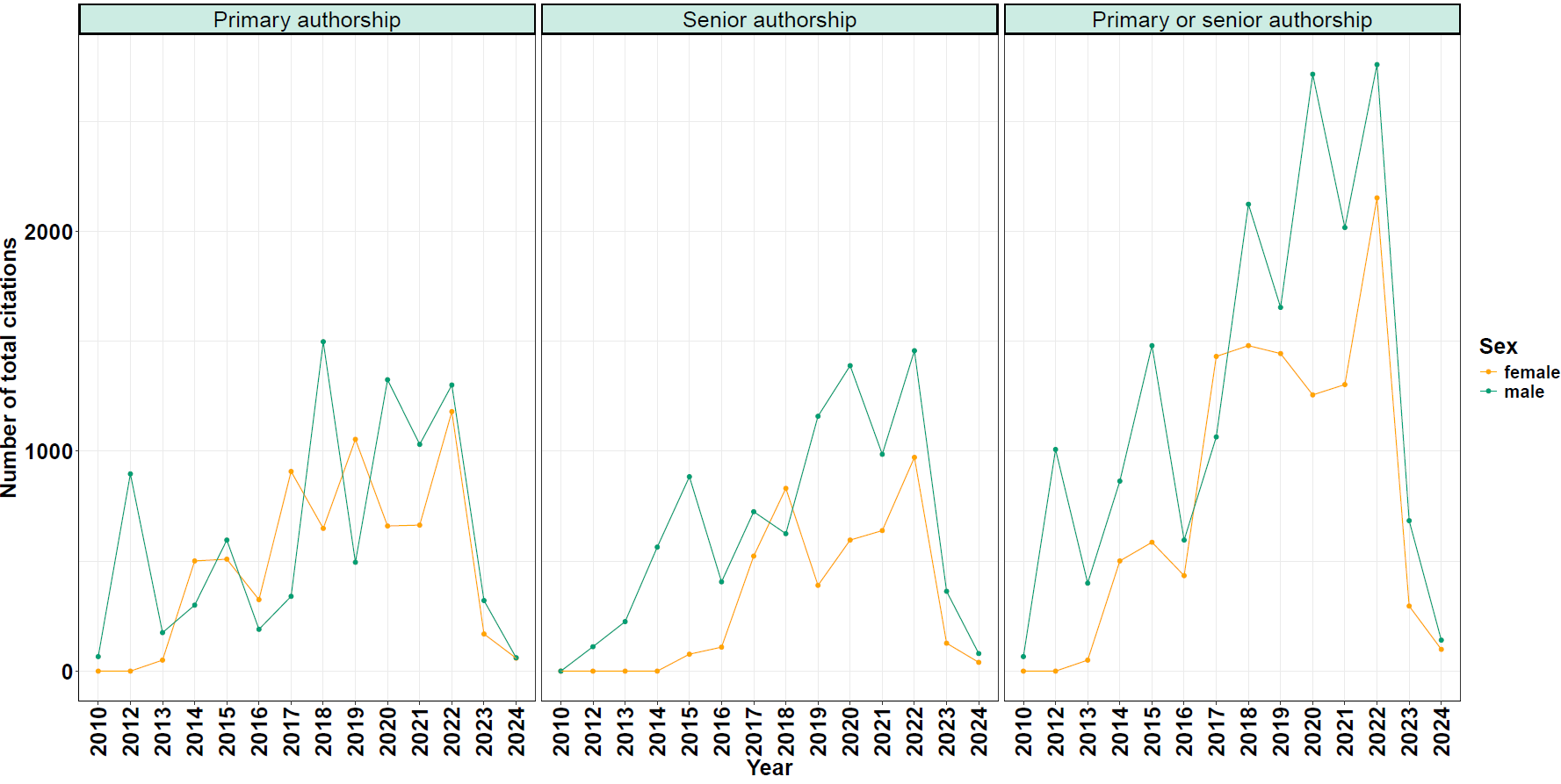


# **Fig.A.20: Total number of citations per year, authorship, and sex (considering only journals with impact factor ≥ 5)**


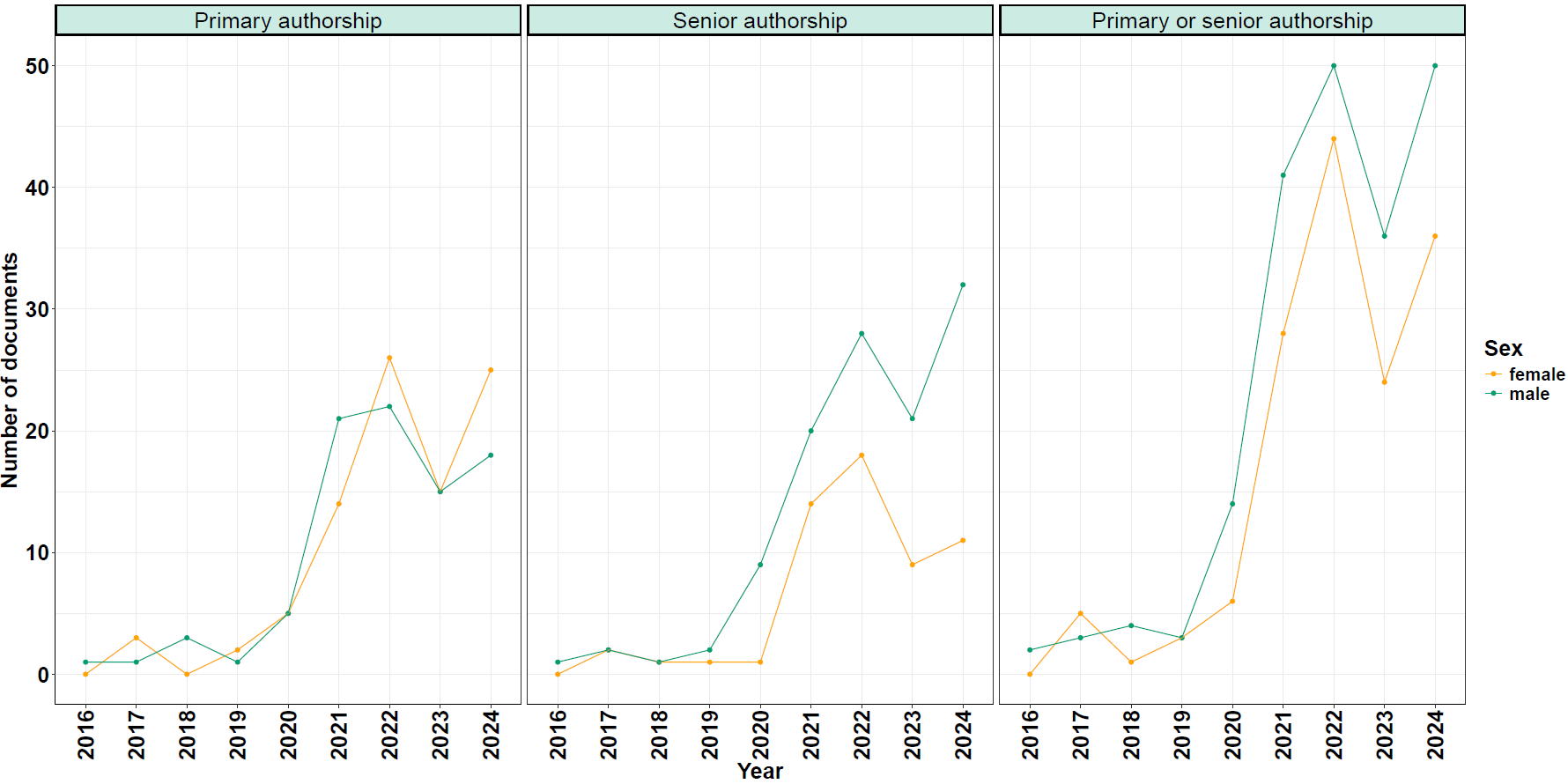


# **Fig.A.21: Total number of publications per year, authorship, and sex (considering only journals with impact factor ≥ 10)**


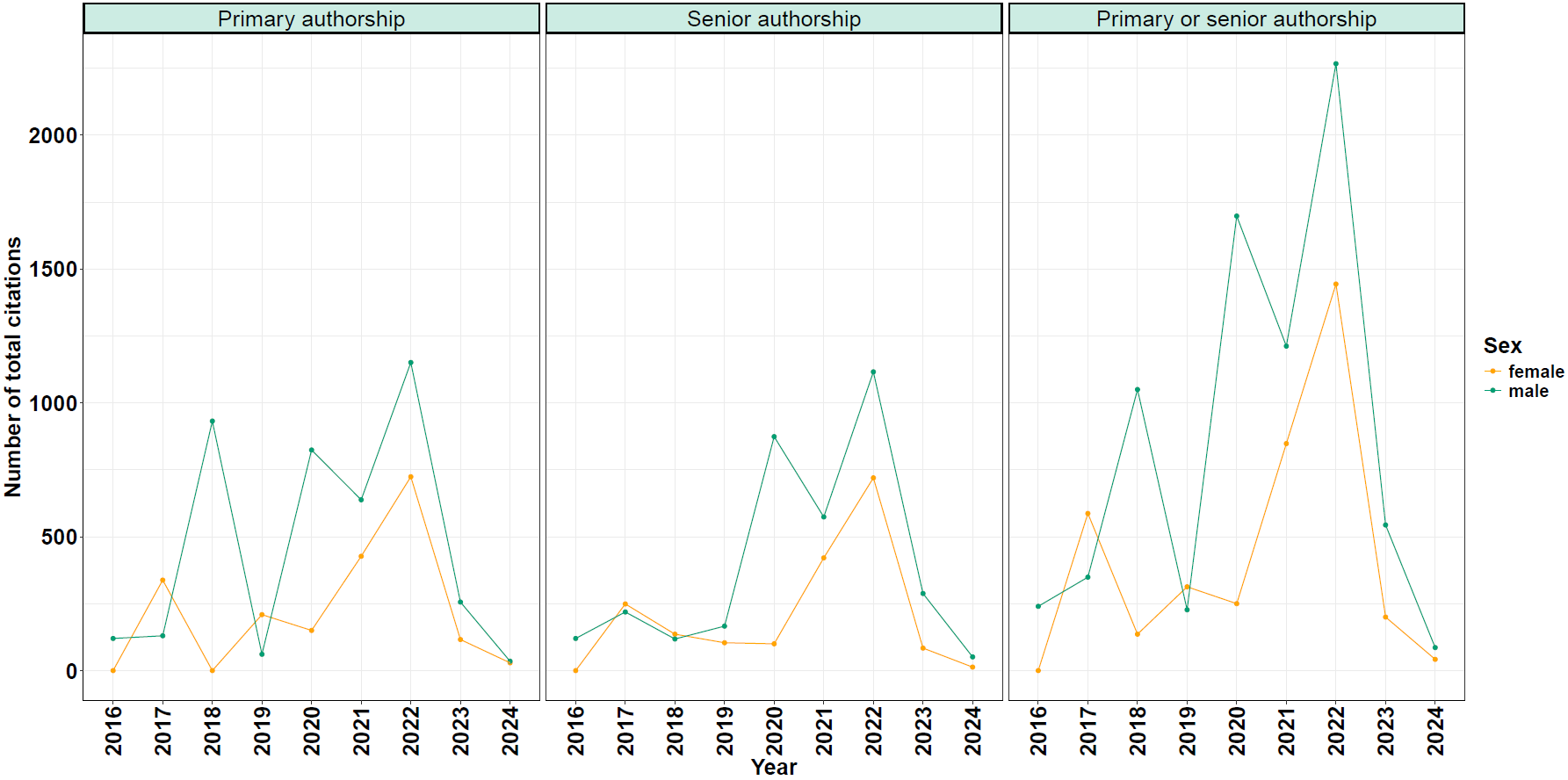


# **Fig.A.22: Total number of citations per year, authorship, and sex (considering only journals with impact factor ≥ 10)**


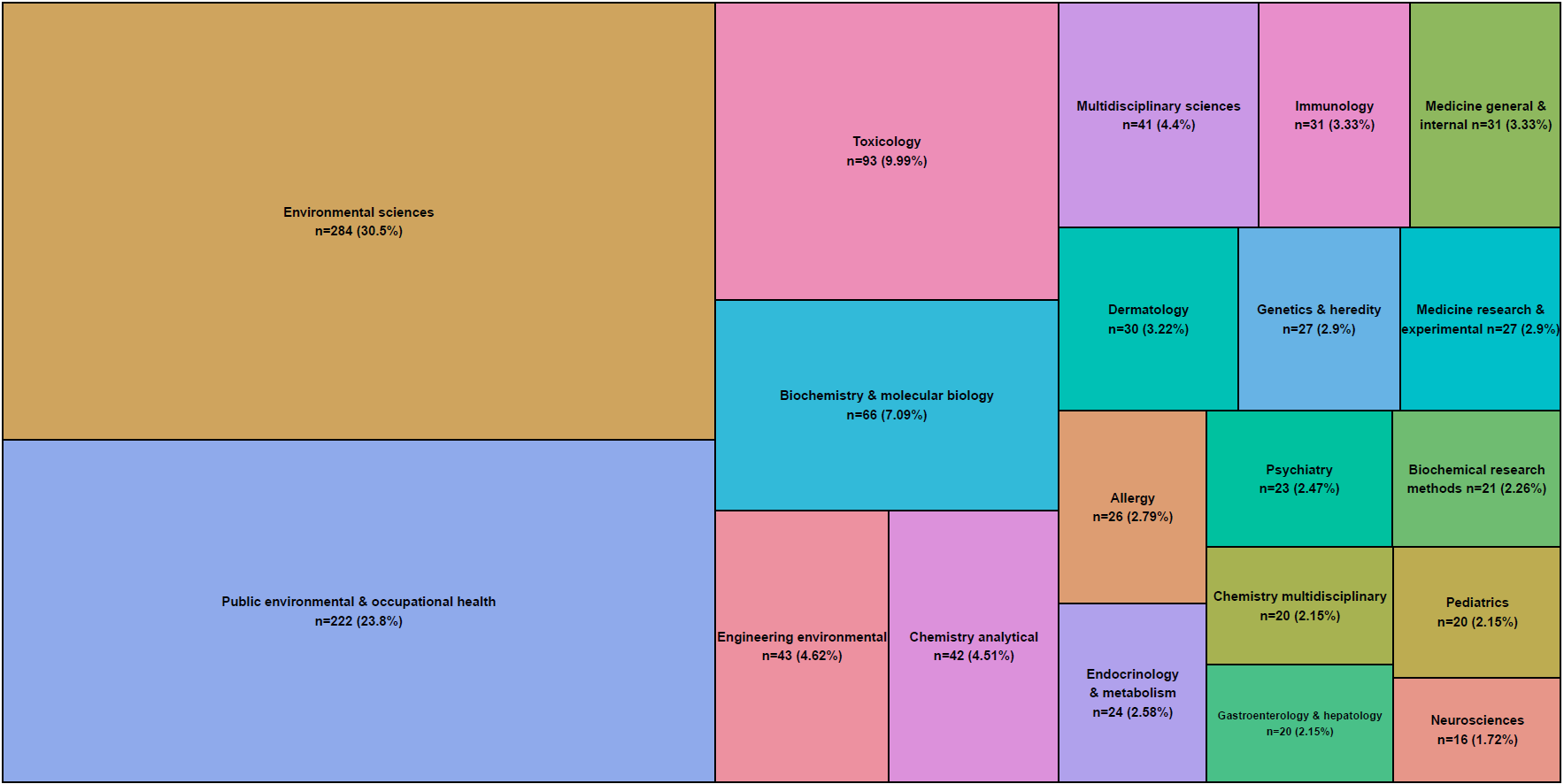


# **Fig.A.23: Tree map of the top 20 research topics/areas**

**
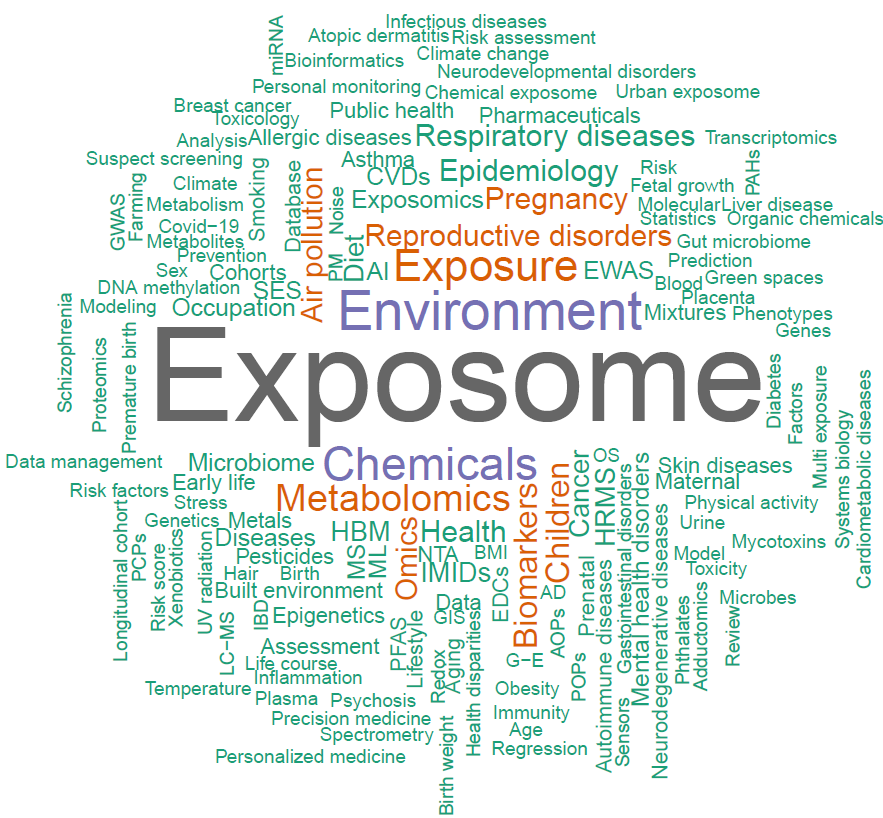
**

# **Fig.A.24: Word cloud of the most frequent keywords**

**
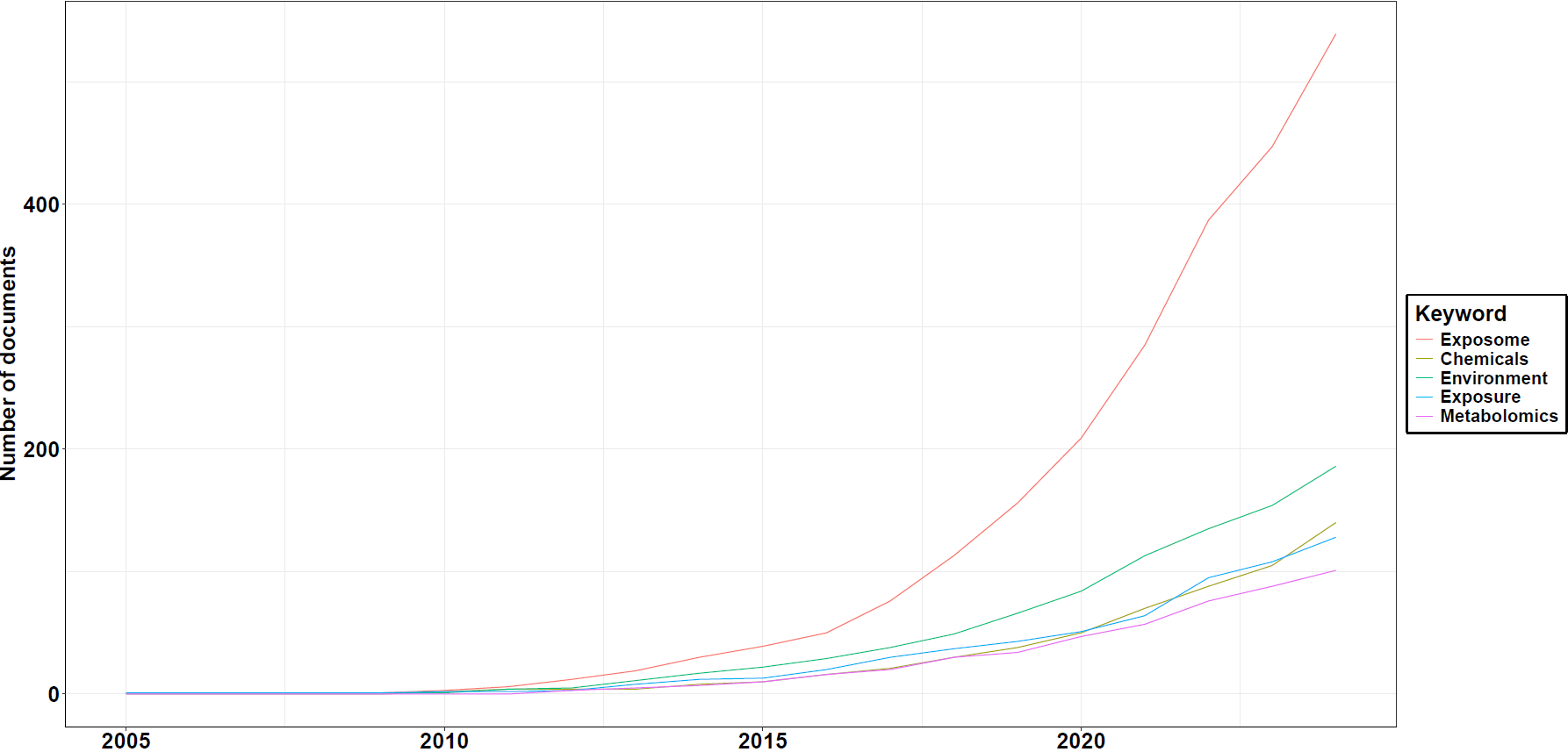
**

# **Fig.A.25: Top 5 of the most frequent keywords – growth trends**

**
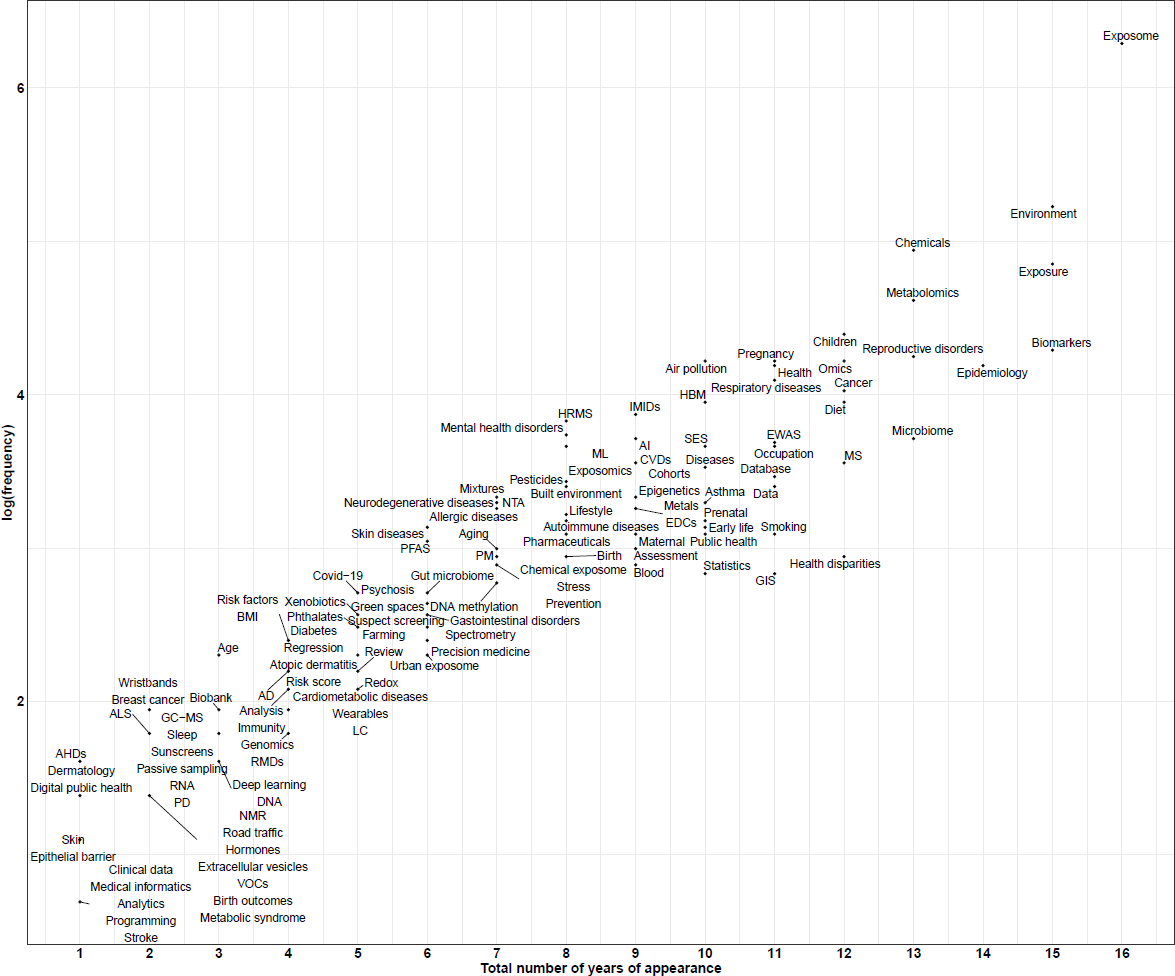
**

# **Fig.A.26:** **Top ten of the most popular keywords by total number of years of appearance**

AD: Alzheimer’s disease, AHDs: administrative health databases, AI: artificial intelligence, ALS: amyotrophic lateral sclerosis, BMI: body mass index, CVDs: cardiovascular diseases, DNA: deoxyribonucleic acid, EDCs: endocrine-disrupting chemicals, EWAS: Exposome-wide association study, GC-MS: gas chromatography – mass spectrometry, GIS: geographic information system, HBM: human biological monitoring, HRMS: high-resolution mass spectrometry, IMIDs: immune-mediated inflammatory diseases, LC: liquid chromatography, ML: machine learning, MS: mass spectrometry, NMR: nuclear magnetic resonance, NTA: non-targeted analysis, PD: Parkinson’s disease, PFAS: per- and polyfluoroalkyl substances, PM: particulate matter, RNA: ribonucleic acid, RMDs: rheumatic and musculoskeletal disorders, SES: socio-economic status, VOCs: volatile organic compounds.


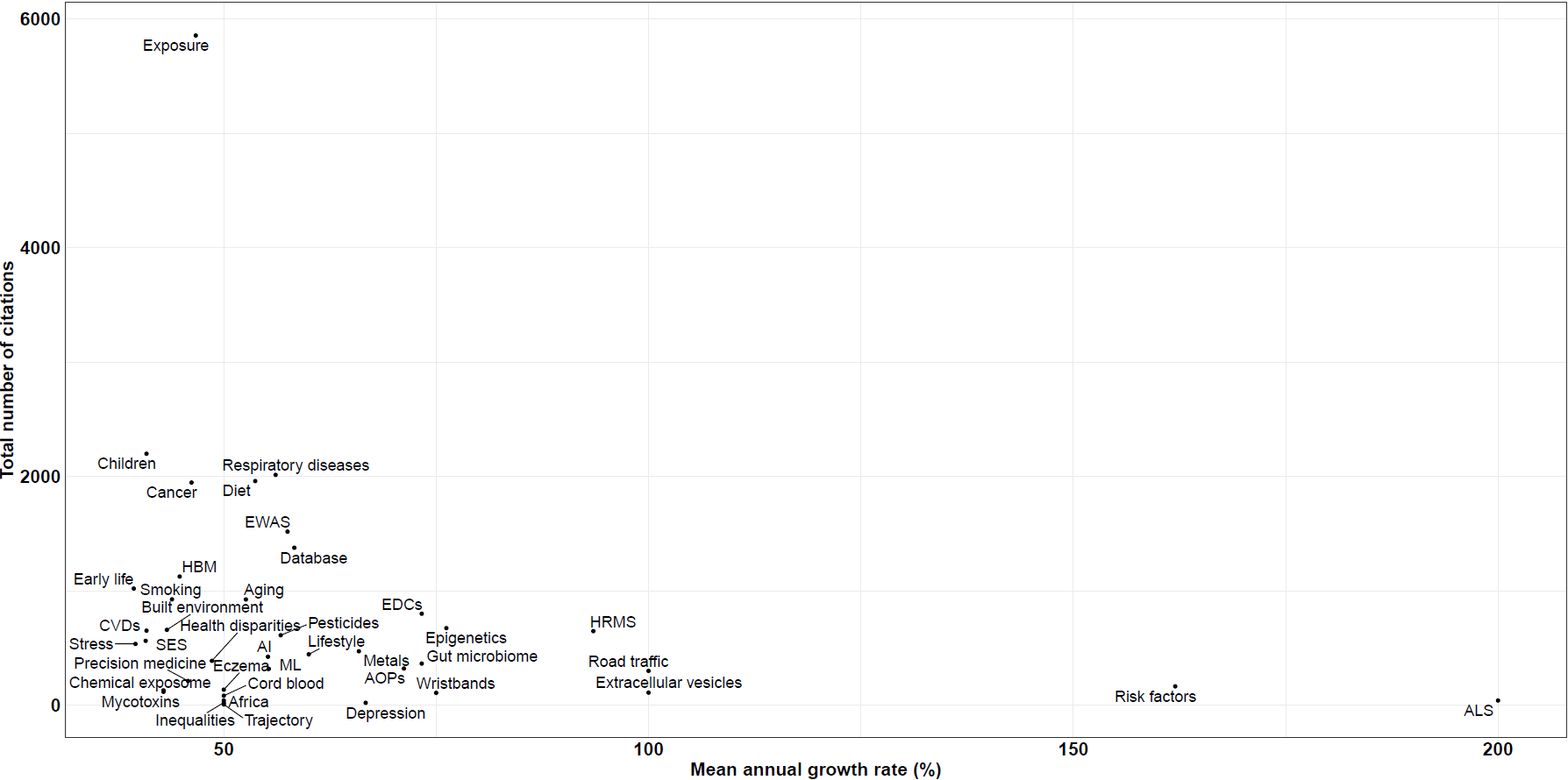


# **Fig.A.27: The top 40 keywords with the highest mean annual growth rate and their total number of citations**

AI: artificial intelligence, ALS: amyotrophic lateral sclerosis, AOPs: adverse outcome pathways, CVDs: cardiovascular diseases, EDCs: endocrine-disrupting chemicals, EWAS: exposome-wide association, HBM: human biological monitoring, HRMS: high-resolution mass spectrometry, ML: machine learning, SES: socio-economic status.


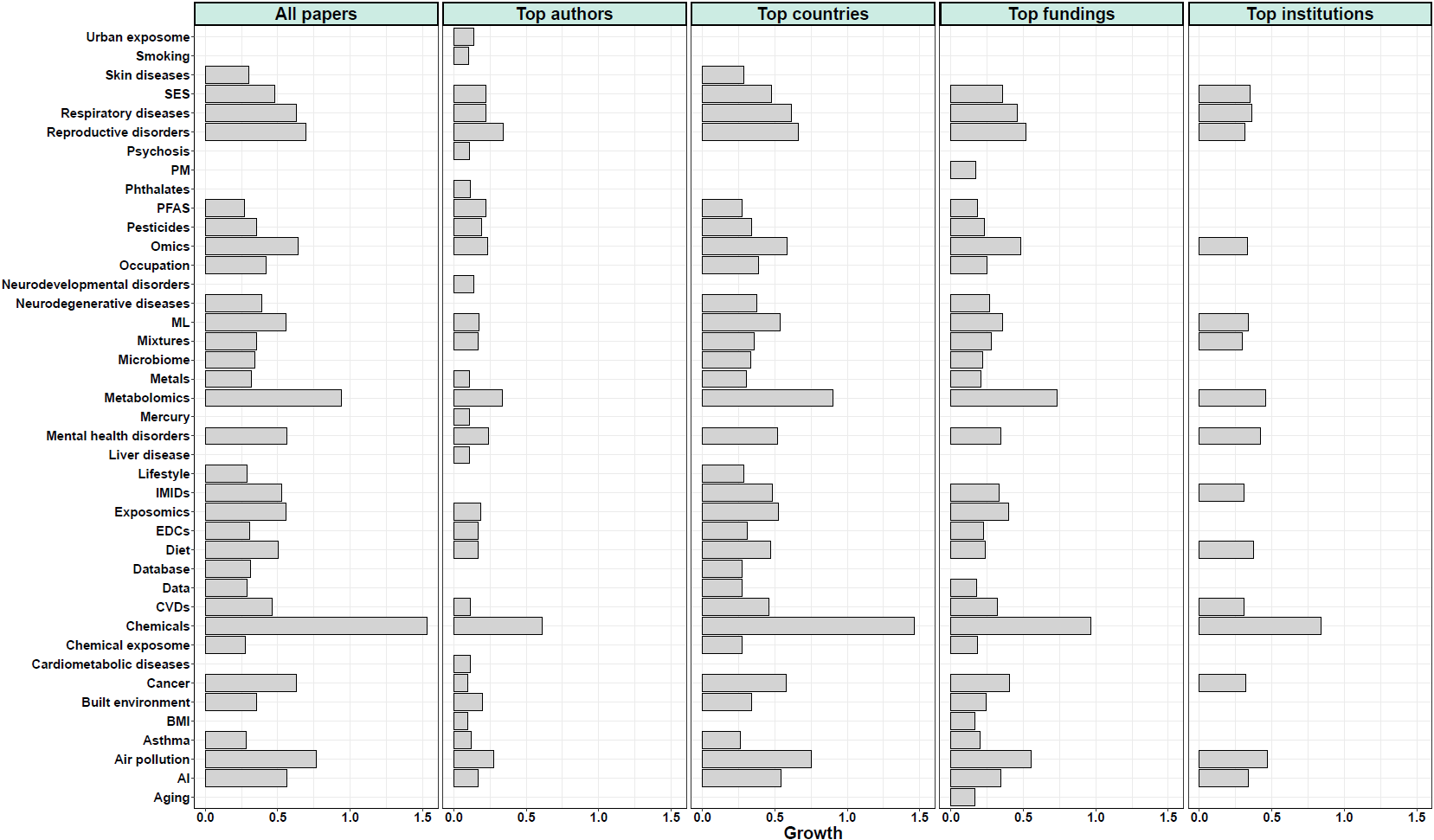


# **Fig.A.28: Keywords with the most important positive growth over time among top authors, countries, funding bodies, institutions, and journals**

AI: artificial intelligence, BMI: body mass index, CVDs: cardiovascular diseases, EDCs: endocrine-disrupting chemicals, IMIDs: immune-mediated inflammatory diseases, ML: machine learning, PFAS: per- and polyfluoroalkyl substances, PM: particulate matter, SES: socio-economic status.


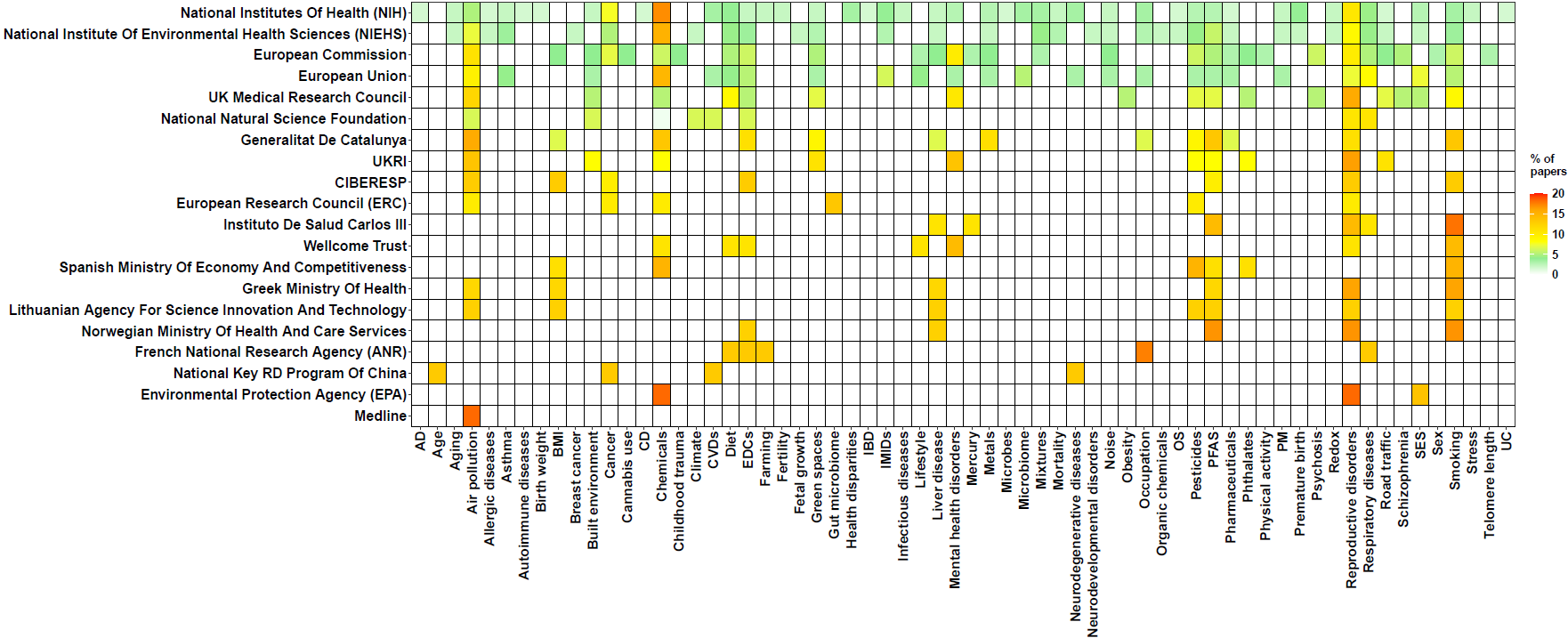


# **Fig.A.29: Exposure and health outcome-related keywords with the most important positive growth over time among top funding bodies**

AD: Alzheimer’s disease, BMI: body mass index, CD: Crohn’s disease, CVDs: cardiovascular diseases, EDCs: endocrine-disrupting chemicals, IBD: inflammatory bowel disease, IMIDs: immune-mediated inflammatory diseases, OS: oxidative stress, PFAS: per- and polyfluoroalkyl substances, PM: particulate matter, SES: socio-economic status, UC: ulcerative colitis.

**
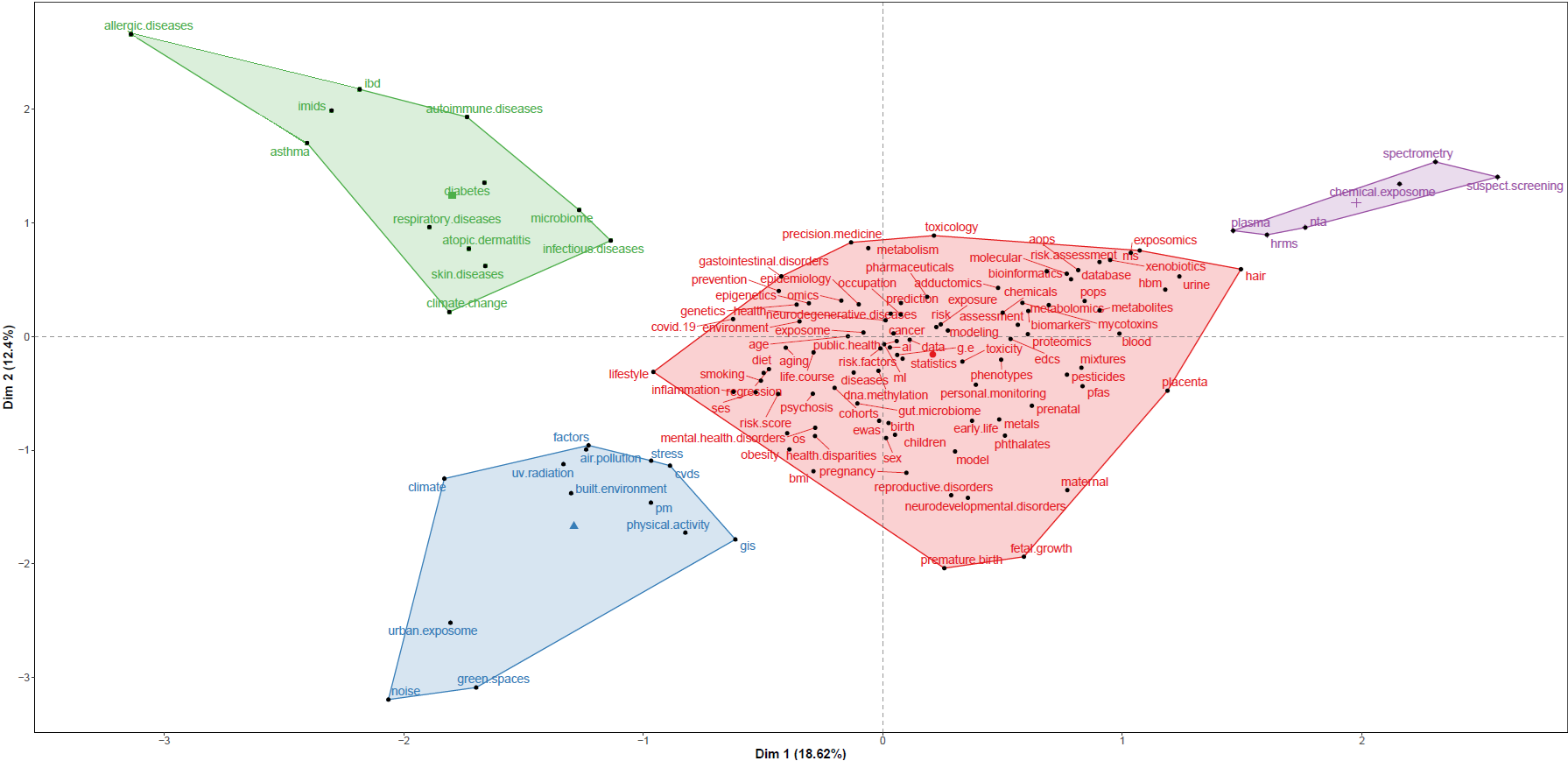
**

# **Fig.A.30: Conceptual structure map of the most frequent keywords made with multiple correspondence analysis**

AI: artificial intelligence, AOPs: adverse outcome pathways, BMI: body mass index, CVDs: cardiovascular diseases, DNA: deoxyribonucleic acid, EDCs: endocrine-disrupting chemicals, EWAS: exposome-wide association study, G.E: gene-environment interaction, GIS: geographic information system, HBM: human biomonitoring, HRMS: high-resolution mass spectrometry, IBD: inflammatory bowel disease, IMIDs: immune-mediated inflammatory diseases, ML: machine learning, MS: mass spectrometry, NTA: non-targeted analysis, OS: oxidative stress, PFAS: per- and polyfluoroalkyl substances, PM: particulate matter, POPs: persistent organic pollutants, SES: socio-economic status, UV: ultraviolet.


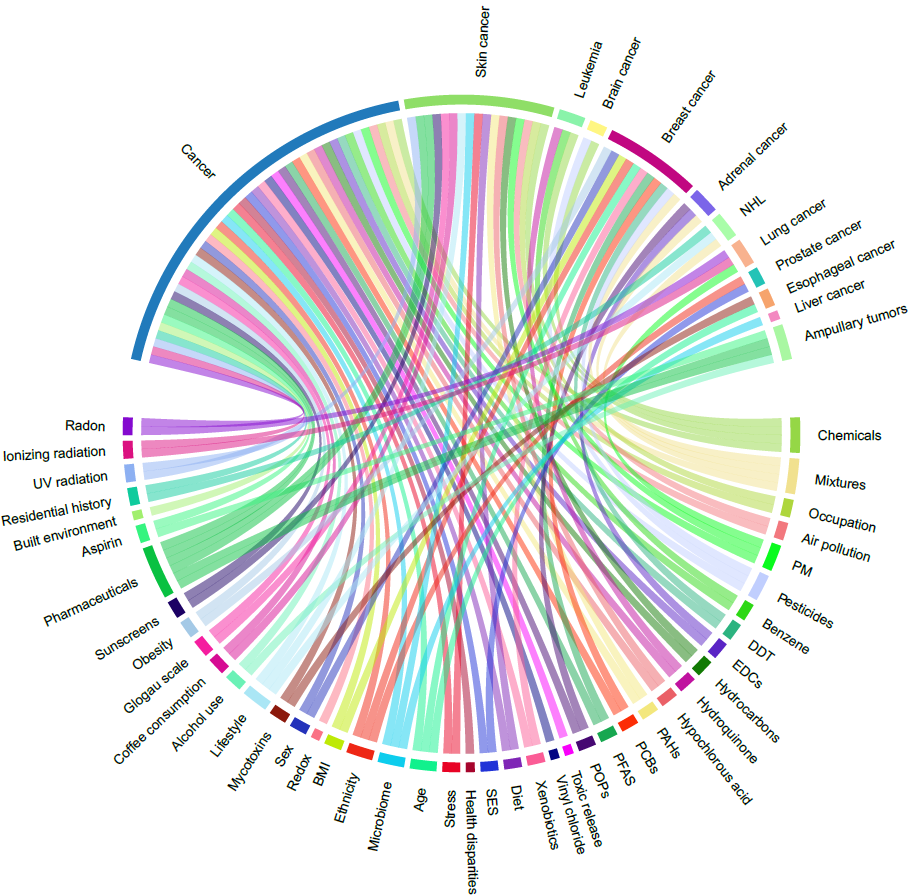


# **Fig.A.31:** **Chord diagram of keyword co-occurrence between potential risk factor and cancer keywords**

BMI: body mass index, DDT: dichlorodiphenyltrichloroethane, EDCs: endocrine-disrupting chemicals, NHL: non-Hodgkin lymphoma, PAHs: polycyclic aromatic hydrocarbons, PCBs: polychlorinated biphenyls, PFAS: per- and polyfluoroalkyl substances, PM: particulate matter, POPs: persistent organic pollutants, SES: socio-economic status, UV: ultraviolet.

**
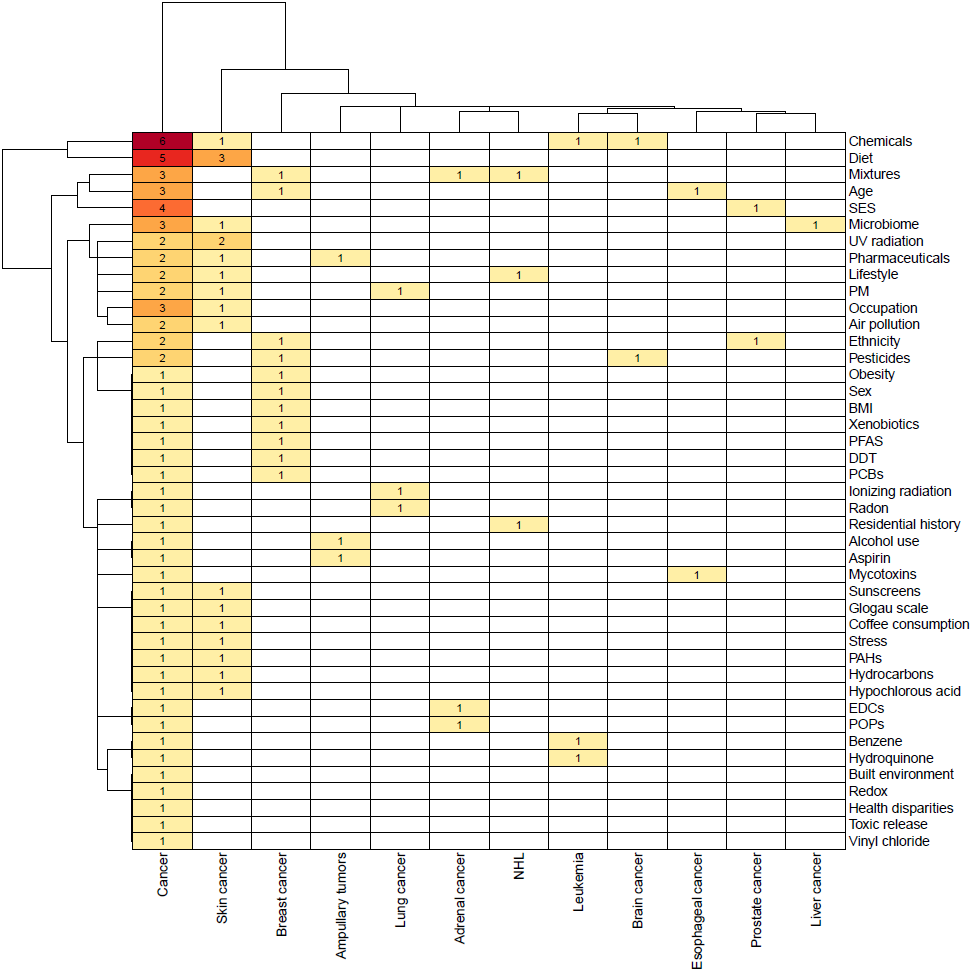
**

# **Fig.A.32: Heatmap of keyword co-occurrence between potential risk factor and cancer keywords**

BMI: body mass index, DDT: dichlorodiphenyltrichloroethane, EDCs: endocrine-disrupting chemicals, NHL: non-Hodgkin lymphoma, PAHs: polycyclic aromatic hydrocarbons, PCBs: polychlorinated biphenyls, PFAS: per- and polyfluoroalkyl substances, PM: particulate matter, POPs: persistent organic pollutants, SES: socio-economic status, UV: ultraviolet.


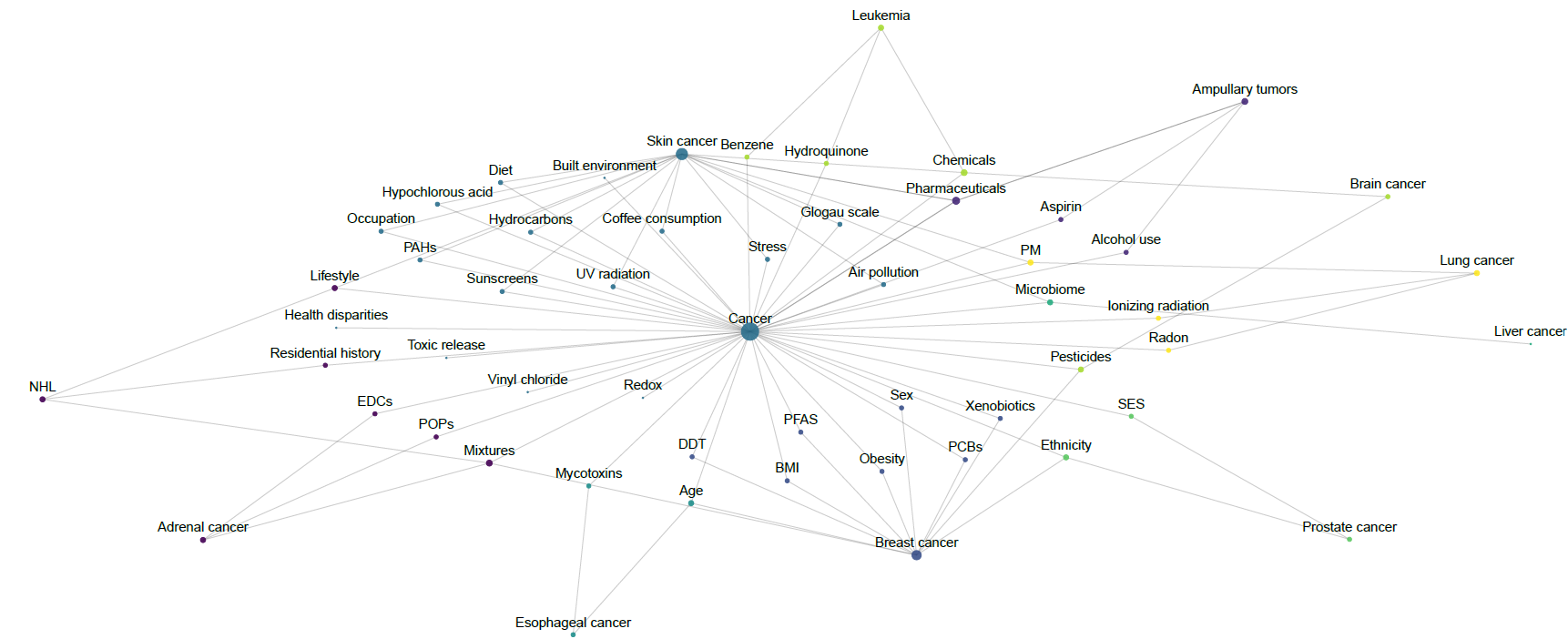


# **Fig.A.33: Keyword co-occurrence network between potential risk factor and cancer keywords**

BMI: body mass index, DDT: dichlorodiphenyltrichloroethane, EDCs: endocrine-disrupting chemicals, NHL: non-Hodgkin lymphoma, PAHs: polycyclic aromatic hydrocarbons, PCBs: polychlorinated biphenyls, PFAS: per- and polyfluoroalkyl substances, PM: particulate matter, POPs: persistent organic pollutants, SES: socio-economic status, UV: ultraviolet.


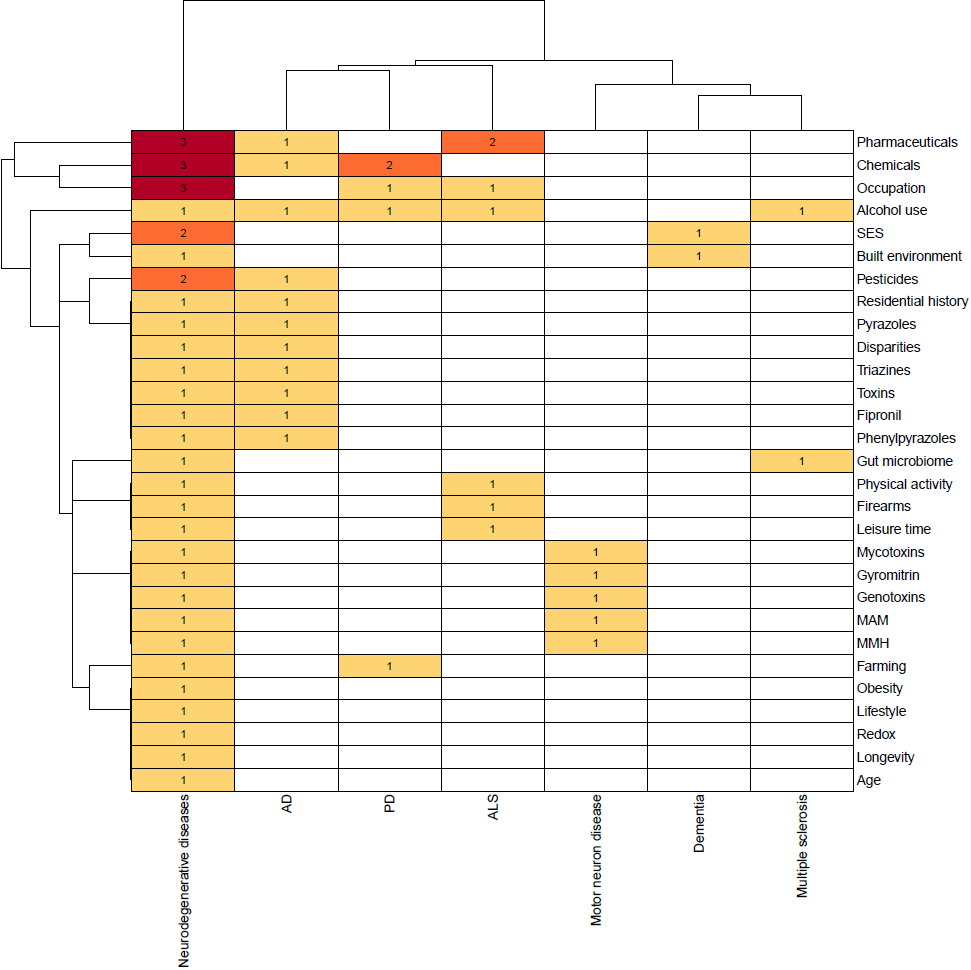


# **Fig.A.34: Heatmap of keyword co-occurrence between potential risk factor and neurodegenerative disease keywords**

AD: Alzheimer’s disease, ALS: amyotrophic lateral sclerosis, MAM: methylazoxymethanol, MMH: monomethylhydrazine, PD: Parkinson’s disease, SES: socio-economic status.


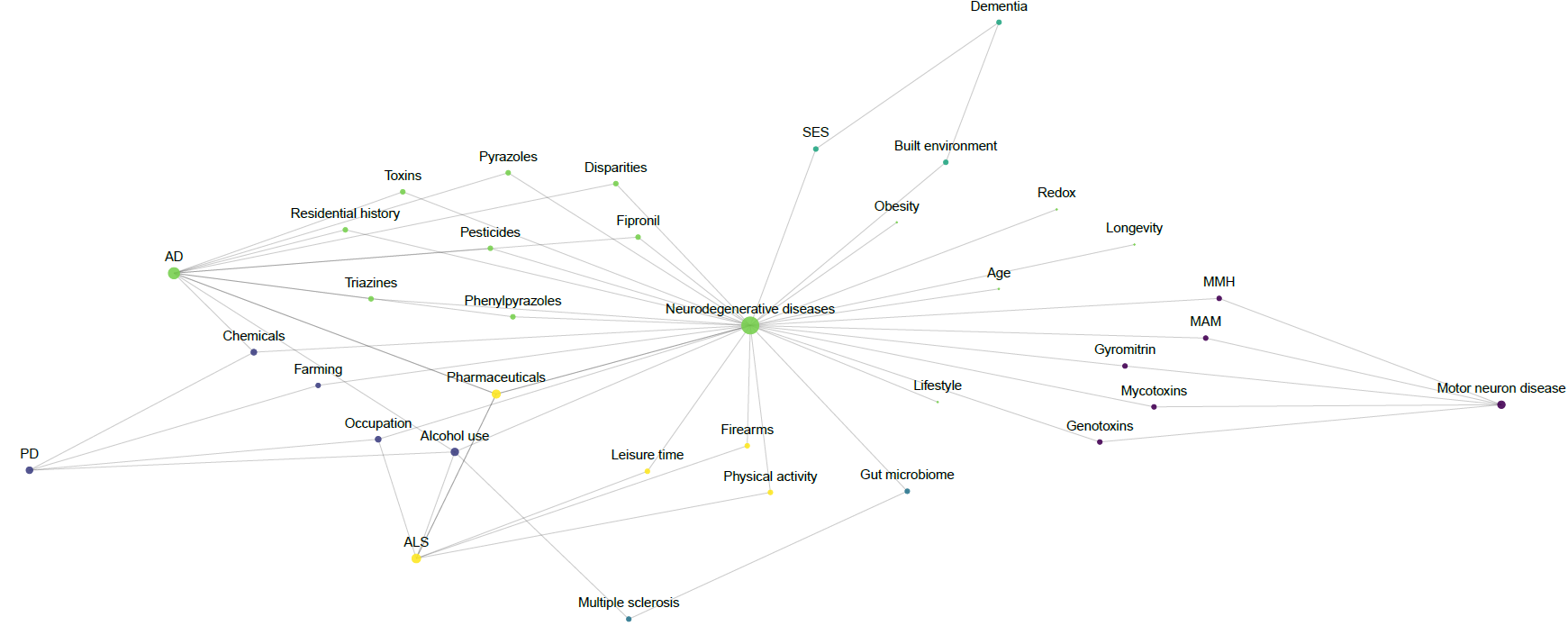


# **Fig.A.35: Keyword co-occurrence network between potential risk factor and neurodegenerative disease keywords**

AD: Alzheimer’s disease, ALS: amyotrophic lateral sclerosis, MAM: methylazoxymethanol, MMH: monomethylhydrazine, PD: Parkinson’s disease, SES: socio-economic status.


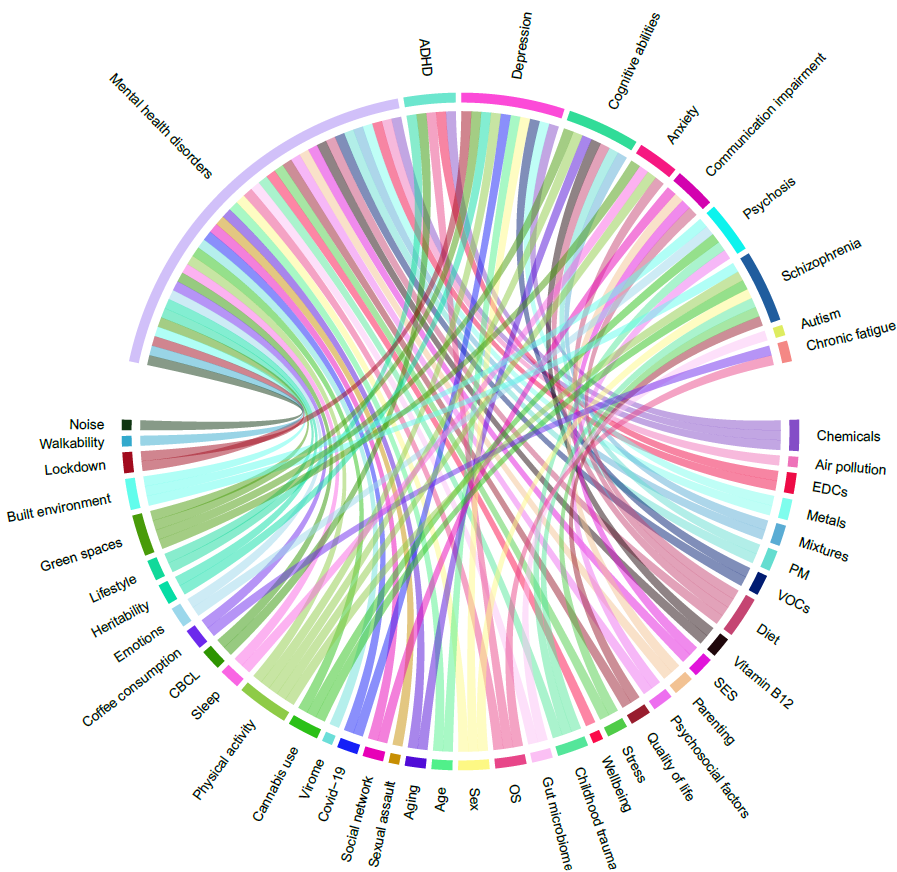


# **Fig.A.36: Chord diagram of keyword co-occurrence between potential risk factor and mental disorder keywords**

ADHD: attention deficit hyperactivity disorder, CBCL: child behavior checklist, EDCs: endocrine-disrupting chemicals, OS: oxidative stress, PM: particulate matter, SES: socioeconomic status, VOCs: volatile organic compounds.


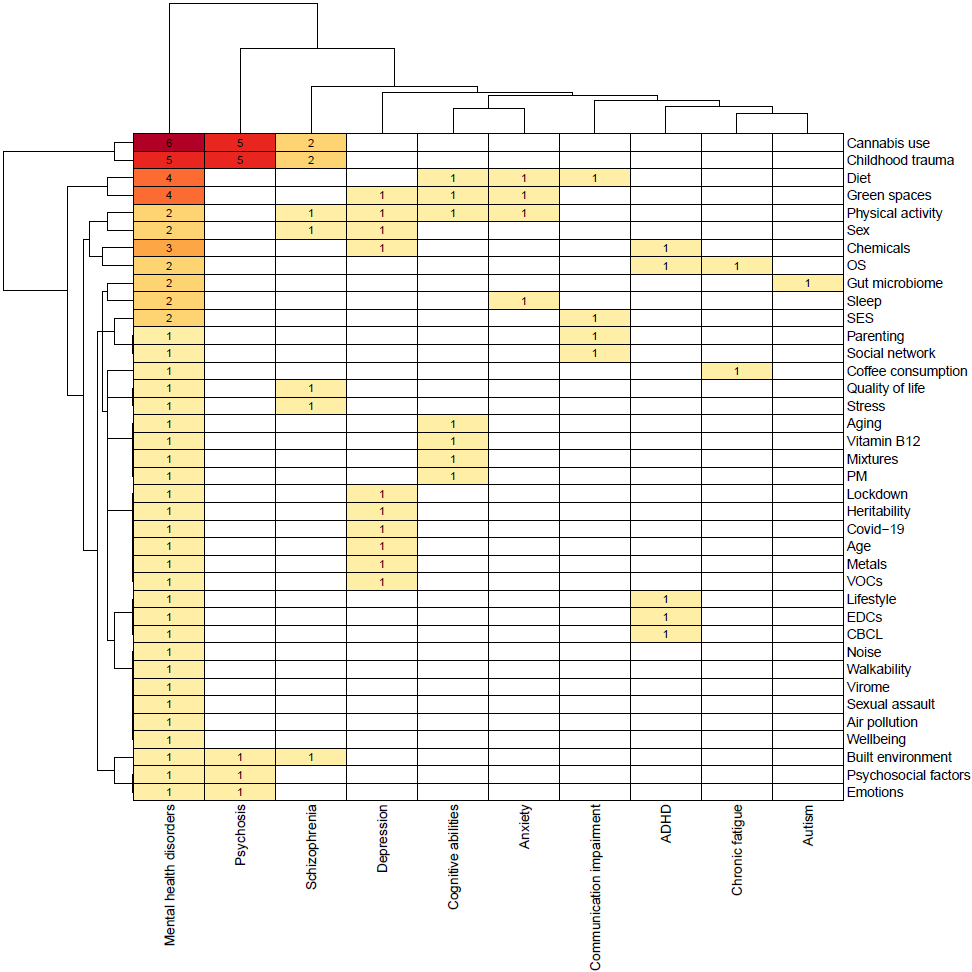


# **Fig.A.37: Heatmap of keyword co-occurrence between potential risk factor and mental disorder keywords**

ADHD: attention deficit hyperactivity disorder, CBCL: child behavior checklist, EDCs: endocrine-disrupting chemicals, OS: oxidative stress, PM: particulate matter, SES: socioeconomic status, VOCs: volatile organic compounds. The number in each cell corresponds to the number of co-occurrences.


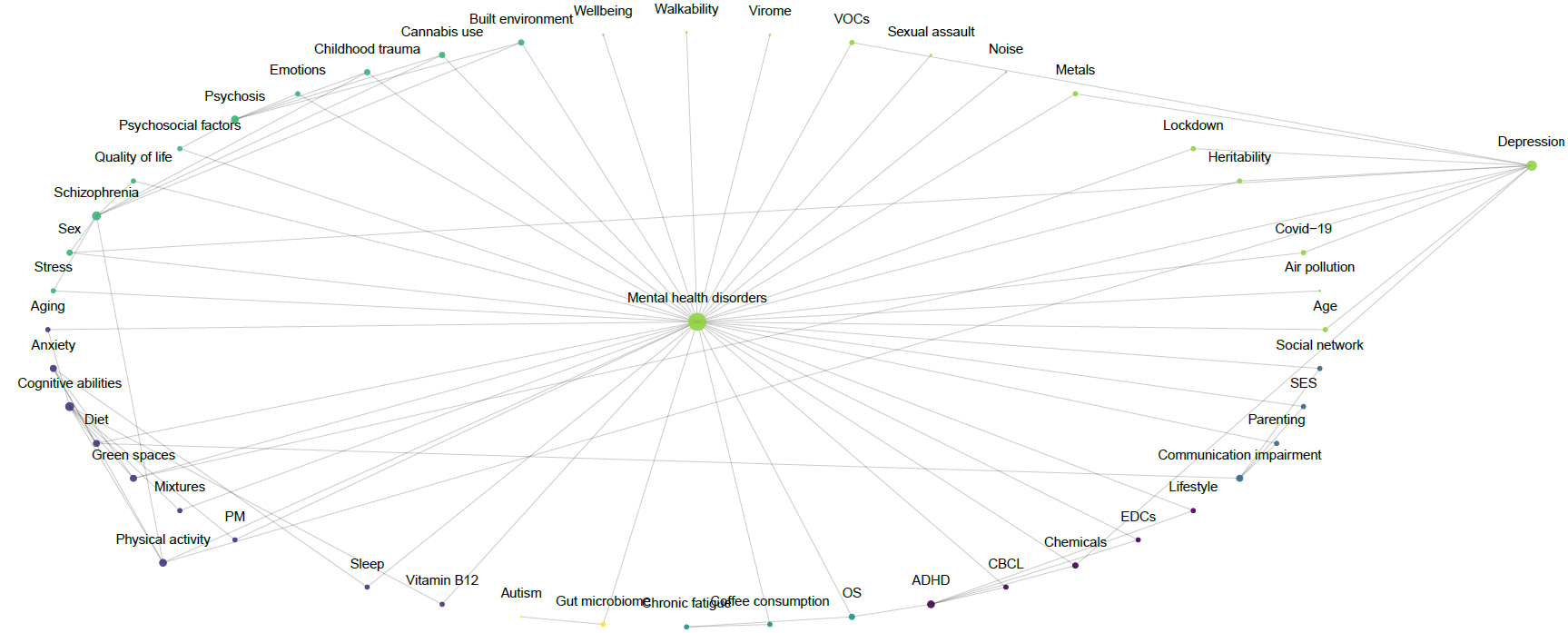


# **Fig.A.38: Keyword co-occurrence network between potential risk factor and mental disorder keywords**

ADHD: attention deficit hyperactivity disorder, CBCL: child behavior checklist, EDCs: endocrine-disrupting chemicals, OS: oxidative stress, PM: particulate matter, SES: socioeconomic status, VOCs: volatile organic compounds.


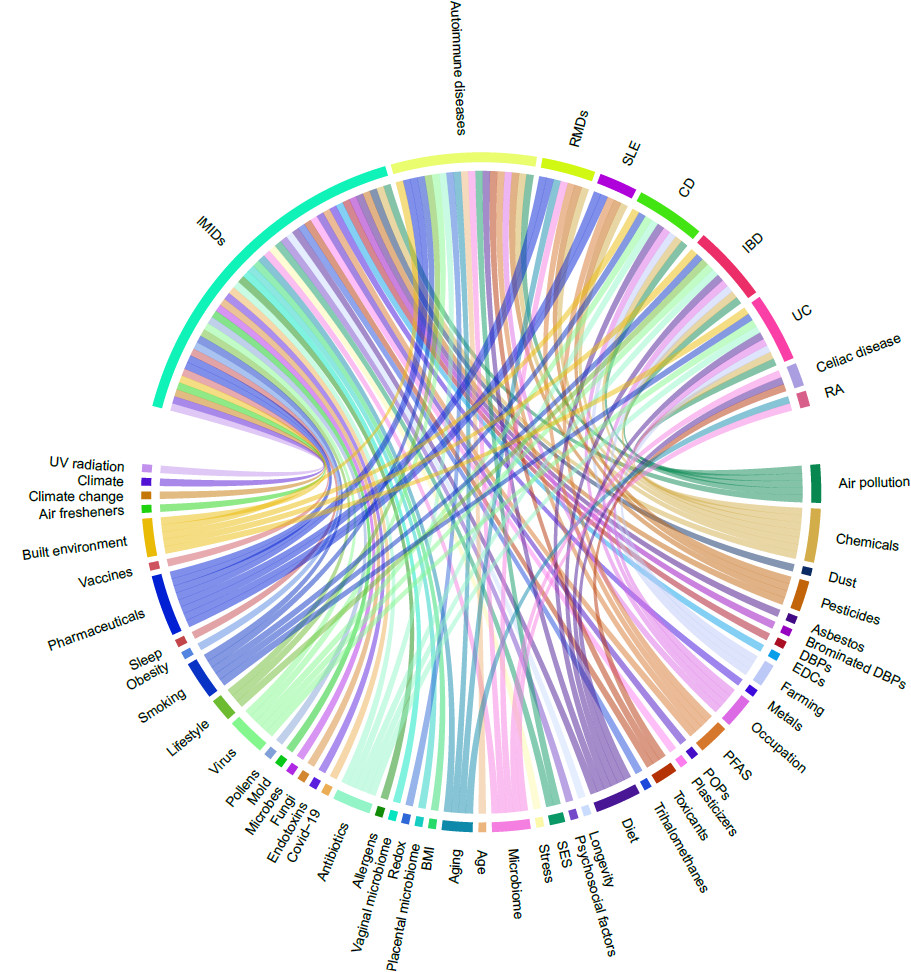


# **Fig.A.39: Chord diagram of keyword co-occurrence between potential risk factor and autoimmune disease keywords**

CD: Crohn’s disease, DBPs: disinfection by-products, EDCs: endocrine-disrupting chemicals, IBDs: inflammatory bowel diseases, IMIDs: immune-mediated inflammatory diseases, PFAS: per- and polyfluoroalkyl substances, POPs: persistent organic pollutants, RA: rheumatoid arthritis, RMDs: rheumatic and musculoskeletal diseases, SLE: systemic lupus erythematosus, SES: socio-economic status, UC: ulcerative colitis, UV: ultraviolet.


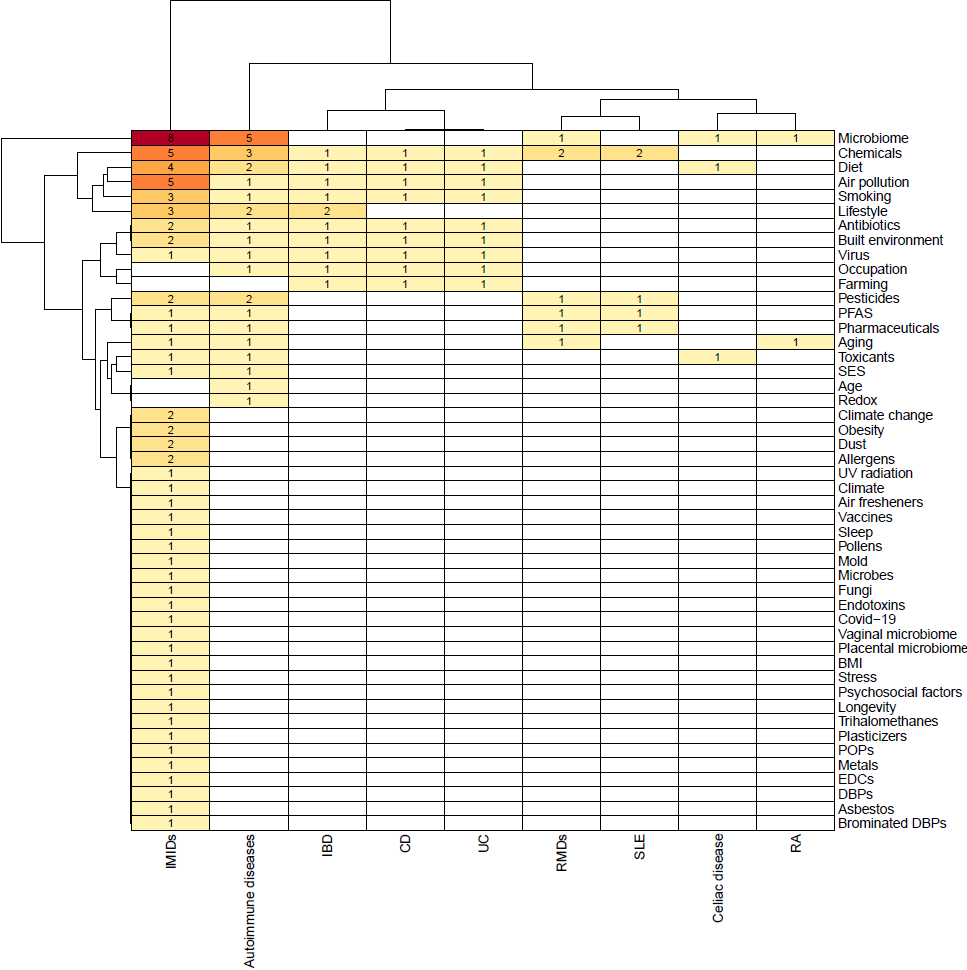


# **Fig.A.40: Heatmap of keyword co-occurrence between potential risk factor and autoimmune disease keywords**

CD: Crohn’s disease, DBP: disinfection by-products, EDCs: endocrine-disrupting chemicals, IBDs: inflammatory bowel diseases, IMIDs: immune-mediated inflammatory diseases, PFAS: per- and polyfluoroalkyl substances, POPs: persistent organic pollutants, RA: rheumatoid arthritis, RMDs: rheumatic and musculoskeletal diseases, SLE: systemic lupus erythematosus, SES: socio-economic status, UC: ulcerative colitis, UV: ultraviolet. The number in each cell corresponds to the number of co-occurrences.


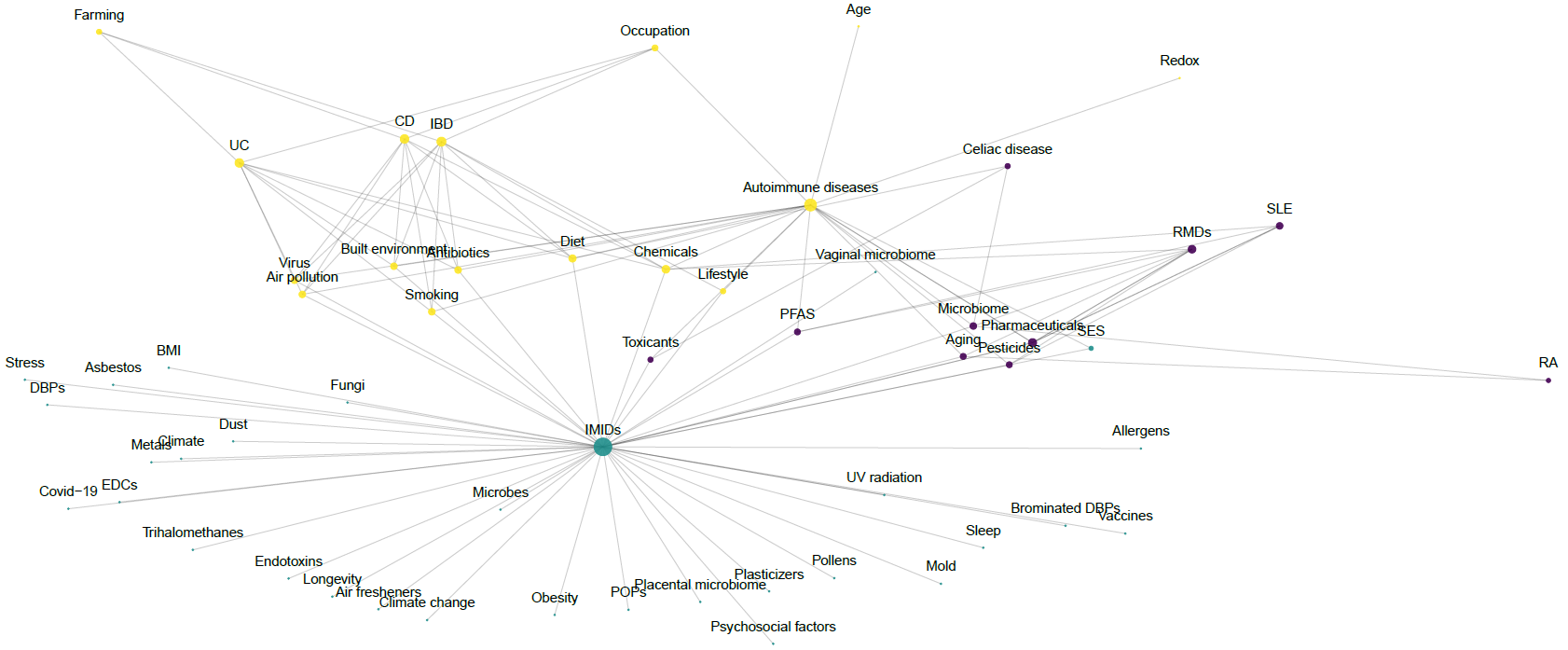


# **Fig.A.41: Keyword co-occurrence network between potential risk factor and autoimmune disease keywords**

CD: Crohn’s disease, DBP: disinfection by-products, EDCs: endocrine-disrupting chemicals, IBDs: inflammatory bowel diseases, IMIDs: immune-mediated inflammatory diseases, PFAS: per- and polyfluoroalkyl substances, POPs: persistent organic pollutants, RA: rheumatoid arthritis, RMDs: rheumatic and musculoskeletal diseases, SLE: systemic lupus erythematosus, SES: socio-economic status, UC: ulcerative colitis, UV: ultraviolet.


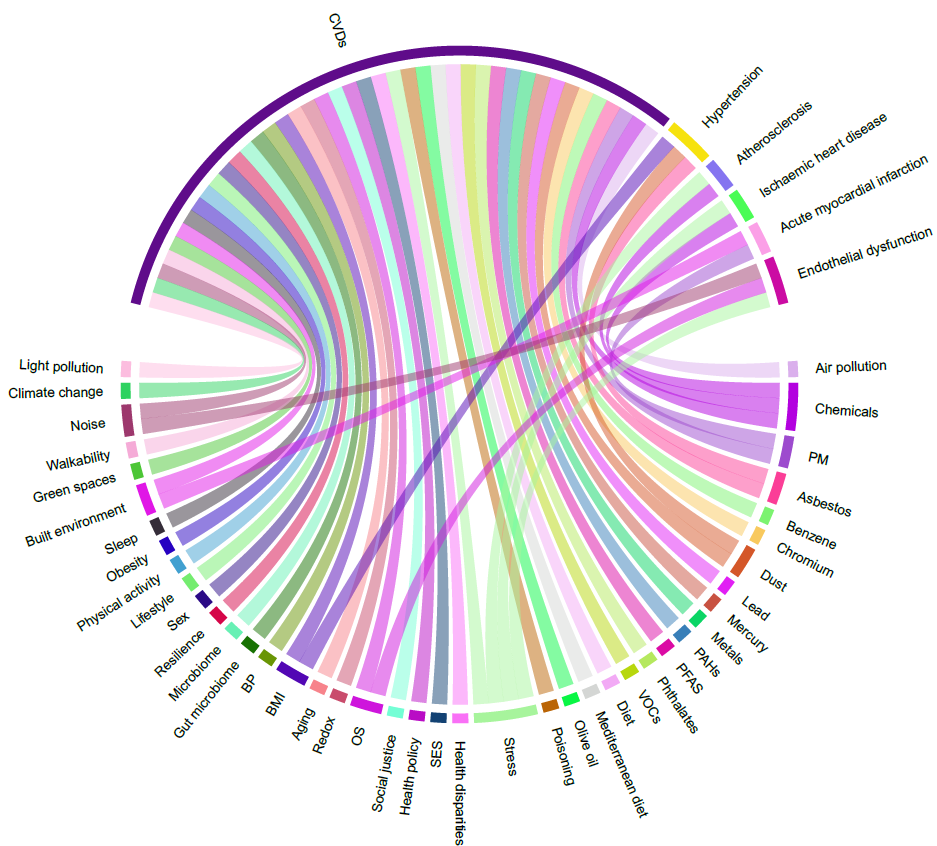


# **Fig.A.42: Chord diagram of keyword co-occurrence between potential risk factor and cardiovascular disease keywords**

BP: blood pressure, BMI: body mas index, CVDs: cardiovascular diseases, OS: oxidative stress, PAHs: polycyclic aromatic hydrocarbons, PFAS: per- and polyfluoroalkyl substances, PM: particulate matter, POPs: persistent organic pollutants, SES: socio-economic status, VOCs: volatile organic compounds.


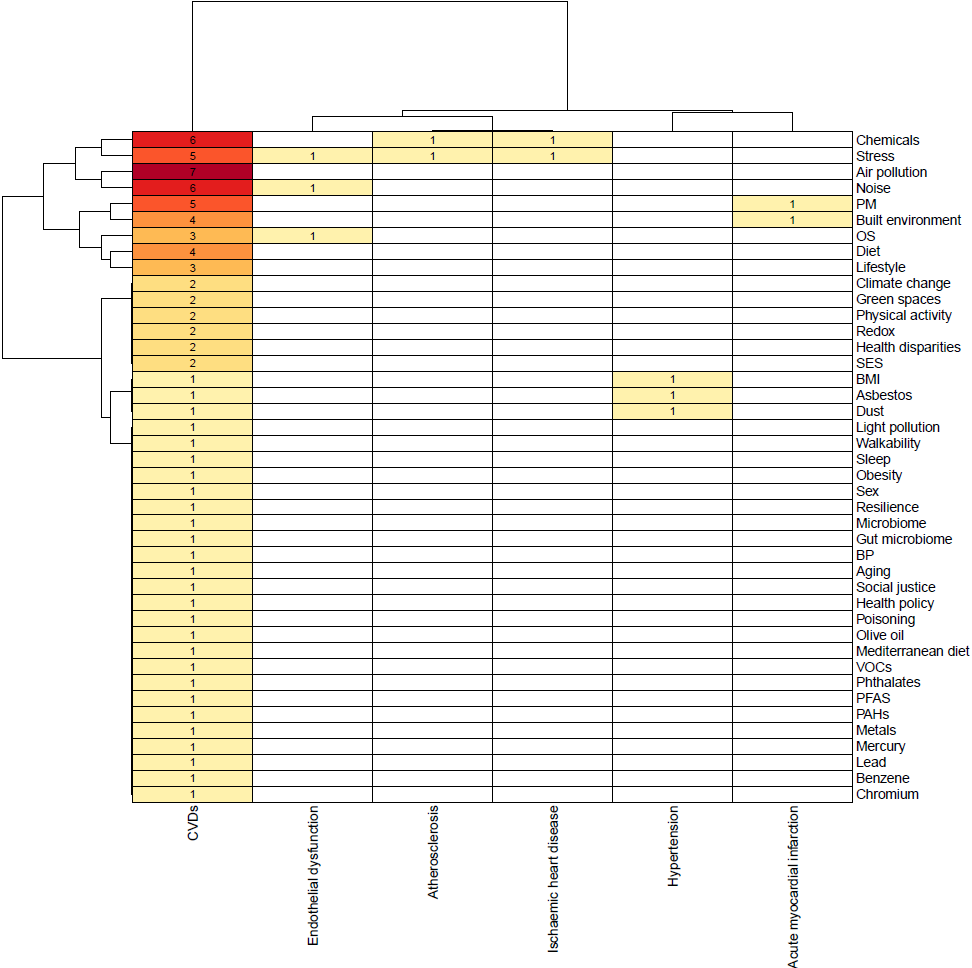


# **Fig.A.43: Heatmap of keyword co-occurrence between risk potential factor and cardiovascular disease keywords**

BP: blood pressure, BMI: body mas index, CVDs: cardiovascular diseases, OS: oxidative stress, PAHs: polycyclic aromatic hydrocarbons, PFAS: per- and polyfluoroalkyl substances, PM: particulate matter, POPs: persistent organic pollutants, SES: socio-economic status, VOCs: volatile organic compounds. The number in each cell corresponds to the number of co-occurrences.


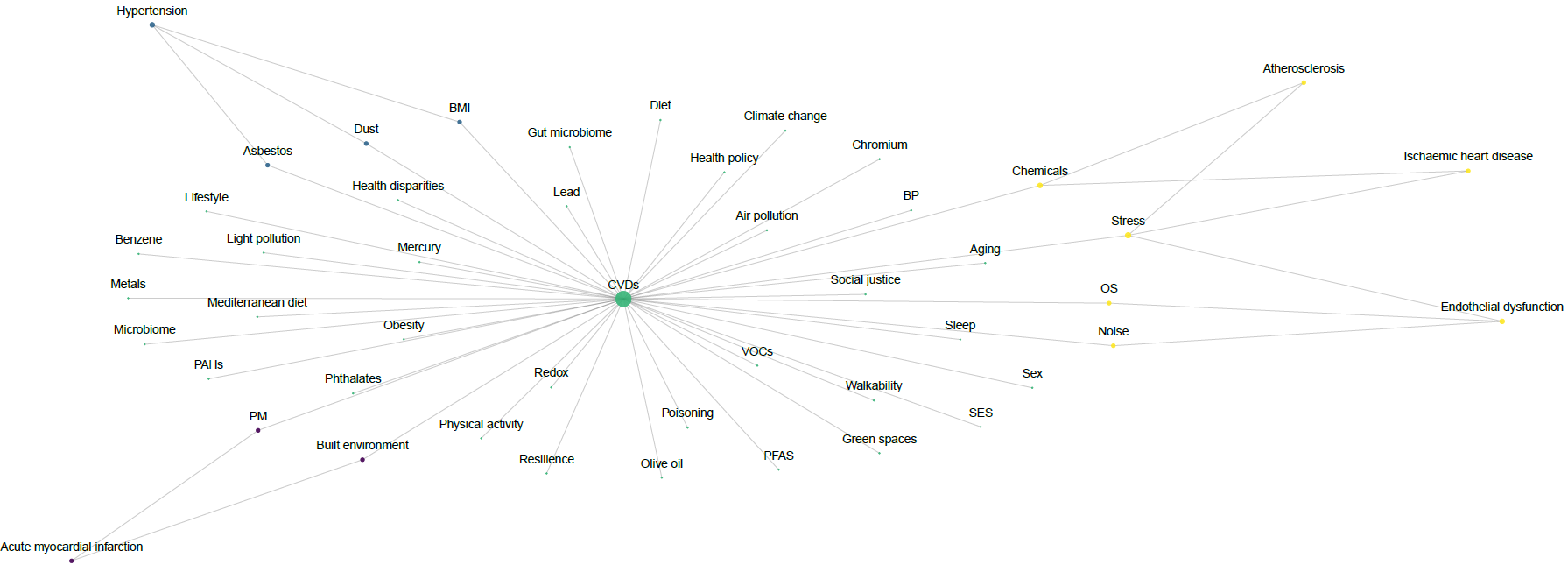


# **Fig.A.44: Keyword co-occurrence network between potential risk factor and cardiovascular disease keywords**

BP: blood pressure, BMI: body mas index, CVDs: cardiovascular diseases, OS: oxidative stress, PAHs: polycyclic aromatic hydrocarbons, PFAS: per- and polyfluoroalkyl substances, PM: particulate matter, POPs: persistent organic pollutants, SES: socio-economic status, VOCs: volatile organic compounds.


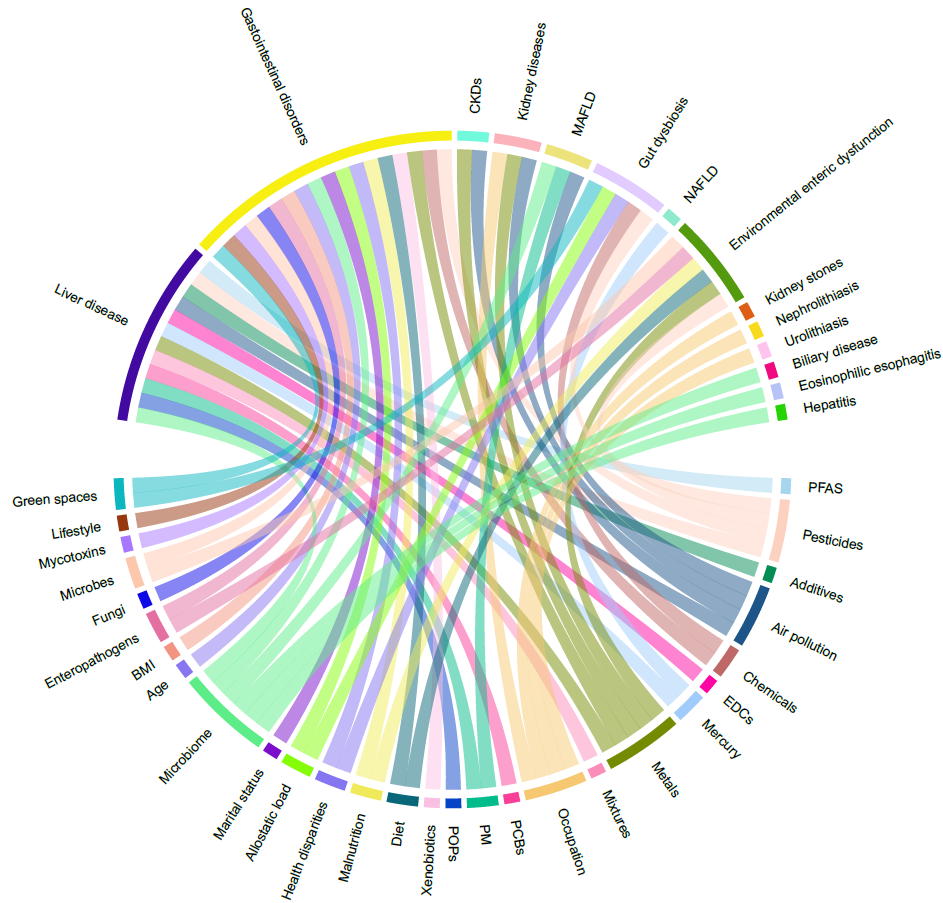


# **Fig.A.45: Chord diagram of keyword co-occurrence between potential risk factor and gastrointestinal disease keywords**

BMI: body mass index, CKD: chronic kidney disease, EDCs: endocrine-disrupting chemicals, MAFLD: metabolic associated fatty liver disease, NAFLD: metabolic dysfunction–associated steatotic liver disease, PCBs: polychlorinated biphenyls, PFAS: per- and polyfluoroalkyl substances, PM: particulate matter, POPs: persistent organic pollutants.


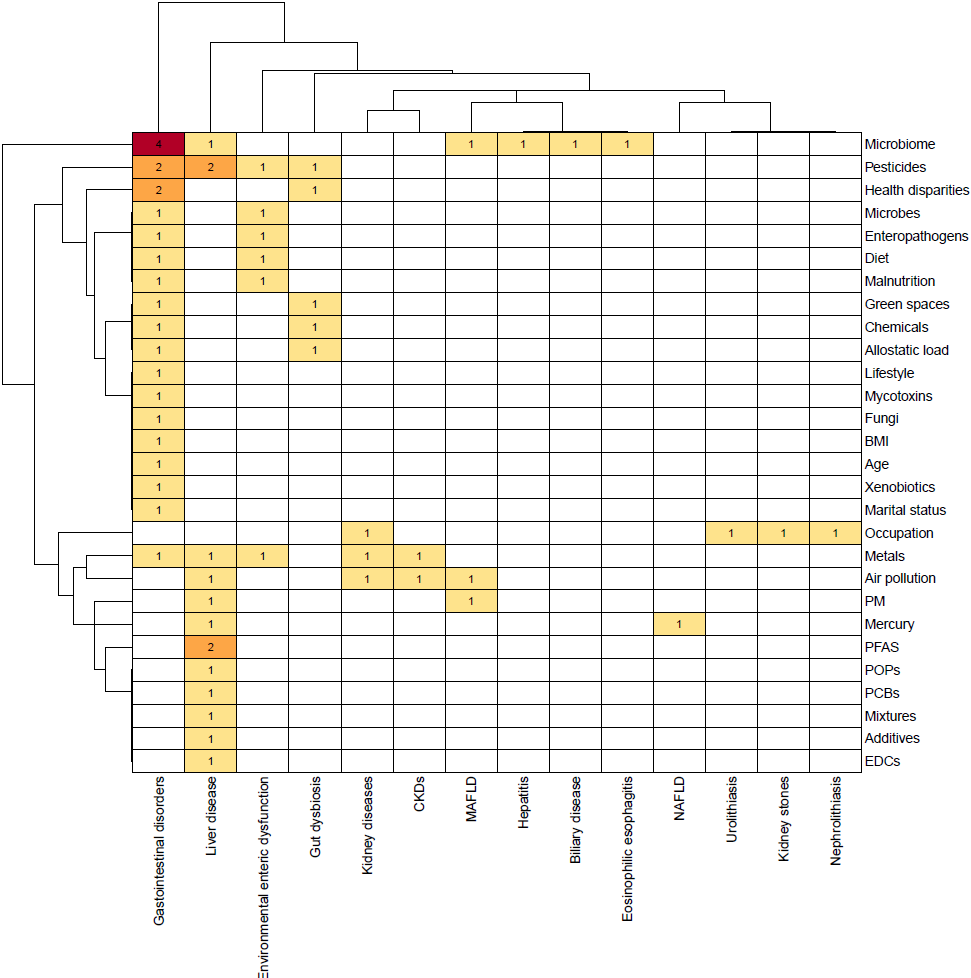


# **Fig.A.46: Heatmap of keyword co-occurrence between potential risk factor and gastrointestinal disease keywords**

BMI: body mass index, CKD: chronic kidney disease, EDCs: endocrine-disrupting chemicals, MAFLD: metabolic associated fatty liver disease, NAFLD: metabolic dysfunction–associated steatotic liver disease, PCBs: polychlorinated biphenyls, PFAS: per- and polyfluoroalkyl substances, PM: particulate matter, POPs: persistent organic pollutants. The number in each cell corresponds to the number of co-occurrences.


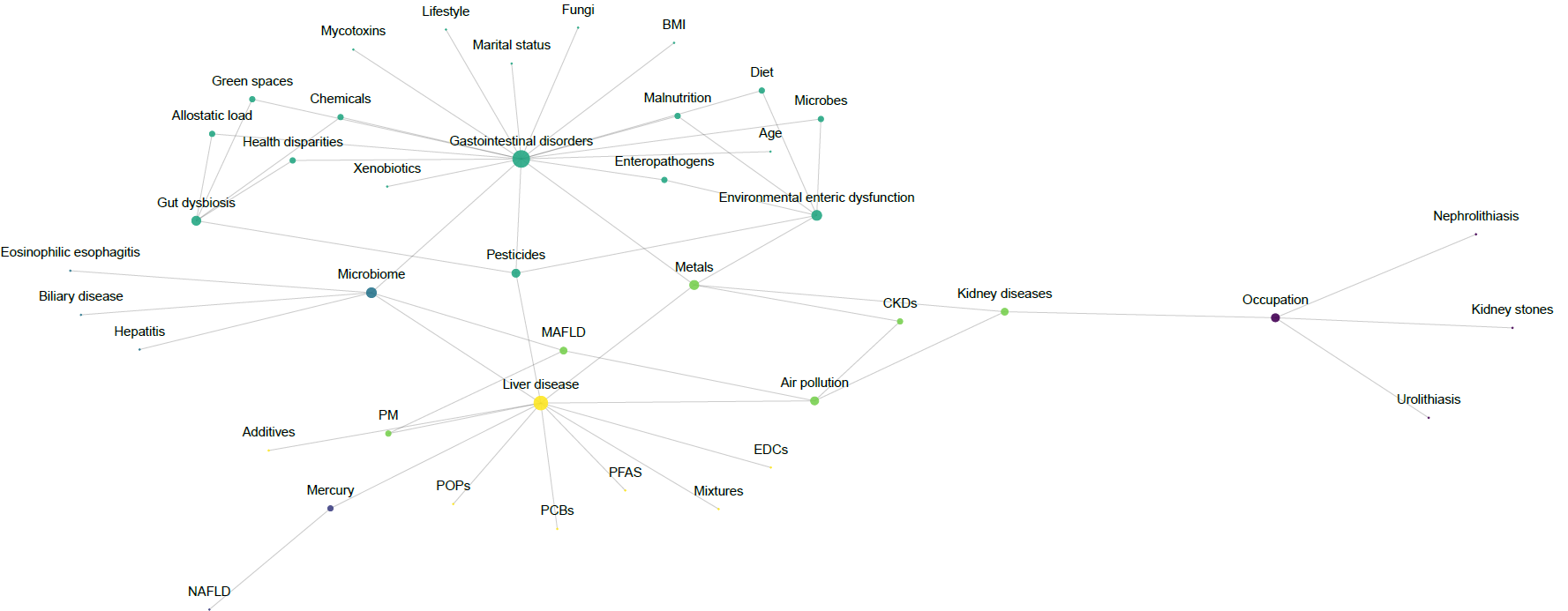


# **Fig.A.47: Keyword co-occurrence network between potential risk factor and gastrointestinal disease keywords**

BMI: body mass index, CKD: chronic kidney disease, EDCs: endocrine-disrupting chemicals, MAFLD: metabolic associated fatty liver disease, NAFLD: metabolic dysfunction–associated steatotic liver disease, PCBs: polychlorinated biphenyls, PFAS: per- and polyfluoroalkyl substances, PM: particulate matter, POPs: persistent organic pollutants.


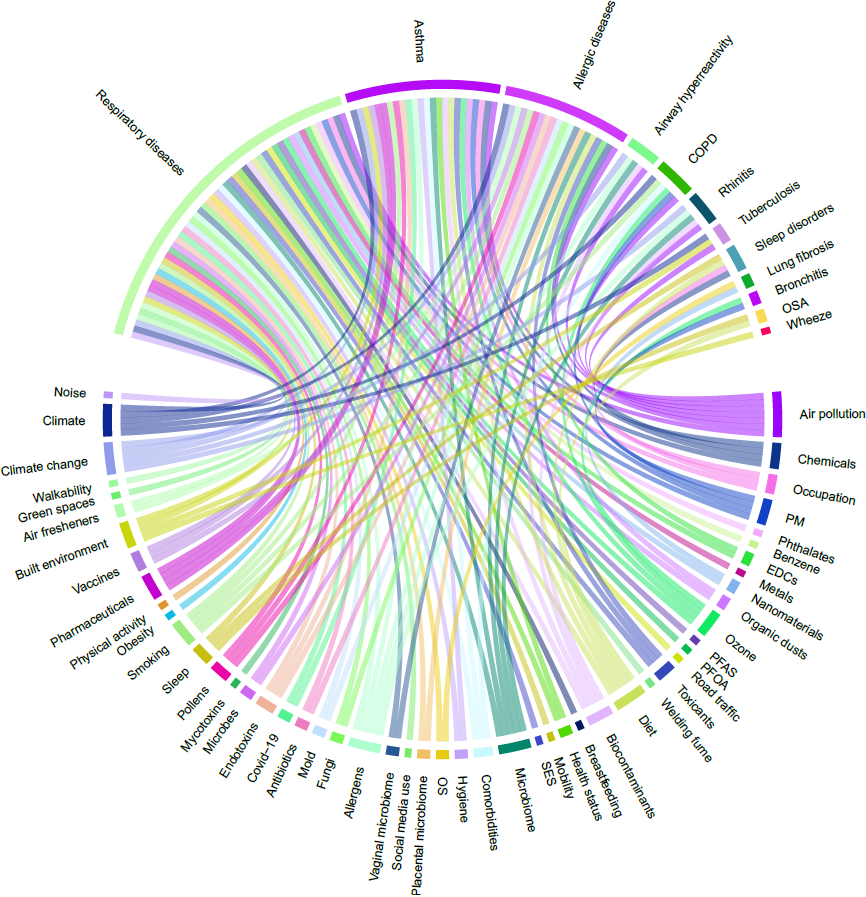


# **Fig.A.48: Chord diagram of keyword co-occurrence between potential risk factor and respiratory disease keywords**

COPD: chronic obstructive pulmonary disease, EDCs: endocrine-disrupting chemicals, PM: particulate matter, OS: oxidative stress, OSA: obstructive sleep apnea, PFAS: per- and polyfluoroalkyl substances, PFOA: perfluorooctanoic acid, SES: socio-economic status.


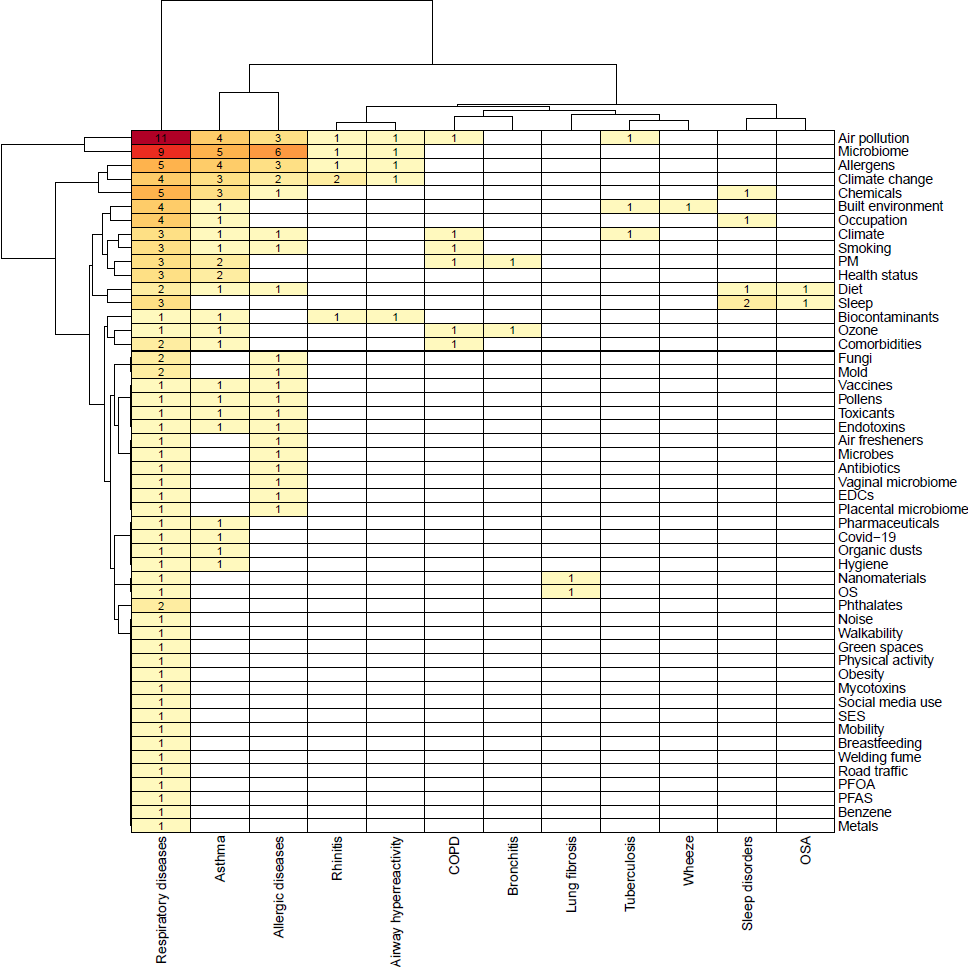


# **Fig.A.49: Heatmap of keyword co-occurrence between potential risk factor and respiratory disease keywords**

COPD: chronic obstructive pulmonary disease, EDCs: endocrine-disrupting chemicals, PM: particulate matter, OS: oxidative stress, OSA: obstructive sleep apnea, PFAS: per- and polyfluoroalkyl substances, PFOA: perfluorooctanoic acid, SES: socio-economic status. The number in each cell corresponds to the number of co-occurrences.


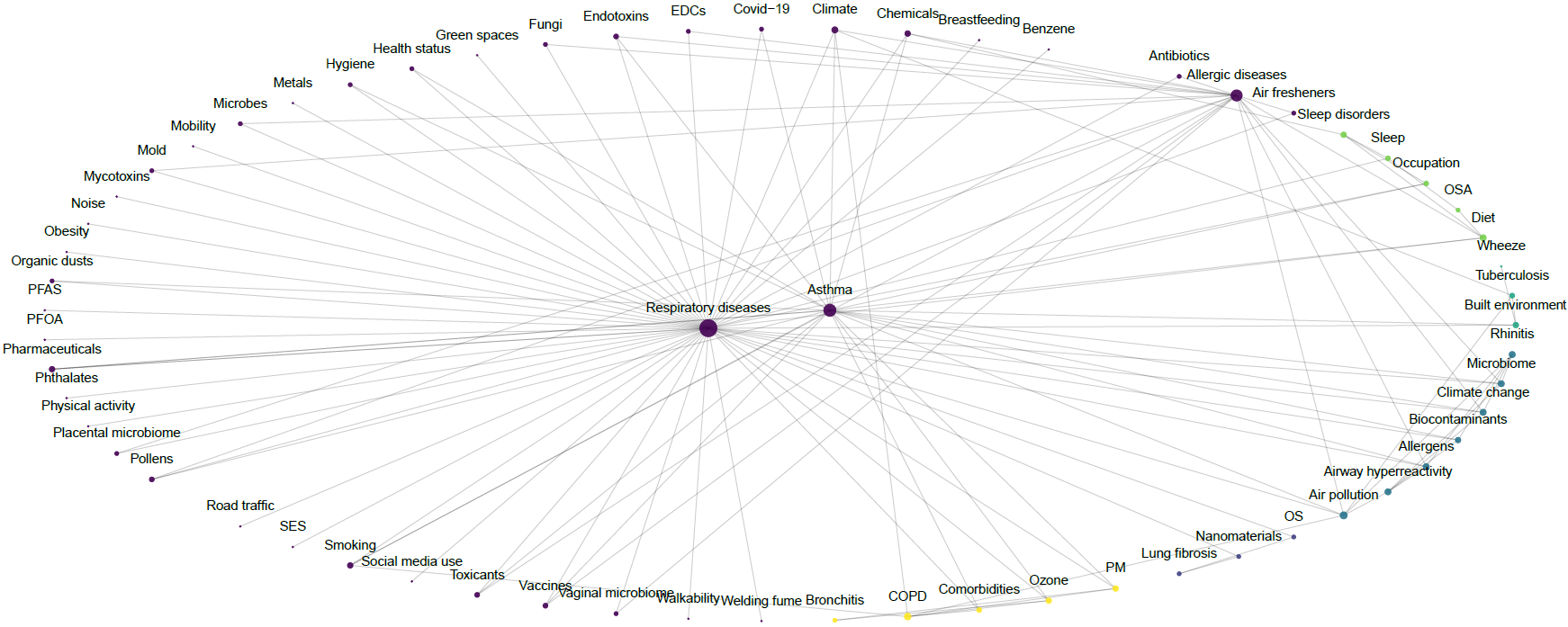


# **Fig.A.50: Keyword co-occurrence network between potential risk factor and respiratory disease keywords**

COPD: chronic obstructive pulmonary disease, EDCs: endocrine-disrupting chemicals, PM: particulate matter, OS: oxidative stress, OSA: obstructive sleep apnea, PFAS: per- and polyfluoroalkyl substances, PFOA: perfluorooctanoic acid, SES: socio-economic status.


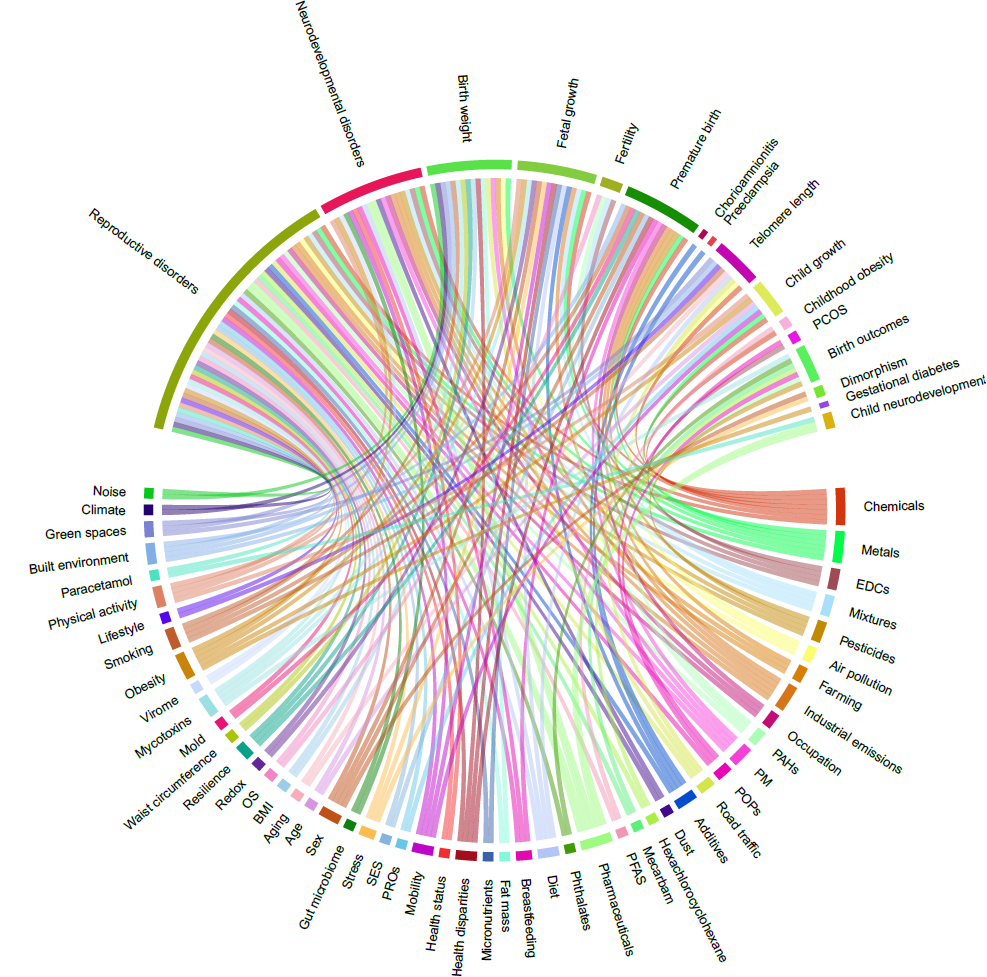


# **Fig.A.51: Chord diagram of keyword co-occurrence between potential risk factor and reproductive disease keywords**

BMI: body mass index, EDCs: endocrine-disrupting chemicals, OS: oxidative stress, PCOS: polycystic ovary syndrome, POPs: persistent organic pollutants, PROs: person reported outcomes, PAHs: polycyclic aromatic hydrocarbons, PFAS: per- and polyfluoroalkyl substances, PM: particulate matter, SES: socio-economic status.


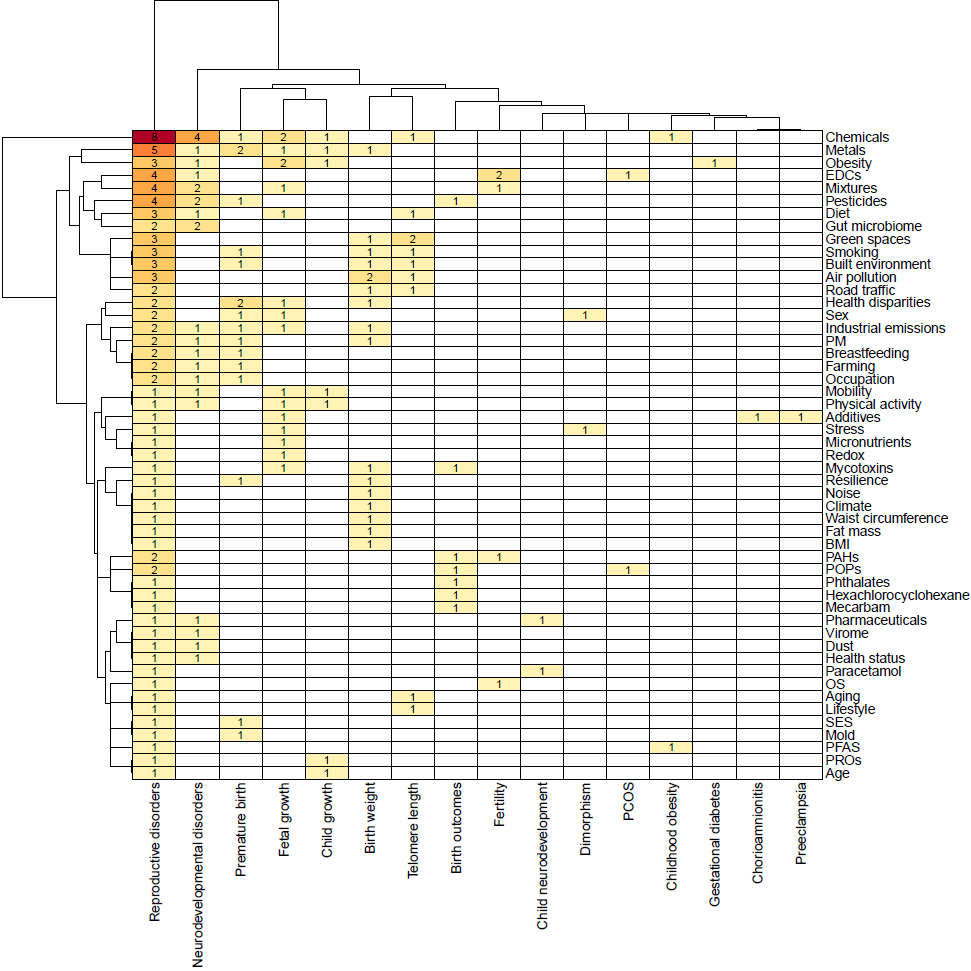


# **Fig.A.52: Heatmap of keyword co-occurrence between potential risk factor and reproductive disease keywords**

BMI: body mass index, EDCs: endocrine-disrupting chemicals, OS: oxidative stress, PCOS: polycystic ovary syndrome, POPs: persistent organic pollutants, PROs: person reported outcomes, PAHs: polycyclic aromatic hydrocarbons, PFAS: per- and polyfluoroalkyl substances, PM: particulate matter, SES: socio-economic status. The number in each cell corresponds to the number of co-occurrences.


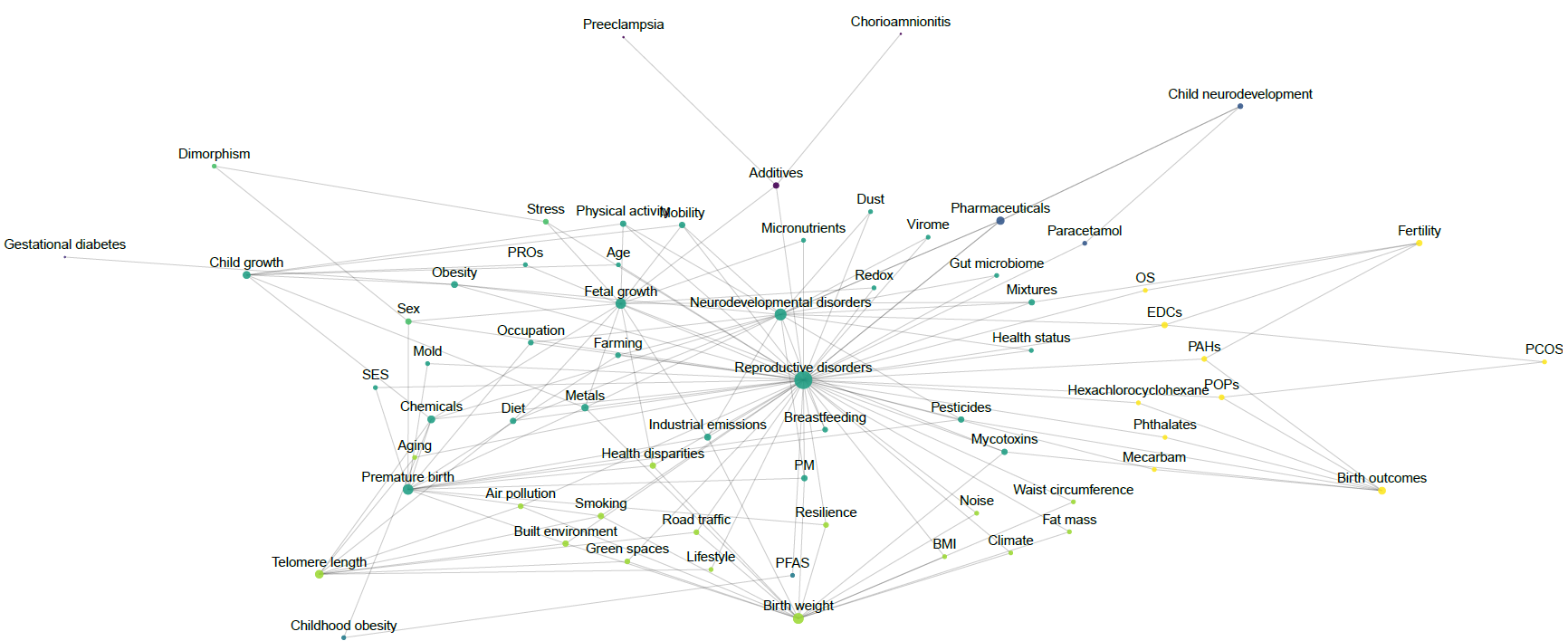


# **Fig.A.53: Keyword co-occurrence network between potential risk factor and reproductive disease keywords**

BMI: body mass index, EDCs: endocrine-disrupting chemicals, OS: oxidative stress, PCOS: polycystic ovary syndrome, POPs: persistent organic pollutants, PROs: person reported outcomes, PAHs: polycyclic aromatic hydrocarbons, PFAS: per- and polyfluoroalkyl substances, PM: particulate matter, SES: socio-economic status.


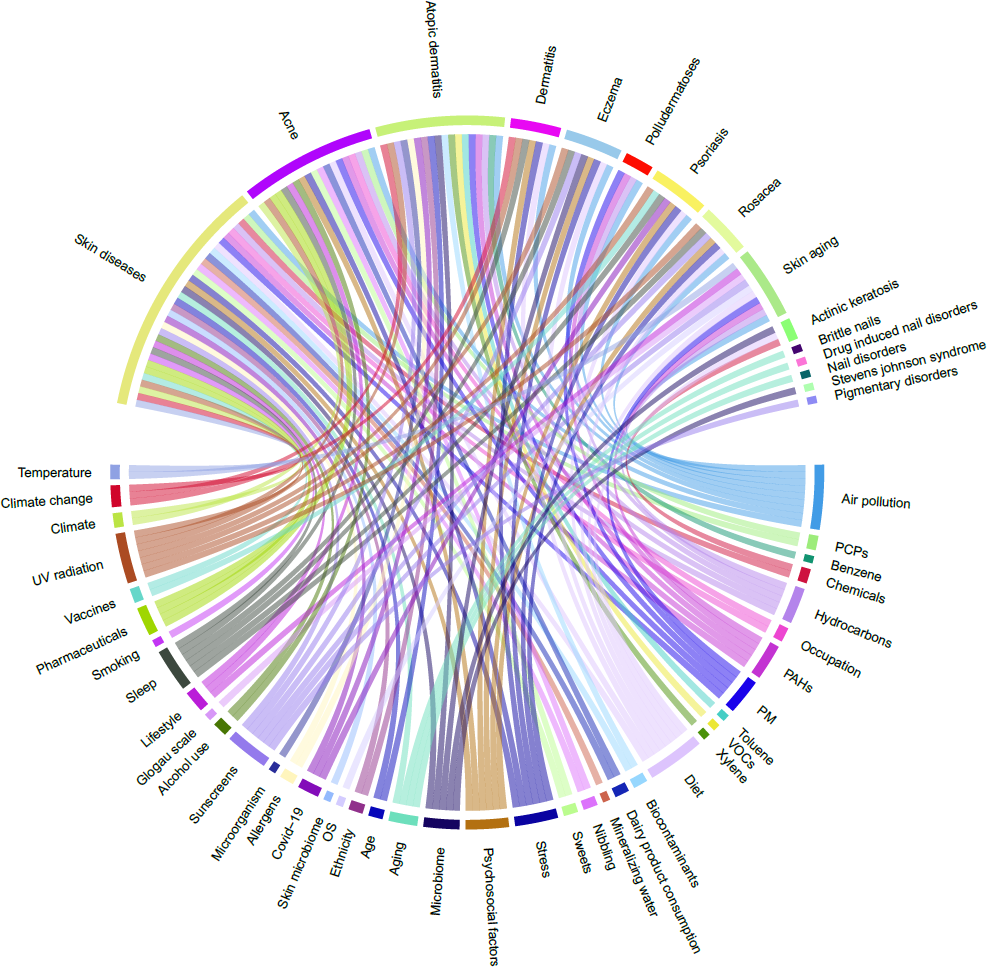


# **Fig.A.54: Chord diagram of keyword co-occurrence between potential risk factor and skin disease keywords**

OS: oxidative stress, PAHs: polycyclic aromatic hydrocarbons, PCP: personal care product, PM: particulate matter, UV: ultraviolet.


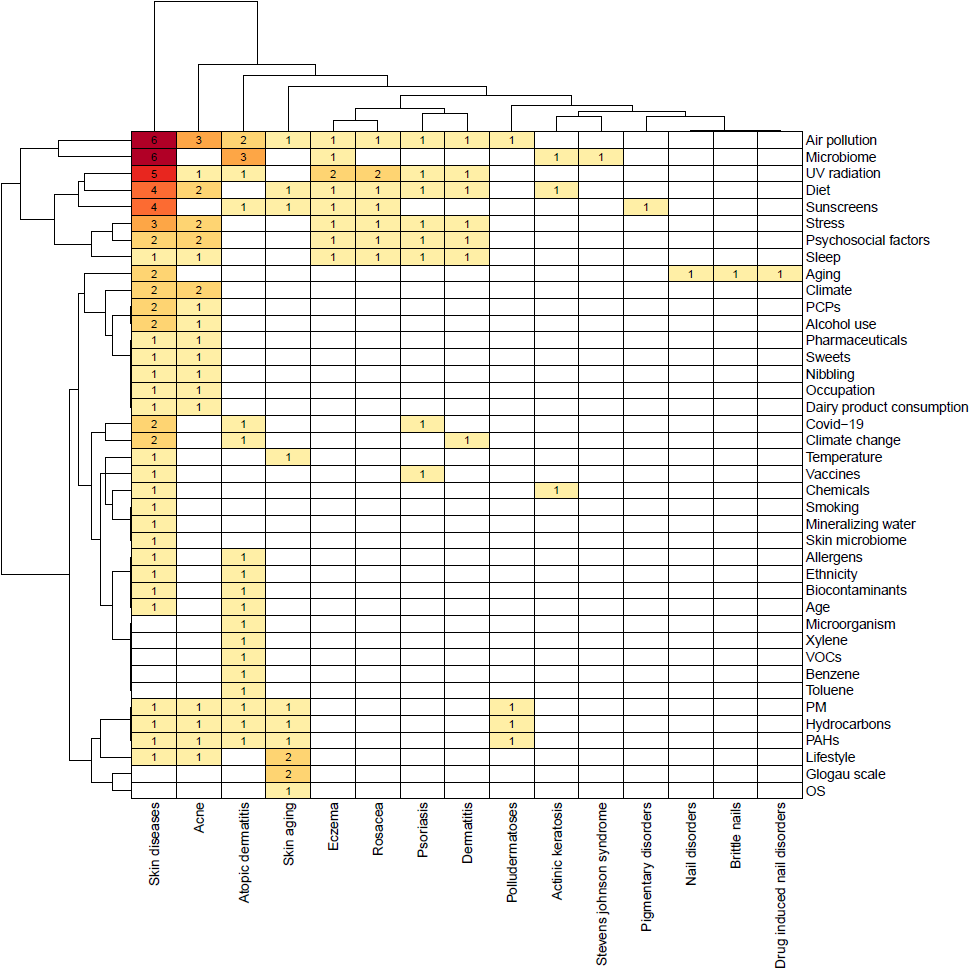


# **Fig.A.55: Heatmap of keyword co-occurrence between potential risk factor and skin disease keywords**

OS: oxidative stress, PAHs: polycyclic aromatic hydrocarbons, PCP: personal care product, PM: particulate matter, UV: ultraviolet. The number in each cell corresponds to the number of co-occurrences.


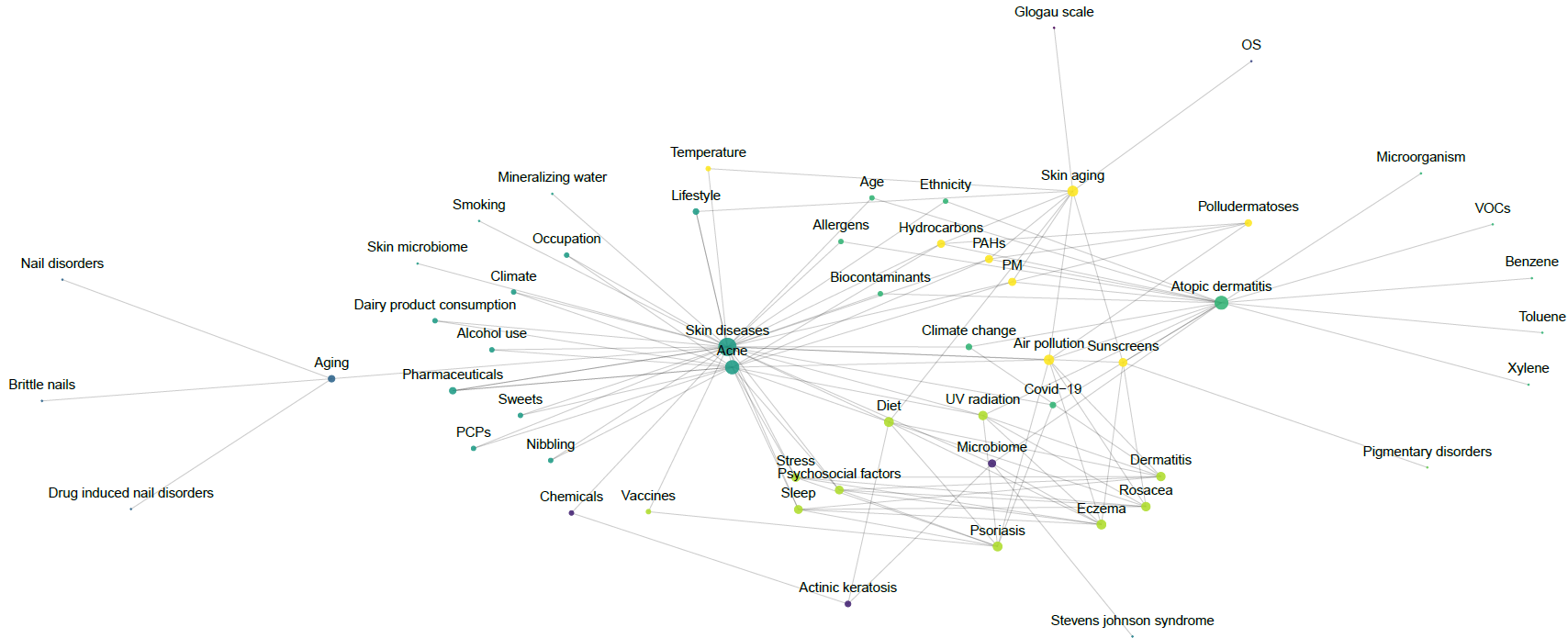


# **Fig.A.56: Keyword co-occurrence network between potential risk factor and skin disease keywords**

OS: oxidative stress, PAHs: polycyclic aromatic hydrocarbons, PCP: personal care product, PM: particulate matter, UV: ultraviolet.


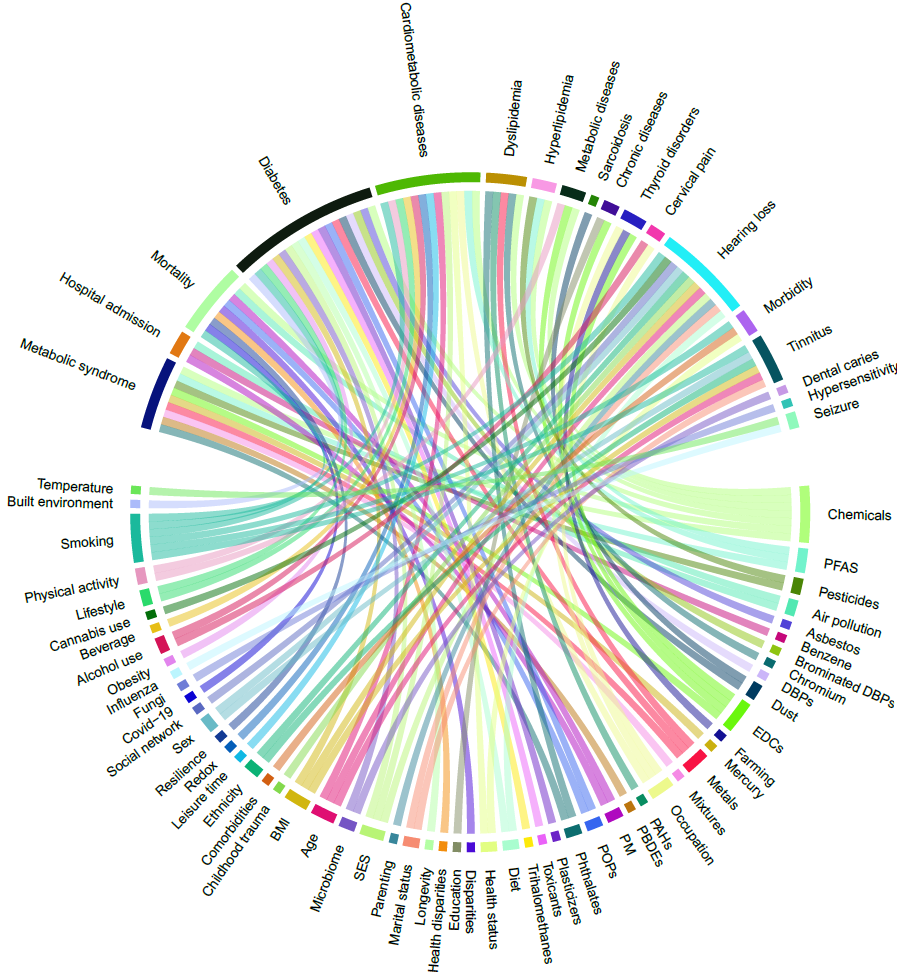


# **Fig.A.57: Chord diagram of keyword co-occurrence between potential risk factor and several health event keywords**

BMI: body mass index, EDCs: endocrine-disrupting chemicals, DBP: disinfection by-product, PAHs: polycyclic aromatic hydrocarbons, PBDEs: polybrominated diphenyl ethers, PFAS: per- and polyfluoroalkyl substances, PM: particulate matter, POPs: persistent organic pollutants, SES: socio-economic status.


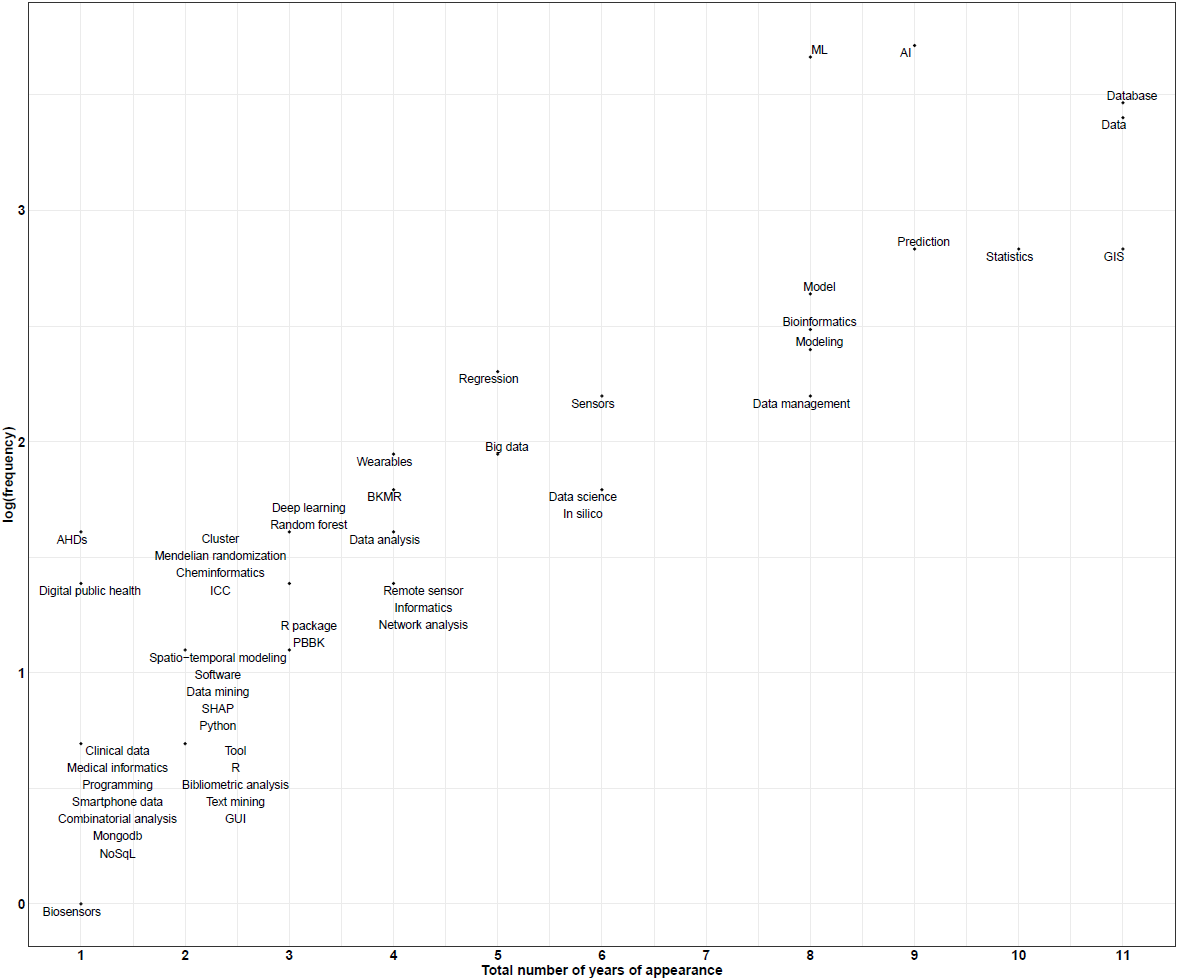


# **Fig.A.58: Top 10 of the most frequent keyword by periods of time**

AHD: administrative health database, AI: artificial intelligence, BKMR: Bayesian kernel machine regression, GIS: geographic information system, GPS: global positioning system, ICC: intra-class correlation, ML: machine learning, NoSqL: not only SQL, PBBK: physiologically-based kinetic, SHAP: SHAPley Additive exPlanations.


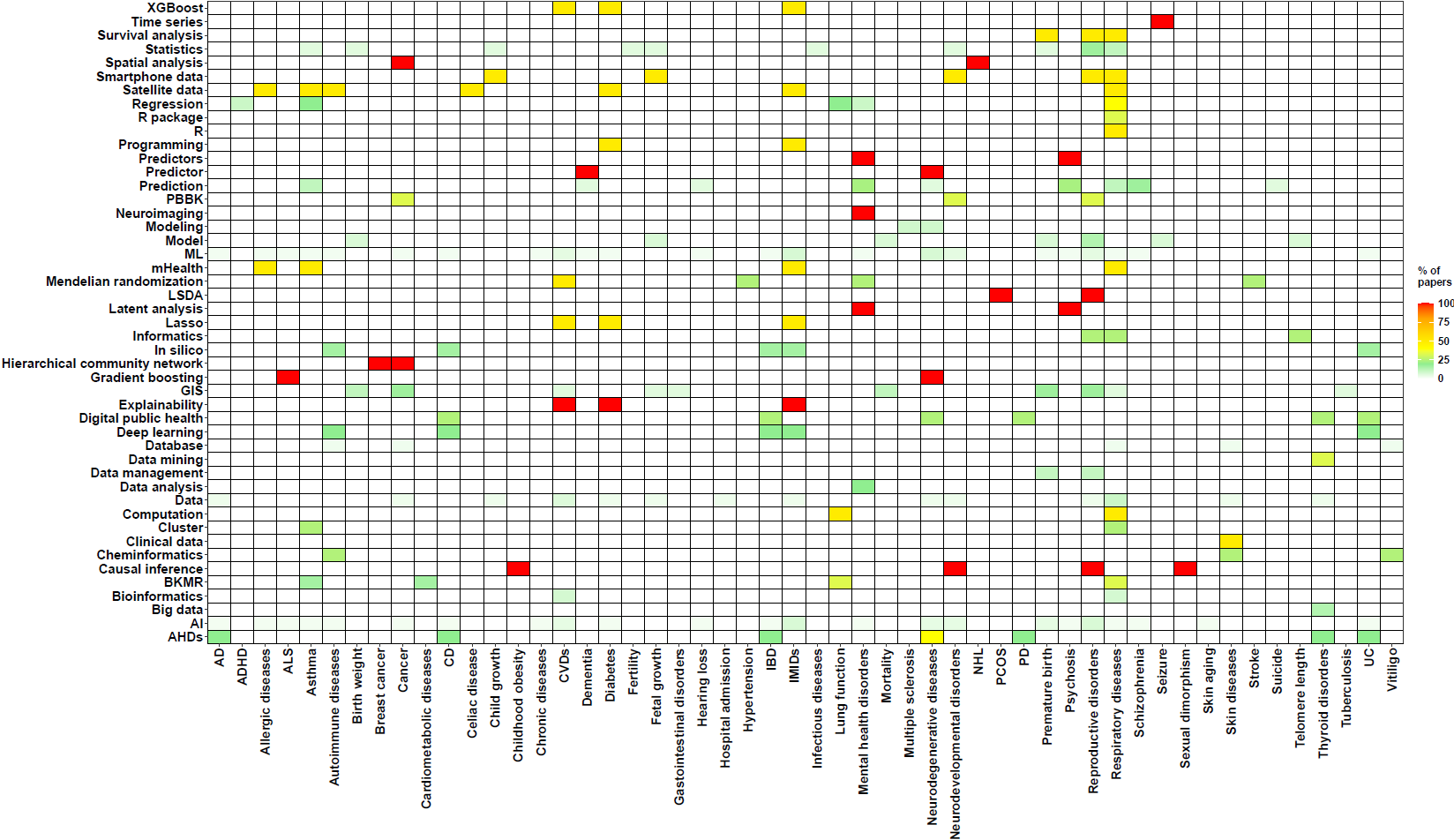


# **Fig.A.59: Most frequent health outcome-related keywords that co-occurred with digital tool-related keywords**

AHD: administrative health database, AD: Alzheimer’s disease, AI: artificial intelligence, ALS: amyotrophic lateral sclerosis, BKMR: Bayesian kernel machine regression, CD: Crohn’s disease, CVDs: cardiovascular diseases, IBDs: inflammatory bowel diseases, IMIDs: immune-mediated inflammatory diseases, GIS: geographic information system, LSDA: least squares discriminant analysis, ML: machine learning, NHL: non-Hodgkin lymphoma, PBBK: physiologically-based kinetic, PCOS: polycystic ovary syndrome, PD: Parkinson’s disease, UC: ulcerative colitis, XGBoost: extreme gradient boosting.


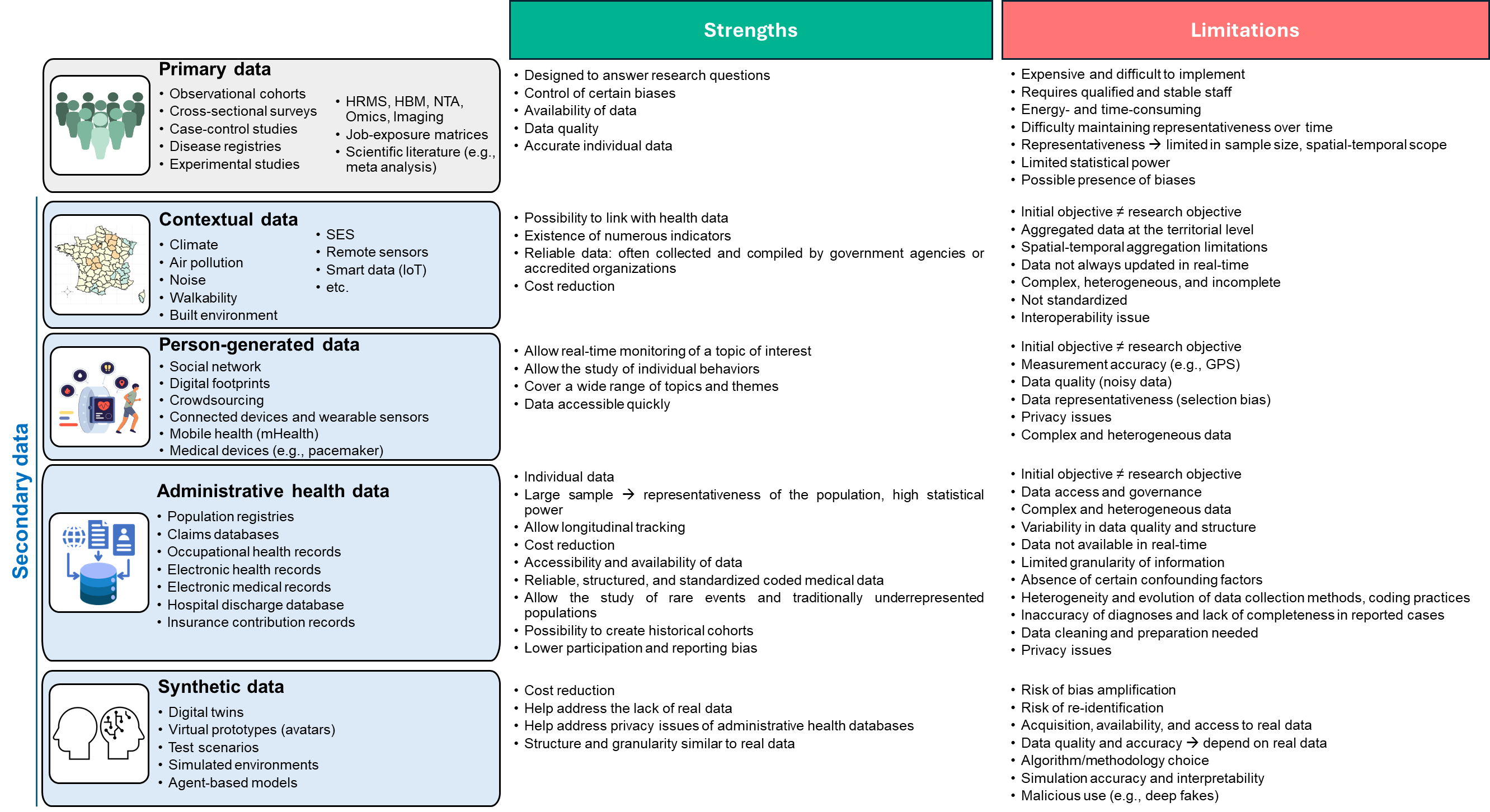


# **Fig.A.60: Strengths and limitations of exposome data**

AHDs: administrative health databases, HBM: human biomonitoring, HRMS: high-resolution mass spectrometry, IoT: Internet of Things, NTA: non-targeted analysis, SES: socio-economic status.

Primary data are specifically collected and tailored for a particular public health research purpose, and can be used either once or multiple times to achieve the same objective. They form the foundation of traditional public health policy and decision-making. In contrast, secondary data are not originally gathered for public health purposes, but they can complement primary data to enrich analysis and insights.

# **Table A.1**: Criteria used for the publication selection

| **Question** | **Description** | **Answer** | |
| --- | --- | --- | --- |
|  |  | no | yes/can’t tell |
| **Stage 1: Publication language** | | | |
| *Q_11_* | Is the publication written in English or French? | 0 | 1 |
| *S_1_ = Q_11_; Publication eligible for stage 2 if S_1_ > 0* | | | |
| **Stage 2: Screening publication title** | | | |
| *Q_21_* | Does the title mention terms related to exposome? | 0 | 1 |
| *Q_22_* | Does the title mention terms related to humans? | 0 | 1 |
| *S_2_ = Q_21_ × Q_22_; Publication eligible for stage 3 if S_2_ = 1* | | | |
| **Stage 3: Screening publication abstract** | | | |
| *Q_31_* | Does the abstract describe an analysis related to exposome? | 0 | 1 |
| *Q_32_* | Does the abstract describe an analysis conducted/related to humans? | 0 | 1 |
| *S_3_ = Q_31_ × Q_32_; Publication eligible for stage 4 if S_3_ = 1* | | | |
| ***Score = S_1_ × S_2_ × S_3_; Publication selected for review/analysis if score ≥ 1*** | | | |

*Note*: Q: question, S: score.

The final records were copied in Bibtex file format and transferred into the R software for bibliometric data processing.

# **Table A.2**: List of all publications included

Please refer to the MS Excel file entitled “Table A.2”.

# **Table A.3**: Main characteristics of the included publications

| **Description** | **Results** |
| --- | --- |
| Timespan | 01/01/2005 to 12/31/2024 |
| Sources (journals, books, etc.) | 424 |
| Number of publications | 931 |
| Annual growth rate % | 31.7 |
| Publication average age | 3.48 |
| Total number of citations | 26623 |
| Average citations per publication | 28.6 |
| Average citations per year per publication | 4.04 |
| Number of references | 56641 |
| Number of keywords plus (ID) | 2401 |
| Number of author's keywords (DE) | 1228 |
| Number of authors | 4529 |
| Number of author appearances | 7815 |
| Number of authors of single-authored publications | 45 (0.99%) |
| Number of single-authored publications | 34 (3.65%) |
| Number of publications per author | 0.21 |
| Number of co-authors per publication | 8.39 |
| International co-authorships (%) | 44.6 |
| Number of primary authorships for females | 471 (50.5%) |
| Number of primary authorships for males | 461 (49.5%) |
| Number of senior authorships for females* | 318 (35.5%) |
| Number of senior authorships for males* | 579 (64.5%) |
| Number of citations for female first authors | 10967 (41.2%) |
| Number of citations for female senior authors* | 6294 (23.6%) |
| Number of citations for male first authors | 10958 (41.2%) |
| Number of citations for male senior authors* | 15631 (58.7%) |

*Note*: Keywords plus is the total number of keywords that frequently appear in the title of a publication.

*excluding single-authored publications

# **Table A.4**: The most productive countries in the human exposome research field

| **Country** | **Continent** | **Publication**  **start** | **Publication**  **end** | **Number of**  **publications**  **(%)** | **Total**  **citation** | **Local**  **citation**  **(%)** | **Average**  **citation**  **per year** | **Mean**  **AGR** | **Fractionalized frequency**  **(%)** | **h-index** | **g-index** | **m-index** | **Scientific**  **production**  **per GDP** | **Scientific**  **production**  **per population** | **Scientific**  **production per**  **researchers in R&D**  **(per million people)** |
| --- | --- | --- | --- | --- | --- | --- | --- | --- | --- | --- | --- | --- | --- | --- | --- |
| USA | North America | 2010 | 2024 | 430 (46.2) | 15049 | 1562 (10.4) | 1003.3 | 51.2 | 284 | 64 | 105 | 4 | 1.7e-11 | 1.3e-06 | 0.09 |
| France | Europe | 2010 | 2024 | 199 (21.4) | 6937 | 1142 (16.5) | 462.5 | 41 | 86.5 | 42 | 78 | 2.62 | 7.2e-11 | 2.9e-06 | 0.04 |
| UK | Europe | 2005 | 2024 | 175 (18.8) | 7133 | 763 (10.7) | 356.7 | 33.9 | 53.1 | 42 | 80 | 2 | 5.7e-11 | 2.6e-06 | 0.04 |
| Spain | Europe | 2012 | 2024 | 160 (17.2) | 4822 | 711 (14.7) | 370.9 | 63 | 58.7 | 41 | 65 | 2.93 | 1.1e-10 | 3.4e-06 | 0.05 |
| The Netherlands | Europe | 2012 | 2024 | 129 (13.9) | 4759 | 657 (13.8) | 366.1 | 24.7 | 40.8 | 37 | 67 | 2.64 | 1.3e-10 | 7.3e-06 | 0.02 |
| Italy | Europe | 2013 | 2024 | 99 (10.6) | 3076 | 256 (8.33) | 256.3 | 53.2 | 46.7 | 27 | 54 | 2.08 | 4.9e-11 | 1.7e-06 | 0.04 |
| China | Asia | 2013 | 2024 | 88 (9.45) | 1148 | 75 (6.53) | 95.7 | 28.2 | 58.2 | 20 | 32 | 1.54 | 4.9e-12 | 6.2e-08 | 0.06 |
| Germany | Europe | 2012 | 2024 | 78 (8.38) | 2111 | 201 (9.52) | 162.4 | 11.4 | 33.2 | 24 | 44 | 1.71 | 1.9e-11 | 9.3e-07 | 0.01 |
| Greece | Europe | 2012 | 2024 | 77 (8.27) | 2989 | 480 (16.1) | 229.9 | 44.3 | 17.9 | 30 | 53 | 2.14 | 3.5e-10 | 7.3e-06 | 0.02 |
| Norway | Europe | 2014 | 2024 | 64 (6.87) | 2161 | 416 (19.3) | 196.5 | 0.45 | 9.07 | 25 | 46 | 2.08 | 1.1e-10 | 1.2e-05 | 9.6e-03 |
| Canada | North America | 2014 | 2024 | 52 (5.59) | 1780 | 131 (7.36) | 161.8 | 67.2 | 28.9 | 22 | 42 | 1.83 | 2.4e-11 | 1.3e-06 | 0.01 |
| Lithuania | Europe | 2014 | 2024 | 47 (5.05) | 2013 | 386 (19.2) | 183.0 | -4.81 | 6.37 | 24 | 44 | 2 | 6.7e-10 | 1.7e-05 | 0.01 |
| Sweden | Europe | 2012 | 2024 | 45 (4.83) | 733 | 74 (10.1) | 56.4 | 39.6 | 15.2 | 15 | 26 | 1.07 | 7.7e-11 | 4.3e-06 | 5.7e-03 |
| Australia | Oceania | 2013 | 2024 | 44 (4.73) | 1041 | 80 (7.68) | 86.8 | 11.9 | 14.7 | 18 | 31 | 1.38 | 2.6e-11 | 1.7e-06 | 9.7e-03 |
| Belgium | Europe | 2015 | 2024 | 43 (4.62) | 1105 | 132 (11.9) | 110.5 | 52.1 | 12.6 | 16 | 33 | 1.45 | 7.4e-11 | 3.7e-06 | 7.5e-03 |
| Switzerland | Europe | 2011 | 2024 | 34 (3.65) | 1048 | 57 (5.44) | 74.9 | -12 | 11.1 | 15 | 32 | 1 | 4.2e-11 | 3.9e-06 | 6.1e-03 |
| Austria | Europe | 2013 | 2024 | 33 (3.54) | 919 | 121 (13.2) | 76.6 | 11.9 | 15 | 17 | 30 | 1.31 | 7e-11 | 3.7e-06 | 5.6e-03 |
| Denmark | Europe | 2015 | 2024 | 25 (2.69) | 418 | 58 (13.9) | 41.8 | -15.7 | 6.5 | 11 | 20 | 1 | 6.3e-11 | 4.2e-06 | 3.2e-03 |
| Finland | Europe | 2018 | 2024 | 19 (2.04) | 247 | 48 (19.4) | 35.3 | 15.7 | 6.06 | 8 | 15 | 1 | 6.8e-11 | 3.4e-06 | 2.4e-03 |
| Singapore | Asia | 2017 | 2023 | 19 (2.04) | 457 | 56 (12.3) | 65.3 | -13.1 | 7.52 | 10 | 19 | 1.11 | 4.1e-11 | 3.4e-06 | 2.6e-03 |
| Brazil | South America | 2017 | 2024 | 18 (1.93) | 441 | 4 (0.907) | 55.1 | 19.6 | 8.33 | 11 | 18 | 1.22 | 9.4e-12 | 8.4e-08 | 0.02 |
| India | Asia | 2020 | 2024 | 15 (1.61) | 249 | 12 (4.82) | 49.8 | 26.7 | 11.1 | 6 | 15 | 1 | 4.4e-12 | 1.1e-08 | 0.06 |
| Ireland | Europe | 2017 | 2024 | 14 (1.5) | 281 | 18 (6.41) | 35.1 | 2.08 | 5.41 | 7 | 14 | 0.778 | 2.7e-11 | 2.8e-06 | 2.6e-03 |
| South Korea | Asia | 2016 | 2024 | 13 (1.4) | 268 | 5 (1.87) | 29.8 | -7.41 | 9.25 | 6 | 13 | 0.6 | 7.2e-12 | 2.5e-07 | 1.5e-03 |
| Luxembourg | Europe | 2020 | 2024 | 13 (1.4) | 584 | 139 (23.8) | 116.8 | 31.7 | 6.13 | 6 | 13 | 1 | 1.6e-10 | 2e-05 | 2.4e-03 |
| Poland | Europe | 2017 | 2024 | 13 (1.4) | 441 | 42 (9.52) | 55.1 | 2.08 | 5.41 | 8 | 13 | 0.889 | 1.9e-11 | 3.5e-07 | 4e-03 |
| Cyprus | Europe | 2018 | 2023 | 12 (1.29) | 151 | 50 (33.1) | 25.2 | -25 | 7.24 | 6 | 12 | 0.75 | 4.2e-10 | 9.6e-06 | 7.1e-03 |
| Czech Republic | Europe | 2020 | 2024 | 12 (1.29) | 182 | 57 (31.3) | 36.4 | 86.7 | 4.25 | 6 | 12 | 1 | 4.1e-11 | 1.1e-06 | 2.9e-03 |
| Japan | Asia | 2017 | 2024 | 12 (1.29) | 365 | 6 (1.64) | 45.6 | 30 | 6.59 | 8 | 12 | 0.889 | 2.8e-12 | 9.6e-08 | 2.2e-03 |
| Slovenia | Europe | 2018 | 2024 | 10 (1.07) | 151 | 34 (22.5) | 21.6 | -2.38 | 3.11 | 7 | 10 | 0.875 | 1.6e-10 | 4.7e-06 | 2e-03 |
| South Africa | Africa | 2019 | 2024 | 10 (1.07) | 93 | 6 (6.45) | 15.5 | 58.3 | 3.43 | 5 | 9 | 0.714 | 2.5e-11 | 1.7e-07 | 0.02 |
| Turkey | Europe | 2019 | 2022 | 10 (1.07) | 457 | 18 (3.94) | 114.3 | 47.5 | 2.31 | 9 | 10 | 1.29 | 1.1e-11 | 1.2e-07 | 5.6e-03 |
| Mexico | North America | 2018 | 2024 | 8 (0.86) | 137 | 4 (2.92) | 19.6 | -39.3 | 3.9 | 5 | 8 | 0.625 | 5.7e-12 | 6.3e-08 | 0.02 |
| Portugal | Europe | 2017 | 2024 | 8 (0.86) | 183 | 3 (1.64) | 22.9 | -25 | 4.53 | 5 | 8 | 0.556 | 3.2e-11 | 7.7e-07 | 1.5e-03 |
| Romania | Europe | 2018 | 2024 | 7 (0.75) | 245 | 21 (8.57) | 35.0 | 0 | 2.72 | 4 | 7 | 0.5 | 2.3e-11 | 3.7e-07 | 5.3e-03 |
| Chile | South America | 2022 | 2024 | 6 (0.64) | 49 | 0 (0) | 16.3 | 11.1 | 2.61 | 4 | 6 | 1 | 2e-11 | 3.1e-07 | 0.01 |
| Russian federation | Europe-Asia | 2018 | 2023 | 6 (0.64) | 141 | 3 (2.13) | 23.5 | -8.33 | 2.96 | 4 | 6 | 0.5 | 2.7e-12 | 4.2e-08 | 1.6e-03 |
| North Macedonia | Europe | 2021 | 2023 | 5 (0.54) | 31 | 9 (29) | 10.3 | 33.3 | 0.671 | 3 | 5 | 0.6 | 3.7e-10 | 2.4e-06 | 5.8e-03 |
| Serbia | Europe | 2019 | 2023 | 5 (0.54) | 196 | 9 (4.59) | 39.2 | -30 | 1.05 | 4 | 5 | 0.571 | 7.9e-11 | 7.4e-07 | 2.3e-03 |
| Bulgaria | Europe | 2021 | 2023 | 4 (0.43) | 18 | 4 (22.2) | 6.00 | 33.3 | 1.49 | 2 | 4 | 0.4 | 4.5e-11 | 6.2e-07 | 1.7e-03 |
| Colombia | South America | 2018 | 2024 | 4 (0.43) | 39 | 1 (2.56) | 5.57 | 0 | 2.17 | 2 | 4 | 0.25 | 1.2e-11 | 7.7e-08 | 0.04 |
| Croatia | Europe | 2018 | 2024 | 4 (0.43) | 91 | 20 (22) | 13.0 | -28.6 | 0.586 | 3 | 4 | 0.375 | 5.6e-11 | 1e-06 | 1.8e-03 |
| Israel | Asia | 2013 | 2024 | 4 (0.43) | 49 | 6 (12.2) | 4.08 | -16.7 | 1.58 | 2 | 4 | 0.154 | 7.7e-12 | 4.2e-07 | no data |
| Egypt | Africa | 2021 | 2023 | 3 (0.32) | 16 | 2 (12.5) | 5.33 | 0 | 2.2 | 2 | 3 | 0.4 | 7.1e-12 | 2.8e-08 | 3.6e-03 |
| Hungary | Europe | 2020 | 2023 | 3 (0.32) | 508 | 134 (26.4) | 127.0 | -25 | 0.708 | 3 | 3 | 0.5 | 1.7e-11 | 3.1e-07 | 6.9e-04 |
| Nigeria | Africa | 2022 | 2023 | 3 (0.32) | 30 | 11 (36.7) | 15.0 | -25 | 1.5 | 3 | 3 | 0.75 | 6.3e-12 | 1.4e-08 | 0.08 |
| Thailand | Asia | 2023 | 2024 | 3 (0.32) | 4 | 0 (0) | 2.00 | 50 | 0.56 | 1 | 1 | 0.333 | 6.1e-12 | 4.2e-08 | 1.7e-03 |
| Burkina Faso | Africa | 2023 | 2024 | 2 (0.22) | 3 | 1 (33.3) | 1.50 | 0 | 0.5 | 1 | 1 | 0.333 | 1.1e-10 | 8.8e-08 | 0.04 |
| Iran | Asia | 2024 | 2024 | 2 (0.22) | 2 | 0 (0) | 2.00 | 0 | 0.75 | 1 | 1 | 0.5 | 5.6e-12 | 2.3e-08 | 1.2e-03 |
| Kenya | Africa | 2016 | 2019 | 2 (0.22) | 25 | 1 (4) | 6.25 | -25 | 1 | 2 | 2 | 0.2 | 1.8e-11 | 3.7e-08 | 9e-03 |
| Malaysia | Asia | 2024 | 2024 | 2 (0.22) | 1 | 0 (0) | 1.00 | 0 | 1.17 | 1 | 1 | 0.5 | 4.9e-12 | 5.9e-08 | 8.4e-04 |
| New Zealand | Oceania | 2018 | 2021 | 2 (0.22) | 24 | 1 (4.17) | 6.00 | -25 | 1.33 | 2 | 2 | 0.25 | 8.1e-12 | 3.9e-07 | 3.4e-04 |
| Suriname | South America | 2022 | 2024 | 2 (0.22) | 7 | 0 (0) | 2.33 | -33.3 | 0.833 | 2 | 2 | 0.5 | 5.5e-10 | 3.2e-06 | no data |
| United Arab Emirates | Asia | 2019 | 2019 | 2 (0.22) | 6 | 0 (0) | 6.00 | 0 | 0.583 | 1 | 2 | 0.143 | 3.9e-12 | 2.1e-07 | 8.2e-04 |
| Uruguay | South America | 2020 | 2022 | 2 (0.22) | 45 | 3 (6.67) | 15.0 | -33.3 | 0.643 | 2 | 2 | 0.333 | 2.8e-11 | 5.8e-07 | 2.6e-03 |
| Costa Rica | South America | 2020 | 2020 | 1 (0.11) | 14 | 0 (0) | 14.0 | 0 | 0.333 | 1 | 1 | 0.167 | 1.5e-11 | 1.9e-07 | 1.8e-03 |
| Cuba | North America | 2019 | 2019 | 1 (0.11) | 43 | 0 (0) | 43.0 | 0 | 0.125 | 1 | 1 | 0.143 | 9.3e-12 | 8.9e-08 | no data |
| Estonia | Europe | 2021 | 2021 | 1 (0.11) | 24 | 9 (37.5) | 24.0 | 0 | 0.111 | 1 | 1 | 0.2 | 2.6e-11 | 7.4e-07 | 2.6e-04 |
| Ethiopia | Africa | 2024 | 2024 | 1 (0.11) | 1 | 0 (0) | 1.00 | 0 | 0.167 | 1 | 1 | 0.5 | 7.9e-12 | 8.1e-09 | 0.01 |
| Iceland | Europe | 2022 | 2022 | 1 (0.11) | 12 | 4 (33.3) | 12.0 | 0 | 0.25 | 1 | 1 | 0.25 | 3.6e-11 | 2.6e-06 | 1.3e-04 |
| Iraq | Asia | 2022 | 2022 | 1 (0.11) | 10 | 5 (50) | 10.0 | 0 | 0.5 | 1 | 1 | 0.25 | 3.8e-12 | 2.3e-08 | 2.3e-03 |
| Liberia | Africa | 2024 | 2024 | 1 (0.11) | 1 | 0 (0) | 1.00 | 0 | 0.5 | 1 | 1 | 0.5 | 2.5e-10 | 1.9e-07 | no data |
| Malawi | Africa | 2021 | 2021 | 1 (0.11) | 8 | 1 (12.5) | 8.00 | 0 | 0.25 | 1 | 1 | 0.2 | 7.6e-11 | 4.9e-08 | 0.02 |
| Malta | Europe | 2020 | 2020 | 1 (0.11) | 20 | 1 (5) | 20.0 | 0 | 0.333 | 1 | 1 | 0.167 | 5.6e-11 | 1.9e-06 | 4.4e-04 |
| Mauritius | Africa | 2022 | 2022 | 1 (0.11) | 4 | 0 (0) | 4.00 | 0 | 0.333 | 1 | 1 | 0.25 | 7.8e-11 | 7.9e-07 | 1.8e-03 |
| Mongolia | Asia | 2023 | 2023 | 1 (0.11) | 10 | 0 (0) | 10.0 | 0 | 0.333 | 1 | 1 | 0.333 | 6e-11 | 2.9e-07 | 3e-03 |
| Peru | South America | 2021 | 2021 | 1 (0.11) | 28 | 1 (3.57) | 28.0 | 0 | 0.2 | 1 | 1 | 0.2 | 4.1e-12 | 2.9e-08 | no data |
| Qatar | Asia | 2018 | 2018 | 1 (0.11) | 151 | 75 (49.7) | 151.0 | 0 | 0.111 | 1 | 1 | 0.125 | 4.2e-12 | 3.7e-07 | 1.7e-03 |
| Saudi Arabia | Asia | 2014 | 2014 | 1 (0.11) | 256 | 0 (0) | 256.0 | 0 | 0.111 | 1 | 1 | 0.0833 | 9e-13 | 2.8e-08 | 2.2e-03 |
| Syria | Asia | 2023 | 2023 | 1 (0.11) | 2 | 0 (0) | 2.00 | 0 | 0.5 | 1 | 1 | 0.333 | 9e-11 | 4.5e-08 | 0.01 |
| Uganda | Africa | 2022 | 2022 | 1 (0.11) | 23 | 0 (0) | 23.0 | 0 | 0.143 | 1 | 1 | 0.25 | 2.2e-11 | 2.1e-08 | 0.03 |

*Note*: AGR: annual growth rate, GDP: gross domestic product. Please refer to the beginning of the supplemental materials for the definition of the bibliometric indices.

# **Table A.5**: Top 30 of the most cited publications in the human exposome research field

| **Publication** | **Publication year** | **Journal** | **DOI** | **Total**  **citation** | **Local**  **citation**  **(%)** | **Average**  **citation**  **per year** | **Main theme** |
| --- | --- | --- | --- | --- | --- | --- | --- |
| Wild, 2005 [6] | 2005 | Cancer Epidemiol Biomarkers Prev | 10.1158/1055-9965.EPI-05-0456 | 1457 | 0 (0) | 72.8 | Cancer, omics |
| Wild, 2012 [7] | 2012 | Int J Epidemiol | 10.1093/ije/dyr236 | 786 | 262 (33.3) | 60.5 | Epidemiology |
| Nikolich-Zugich, 2018 [8] | 2018 | Nat Immunol | 10.1038/s41590-017-0006-x | 678 | 0 (0) | 96.9 | Aging, immune system |
| Vermeulen et al. 2020 [9] | 2020 | Science | 10.1126/science.aay3164 | 486 | 127 (26.1) | 97.2 | Chemicals, HRMS |
| Krutmann et al. 2017 [10] | 2017 | J Dermatol Sci | 10.1016/j.jdermsci.2016.09.015 | 447 | 30 (6.71) | 55.9 | Skin aging |
| Rappaport, 2011 [11] | 2011 | J Expo Sci Environ Epidemiol | 10.1038/jes.2010.50 | 302 | 0 (0) | 21.6 | Exposure science |
| Gacesa et al. 2022 [12] | 2022 | Nature | 10.1038/s41586-022-04567-7 | 283 | 2 (0.71) | 94.3 | Gut microbiome |
| Davis et al. 2023 [13] | 2022 | Nucleic Acids Res | 10.1093/nar/gkac833 | 267 | 0 (0) | 89.0 | Toxicogenomics |
| Go et al. 2015 [14] | 2015 | Free Radic Biol Med | 10.1016/j.freeradbiomed.2015.03.022 | 264 | 0 (0) | 26.4 | Cysteine proteome |
| Miller and O'Callaghan, 2015 [15] | 2015 | Metabolism | 10.1016/j.metabol.2014.10.030 | 262 | 0 (0) | 26.2 | Parkinson's disease, biomarkers |
| Rappaport et al. 2014 [16] | 2014 | Environ Health Perspect | 10.1289/ehp.1308015 | 257 | 0 (0) | 23.4 | Blood exposome |
| Vrijheid et al. 2014 [17] | 2014 | Environ Health Perspect | 10.1289/ehp.1307204 | 256 | 0 (0) | 23.3 | Early-life, omics |
| Vrijens et al. 2015 [18] | 2015 | Environ Health Perspect | 10.1289/ehp.1408459 | 245 | 0 (0) | 24.5 | MicroRNAs |
| Burbank et al. 2017 [19] | 2017 | J Allergy Clin Immunol | 10.1016/j.jaci.2017.05.010 | 204 | 0 (0) | 25.5 | Early-life, allergy, asthma |
| Jones et al. 2012 [20] | 2012 | Annu Rev Nutr | 10.1146/annurev-nutr-072610-145159 | 200 | 14 (7) | 15.4 | Metabolomics, diet |
| Vrijheid, 2014 [21] | 2014 | Thorax | 10.1136/thoraxjnl-2013-204949 | 194 | 56 (28.9) | 17.6 | Epidemiology |
| Wishart et al. 2015 [22] | 2015 | Nucleic Acids Res | 10.1093/nar/gku1004 | 188 | 28 (14.9) | 18.8 | Chemicals, toxicology |
| Vineis et al. 2017 [23] | 2017 | Int J Hyg Environ Health | 10.1016/j.ijheh.2016.08.001 | 184 | 0 (0) | 23.0 | Omics, EWAS |
| Ugai et al. 2022 [24] | 2022 | Nat Rev Clin Oncol | 10.1038/s41571-022-00672-8 | 184 | 2 (1.09) | 61.3 | Early-life, cancer |
| Celebi Sozener et al. 2022 [25] | 2022 | Allergy | 10.1111/all.15240 | 183 | 4 (2.19) | 61.0 | Epithelial barrier, allergy |
| Kumar et al. 2020 [26] | 2020 | Front Public Health | 10.3389/fpubh.2020.553850 | 172 | 1 (0.58) | 34.4 | EDCs, non-communicable diseases |
| Uppal et al. 2016 [27] | 2016 | Chem Res Toxicol | 10.1021/acs.chemrestox.6b00179 | 170 | 25 (14.7) | 18.9 | Metabolomics |
| Go et al. 2015 [28] | 2015 | Toxicol Sci | 10.1093/toxsci/kfv198 | 169 | 27 (16.0) | 16.9 | Metabolomics, HRMS |
| Rappaport, 2016 [29] | 2016 | PLoS One | 10.1371/journal.pone.0154387 | 157 | 41 (26.1) | 17.4 | GWAS, EWAS |
| Wild et al. 2013 [30] | 2013 | Environ Mol Mutagen | 10.1002/em.21777 | 156 | 32 (20.5) | 13.0 | Omics, cancer |
| Maitre et al. 2018 [31] | 2018 | BMJ Open | 10.1136/bmjopen-2017-021311 | 151 | 75 (49.7) | 21.6 | Early-life, omics, allergy, asthma |
| Lochhead et al. 2015 [32] | 2015 | Mod Pathol | 10.1038/modpathol.2014.81 | 148 | 0 (0) | 14.8 | Etiologic field effect, cancer |
| Janssen et al. 2015 [33] | 2015 | Epigenetics | 10.1080/15592294.2015.1048412 | 147 | 0 (0) | 14.7 | Early-life, DNA methylation |
| Dennis et al. 2017 [34] | 2017 | Environ Health Perspect | 10.1289/EHP474 | 146 | 0 (0) | 18.2 | HBM |
| Jones, 2015 [35] | 2015 | Redox Biol | 10.1016/j.redox.2015.03.004 | 144 | 4 (2.78) | 14.4 | Redox, oxidative stress |

*Note*: DNA: deoxyribonucleic acid, DOI: digital object identifier, EDC : endocrine disrupting chemical, EWAS : exposome-wide association study, GWAS: genome-wide association study, HBM: human biomonitoring, HRMS: high-resolution mass spectrometry, RNA: ribonucleic acid. Please refer to the beginning of the supplemental materials for the definition of the bibliometric indices.

# **Table A.6**: Top 20 of the most active journals in the human exposome research field

| **Journal** | **Publication**  **start** | **Number of**  **publications (%)** | **Total**  **citation** | **h-index** | **Average citation**  **per year** | **Mean AGR** | **g-index** | **m-index** | **Impact factor**  **(2024)** | **Rank** |
| --- | --- | --- | --- | --- | --- | --- | --- | --- | --- | --- |
| Environment International | 2015 | 58 (6.23) | 1636 | 23 | 163.6 | 16.3 | 40 | 2.09 | 10.3 | Q1 |
| Environmental Research | 2017 | 35 (3.76) | 422 | 14 | 52.8 | 38.8 | 19 | 1.56 | 7.7 | Q1 |
| Environmental Health Perspectives | 2010 | 32 (3.44) | 1995 | 20 | 133.0 | 37.8 | 32 | 1.25 | 10.1 | Q1 |
| Environmental Science & Technology | 2015 | 32 (3.44) | 552 | 12 | 55.2 | 12.2 | 23 | 1.09 | 10.9 | Q1 |
| International Journal Of Environmental Research And Public Health | 2014 | 20 (2.15) | 555 | 12 | 50.5 | 13 | 20 | 1 | 4.61* | Q1* |
| Science Of The Total Environment | 2018 | 19 (2.04) | 231 | 9 | 33.0 | 30 | 15 | 1.13 | 8.2 | Q1 |
| Frontiers In Public Health | 2020 | 18 (1.93) | 278 | 6 | 55.6 | 28.7 | 16 | 1 | 3.0 | Q2 |
| Scientific Reports | 2017 | 15 (1.61) | 147 | 7 | 18.4 | 47.9 | 12 | 0.78 | 3.8 | Q1 |
| Journal Of Exposure Science And Environmental Epidemiology | 2011 | 11 (1.18) | 337 | 5 | 24.1 | -14.3 | 11 | 0.33 | 4.1 | Q1 |
| Molecular Aspects Of Medicine | 2022 | 10 (1.07) | 151 | 8 | 50.3 | 0 | 10 | 2 | 8.7 | Q1 |
| Environmental Epidemiology | 2021 | 10 (1.07) | 127 | 7 | 31.8 | 0 | 10 | 1.4 | 3.3 | Q1 |
| Allergy | 2019 | 9 (0.97) | 627 | 7 | 104.5 | -8.33 | 9 | 1 | 12.6 | Q1 |
| Analytical Chemistry | 2013 | 9 (0.97) | 271 | 6 | 22.6 | -16.7 | 9 | 0.46 | 6.8 | Q1 |
| Environmental Pollution | 2018 | 8 (0.86) | 198 | 5 | 28.3 | -21.4 | 8 | 0.63 | 7.6 | Q1 |
| International Journal Of Hygiene And Environmental Health | 2017 | 8 (0.86) | 328 | 5 | 41.0 | -37.5 | 8 | 0.56 | 4.5 | Q1 |
| International Journal Of Molecular Sciences | 2020 | 8 (0.86) | 80 | 4 | 16.0 | -10 | 8 | 0.67 | 4.9 | Q1 |
| Bmc Medicine | 2012 | 6 (0.64) | 249 | 5 | 19.2 | -25 | 6 | 0.36 | 7.1 | Q1 |
| Current Opinion In Pediatrics | 2016 | 6 (0.64) | 198 | 6 | 22.0 | 0 | 6 | 0.6 | 2.2 | Q2 |
| Environmental Science & Technology Letters | 2021 | 6 (0.64) | 95 | 3 | 23.8 | -37.5 | 6 | 0.6 | 8.9 | Q1 |
| International Journal Of Epidemiology | 2012 | 6 (0.64) | 851 | 3 | 65.5 | -7.69 | 6 | 0.21 | 6.4 | Q1 |
| Journal Of Occupational And Environmental Medicine | 2016 | 6 (0.64) | 111 | 5 | 12.3 | -25 | 6 | 0.50 | 2.3 | Q2 |
| Journal Of The European Academy Of Dermatology And Venereology | 2018 | 6 (0.64) | 270 | 6 | 38.6 | -33.3 | 6 | 0.75 | 8.5 | Q1 |
| Metabolites | 2020 | 6 (0.64) | 129 | 4 | 25.8 | -33.3 | 6 | 0.67 | 3.5 | Q2 |
| Nature Communications | 2021 | 6 (0.64) | 165 | 5 | 41.3 | 37.5 | 6 | 1 | 14.7 | Q1 |
| Nucleic Acids Research | 2015 | 6 (0.64) | 627 | 5 | 62.7 | -40 | 6 | 0.45 | 16.7 | Q1 |
| Plos One | 2015 | 6 (0.64) | 198 | 4 | 19.8 | -20 | 6 | 0.36 | 2.9 | Q1 |
| Tract-rends In Analytical Chemistry | 2019 | 6 (0.64) | 96 | 4 | 16.0 | -8.33 | 6 | 0.57 | 11.8 | Q1 |

*Note*: AGR: annual growth rate. Please refer to the beginning of the supplemental materials for the definition of the bibliometric indices.

*latest data available from 2021

# **Table A.7**: Top 30 of the most prolific authors in the human exposome research field

| **Author** | **Sex** | **Publication**  **period** | **Number of**  **publications (%)** | **Fractionalized**  **frequency (%)** | **DF first**  **author (%)** | **DF last**  **author (%)** | **Total**  **citation** | **Local**  **citation (%)** | **Average citation**  **per year** | **h-index** | **Mean AGR** | **g-index** | **m-index** | **Y-index** |
| --- | --- | --- | --- | --- | --- | --- | --- | --- | --- | --- | --- | --- | --- | --- |
| VRIJHEID M | Female | 2014-2024 | 70 (7.52) | 5.38 | 4.35 | 20.3 | 2855 | 544 (19.1) | 259.5 | 30 | 35.5 | 53 | 2.5 | 14.3 |
| SLAMA R | Male | 2014-2024 | 44 (4.73) | 3.52 | 0 | 20.5 | 2046 | 445 (21.7) | 186.0 | 24 | 70.7 | 44 | 2 | 9 |
| MAITRE L | Female | 2018-2024 | 43 (4.62) | 2.32 | 11.6 | 6.98 | 1452 | 383 (26.4) | 207.4 | 21 | 20.6 | 38 | 2.62 | 5.83 |
| CHATZI L | Female | 2014-2024 | 41 (4.4) | 1.81 | 0 | 12.2 | 1835 | 356 (19.4) | 166.8 | 23 | -2.84 | 41 | 1.92 | 5 |
| GRAZULEVICIENE R | Female | 2014-2024 | 40 (4.3) | 1.73 | 0 | 0 | 1616 | 321 (19.9) | 146.9 | 21 | 7.69 | 40 | 1.75 | 0 |
| WRIGHT J | Male | 2018-2024 | 40 (4.3) | 1.72 | 0 | 0 | 1497 | 320 (21.4) | 213.9 | 22 | 10.5 | 38 | 2.75 | 0 |
| BASAGANA X | Male | 2014-2024 | 39 (4.19) | 2.64 | 0 | 17.9 | 1877 | 402 (21.4) | 170.6 | 24 | 37.4 | 39 | 2 | 7 |
| CASAS M | Female | 2014-2024 | 37 (3.97) | 1.65 | 2.7 | 2.7 | 1592 | 288 (18.1) | 144.7 | 22 | -1.89 | 37 | 1.83 | 1.41 |
| NIEUWENHUIJSEN MJ | Male | 2014-2024 | 34 (3.65) | 2.14 | 5.88 | 8.82 | 1454 | 241 (16.6) | 132.2 | 20 | 6.97 | 34 | 1.67 | 3.61 |
| VAFEIADI M | Female | 2018-2024 | 32 (3.44) | 1.34 | 0 | 0 | 1358 | 301 (22.2) | 194.0 | 20 | 25.2 | 32 | 2.5 | 0 |
| MCEACHAN RRC | Female | 2014-2024 | 30 (3.22) | 1.27 | 0 | 0 | 1513 | 278 (18.4) | 137.5 | 20 | 2.9 | 30 | 1.67 | 0 |
| VERMEULEN RCH | Male | 2013-2024 | 30 (3.22) | 2.87 | 3.33 | 23.3 | 1455 | 227 (15.6) | 121.3 | 15 | 23.3 | 30 | 1.15 | 7.07 |
| URQUIZA J | Male | 2018-2024 | 29 (3.11) | 1.25 | 0 | 0 | 1246 | 259 (20.8) | 178.0 | 18 | 8.57 | 29 | 2.25 | 0 |
| ANDRUSAITYTE S | Female | 2018-2024 | 27 (2.9) | 1.09 | 0 | 0 | 1139 | 292 (25.6) | 162.7 | 16 | 9.29 | 27 | 2 | 0 |
| BUSTAMANTE M | Female | 2014-2024 | 26 (2.79) | 1.32 | 3.85 | 19.2 | 706 | 141 (20) | 64.2 | 11 | -12.9 | 26 | 0.917 | 5.1 |
| JONES DP | Male | 2012-2024 | 26 (2.79) | 6.93 | 12.5 | 62.5 | 1773 | 204 (11.5) | 136.4 | 17 | 8.21 | 26 | 1.21 | 15.3 |
| ROBINSON O | Male | 2014-2024 | 26 (2.79) | 1.56 | 11.5 | 0 | 1752 | 408 (23.3) | 159.3 | 21 | -18.9 | 26 | 1.75 | 3 |
| SIROUX V | Female | 2014-2024 | 25 (2.69) | 1.86 | 4 | 24 | 1391 | 256 (18.4) | 126.5 | 17 | 30.8 | 25 | 1.42 | 6.08 |
| THOMSEN C | Female | 2014-2024 | 25 (2.69) | 1.07 | 0 | 4 | 1436 | 316 (22) | 130.5 | 18 | -11.4 | 25 | 1.5 | 1 |
| GONZALEZ JR | Male | 2014-2024 | 24 (2.58) | 1.45 | 0 | 16.7 | 1280 | 257 (20.1) | 116.4 | 17 | 11.8 | 24 | 1.42 | 4 |
| HEUDE B | Female | 2018-2024 | 23 (2.47) | 0.934 | 0 | 0 | 769 | 195 (25.4) | 109.9 | 11 | 39.5 | 23 | 1.38 | 0 |
| WALKER DI | Male | 2015-2024 | 22 (2.36) | 2.64 | 13.6 | 0 | 921 | 160 (17.4) | 92.1 | 14 | 22.8 | 22 | 1.27 | 3 |
| GUTZKOW KB | Female | 2014-2024 | 21 (2.26) | 0.964 | 0 | 4.76 | 841 | 151 (18) | 76.5 | 13 | 5.3 | 21 | 1.08 | 1 |
| HAUG LS | Female | 2018-2024 | 19 (2.04) | 0.773 | 5.26 | 0 | 1169 | 290 (24.8) | 167.0 | 15 | 16.7 | 19 | 1.88 | 1 |
| SUNYER J | Male | 2014-2024 | 19 (2.04) | 1.06 | 0 | 5.26 | 1166 | 261 (22.4) | 106.0 | 15 | -13.6 | 19 | 1.25 | 1 |
| KEUN HC | Male | 2012-2024 | 18 (1.93) | 0.949 | 0 | 16.7 | 1060 | 206 (19.4) | 81.5 | 11 | -14.1 | 18 | 0.786 | 3 |
| WARTH B | Male | 2017-2024 | 18 (1.93) | 3.43 | 5.56 | 72.2 | 337 | 76 (22.6) | 42.1 | 10 | 14.6 | 18 | 1.11 | 13 |
| LEPEULE J | Female | 2018-2024 | 16 (1.72) | 0.843 | 0 | 0 | 389 | 32 (8.23) | 55.6 | 9 | 11.9 | 16 | 1.12 | 0 |
| SARIGIANNIS DA | Male | 2016-2023 | 16 (1.72) | 3.48 | 26.7 | 33.3 | 424 | 55 (13) | 47.1 | 11 | 24 | 16 | 1.1 | 6.4 |
| VINEIS P | Male | 2013-2024 | 16 (1.72) | 3.6 | 21.4 | 0 | 807 | 99 (12.3) | 67.3 | 12 | 14.2 | 16 | 0.923 | 3 |

*Note*: AGR: annual growth rate, DF: dominance factor. Please refer to the beginning of the supplemental materials for the definition of the bibliometric indices.

# **Table A.8**: Number of publications and citations over time, stratified by authorship and sex

| **Year** | **Number of publications by primary authorship** | | | | **Number of publications by senior authorship** | | | | | **Number of citations by primary authorship** | | | | | **Number of citations by senior authorship** | | | | |
| --- | --- | --- | --- | --- | --- | --- | --- | --- | --- | --- | --- | --- | --- | --- | --- | --- | --- | --- | --- |
|  | **Female** | **Male** |  |  | **Female** | **Male** |  |  | **Female** | | **Male** |  |  | **Female** | | **Male** |  |  |  |
|  | Median  (IQR) | Median  (IQR) | **p-value** | **Cohen's d** | Median  (IQR) | Median  (IQR) | **p-value** | **Cohen's d** | Median  (IQR) | | Median  (IQR) | **p-value** | **Cohen's d** | Median  (IQR) | | Median  (IQR) | **p-value** | **Cohen's d** |  |
| Overall | **1 (1-1)** | **1 (1-2)** | **3.0e-4** | **-0.20 [-0.35; -0.06]** | **1 (1-1)** | **1 (1-2)** | **0.03** | **-0.17 [-0.33; -0.01]** | 10 (2-31.8) | | 11.5 (2-39.8) | 0.38 | -0.17 [-0.31; -0.02] | **10 (2-34.2)** | | **15.5 (3-48.2)** | **0.01** | **-0.16 [-0.32; -0.003]** |  |
| 2024 | 1 (1-1) | 1 (1-1) | 0.36 | -0.15 [-0.46; 0.17] | 1 (1-1) | 1 (1-1) | 0.22 | -0.24 [-0.57; 0.10] | 0 (0-1.25) | | 0 (0-1) | 0.94 | -0.04 [-0.35; 0.27] | 0 (0-1) | | 1 (0-2) | 0.23 | -0.13 [-0.46; 0.21] |  |
| 2023 | 1 (1-1) | 1 (1-1) | 0.99 | -0.004 [-0.40; 0.39] | 1 (1-1) | 1 (1-1) | 0.10 | -0.34 [-0.76; 0.08] | 3 (1-6) | | 2 (1-6.5) | 0.74 | -0.22 [-0.61; 0.18] | 2 (1-5.5) | | 4 (2-6.75) | 0.11 | -0.30 [-0.72; 0.12] |  |
| 2022 | 1 (1-1) | 1 (1-1) | 0.93 | 0.01 [-0.31; 0.33] | 1 (1-1) | 1 (1-1) | 0.53 | 0.07 [-0.22; 0.40] | 9 (4-17.5) | | 9 (5-21.2) | 0.53 | -0.19 [-0.51; 0.13] | 10.5 (5-23.2) | | 11 (5-22) | 0.96 | -0.08 [-0.41; 0.25] |  |
| 2021 | 1 (1-1) | 1 (1-1) | 0.67 | 0.11 [-0.26; 0.49] | 1 (1-1) | 1 (1-1) | 0.70 | -0.13 [-0.54; 0.28] | **11.5 (5.25-30)** | | **19.5 (12.8-36.2)** | **0.02** | **-0.23 [-0.60; 0.15]** | 14.5 (7.75-31.2) | | 18 (8.5-30) | 0.65 | 0.07 [-0.34; 0.47] |  |
| 2020 | 1 (1-1) | 1 (1-1) | 0.36 | 0.26 [-0.18; 0.70] | **1 (1-1)** | **1 (1-1)** | **0.01** | **0.51 [0.02; 0.99]** | 19.5 (10-30.8) | | 19.5 (8.25-38.5) | 0.95 | -0.28 [-0.72; 0.16] | 20 (12-37) | | 19 (11-38) | 0.85 | -0.14 [-0.61; 0.34] |  |
| 2019 | 1 (1-1) | 1 (1-1) | 0.30 | -0.32 [-0.81; 0.17] | 1 (1-1) | 1 (1-1) | 0.24 | -0.38 [-0.95; 0.20] | 18 (11.5-64) | | 21 (6-39.8) | 0.32 | 0.15 [-0.34; 0.64] | 15 (11-55.8) | | 40 (14.5-71) | 0.31 | -0.39 [-0.97; 0.19] |  |
| 2018 | 1 (1-1) | 1 (1-1) | 0.96 | -0.09 [-0.61; 0.42] | 1 (1-1) | 1 (1-1) | 0.67 | -0.005 [-0.57; 0.56] | 24 (18.5-39.5) | | 33 (16-68.5) | 0.40 | -0.25 [-0.77; 0.27] | **39.5 (31.5-77.2)** | | **23 (16-41)** | **0.04** | **0.67 [0.08; 1.25]** |  |
| 2017 | **1 (1-1)** | **1 (1-1)** | **0.03** | **-0.67 [-1.26; -0.07]** | 1 (1-1) | 1 (1-1) | 0.18 | -0.46 [-1.11; 0.20] | 53.5 (27.5-96.8) | | 21 (6-59) | 0.06 | 0.48 [-0.11; 1.06] | 46 (14-67) | | 37.5 (9.5-84.5) | 0.70 | -0.008 [-0.66; 0.64] |  |
| 2016 | 1 (1-1) | 1 (1-1) | 0.96 | -0.32 [-1.08; 0.44] | 1 (1-1) | 1 (1-1) | 0.54 | -0.32 [-1.23; 0.59] | 35 (28.5-73.5) | | 37 (18-120) | 1.00 | -0.38 [-1.13; 0.39] | 28 (21.5-38.5) | | 45 (20.2-108) | 0.32 | -0.60 [-1.52; 0.34] |  |
| 2015 | 1 (1-1) | 1 (1-1) | 0.68 | 0.12 [-0.85; 1.09] | 1 (1-1) | 1 (1-1) | 0.48 | -0.40 [-1.53; 0.734] | 154 (26-258) | | 39 (23-147) | 0.48 | 0.41 [-0.57; 1.38] | 13.5 (11.8-29.8) | | 62 (23-188) | 0.09 | -0.72 [-1.85; 0.45] |  |
| 2014 | 1 (1-1) | 1 (1-1) | 0.77 | -0.27 [-1.24; 0.70] | 1 (1-1) | 1 (1-1) | 0.67 | -0.40 [-1.89; 1.1] | 45 (14-96) | | 58.5 (22.5-130) | 0.56 | 0.03 [-0.93; 1.00] | 79 (62-96) | | 51 (20-141) | 0.93 | -0.13 [-1.62; 1.36] |  |
| 2013 | 1 (1-1) | 1 (1-1) | 0.62 | -0.39 [-1.57; 0.81] | 0 | 1 (1-1) | NC | NC | 42.5 (27.2-69.8) | | 53 (9-95) | 0.83 | -0.08 [-1.26; 1.10] | 0 | | 51.5 (28.2-98) | NC | NC |  |
| 2012 | 1 (1-1) | 1 (1-1) | 1.00 | -0.45 [-2.59; 1.75] | 1 (1-1) | 1 (1-1) | 1.00 | 0 [0; 0] | 31 (31-31) | | 200 (111-270) | 0.67 | -0.84 [-3.01; 1.43] | 31 (31-31) | | 135 (123-168) | 0.50 | -2.56 [-5.69; 0.79] |  |
| 2011 | 1 (1-1) | 1.5 (1-2) | 0.26 | -1.12 [-2.72; 0.57] | 1 (1-1) | 1 (1-1.25) | 1.00 | -0.5 [-2.69; 1.76] | 31 (22-81.5) | | 71.5 (55.5-152) | 0.63 | -0.59 [-2.10; 0.98] | 132 (132-132) | | 33.5 (30.8-122) | 0.80 | 0.08 [-2.12; 2.26] |  |
| 2010 | 0 | 1 (1-1) | NC | NC | 0 | 1 (1-1) | NC | NC | 0 | | 45 (24-55.5) | NC | NC | 0 | | 45 (45-45) | NC | NC |  |
| 2005 | 0 | 1 (1-1) | NC | NC | NC | NC | NC | NC | 0 | | 1457 (1457-1457) | NC | NC | NC | | NC | NC | NC |  |

*Note*: IQR: interquartile range, NC: not calculated. The p-value corresponds to the Wilcoxon Mann-Whitney rank sum test. In green: small effect size, in orange: medium effect size, in red: large effect size.

# **Table A.9**: Total number of publications and citations by primary and senior authorship pairs

| **Author pair** | **Number of publications** | | | | | **Number of citations** | | | | |
| --- | --- | --- | --- | --- | --- | --- | --- | --- | --- | --- |
|  | **median (IQR)** | **Chi-squared** | **df** | **p value** | **Cohen's d** | **median (IQR)** | **Chi-squared** | **df** | **p value** | **Cohen's d** |
| Female primary and senior  (201 publications, 3890 citations) | 1 (1-1) | 18.18 | 3 | 4.38e-4 | -0.27 [-0.39; -0.13] | 8.5 (2-30) | 15.913 | 3 | 0.001181 | -0.25 [-0.37; -0.11] |
| Female primary and male senior  (260 publications, 7077 citations) | 1 (1-2) |  |  |  |  | 15 (4-45) |  |  |  |  |
| Male primary and female senior  (116 publications, 2404 citations) | 1 (1-2) |  |  |  |  | 13 (2-42) |  |  |  |  |
| Male primary and senior  (309 publications, 8554 citations) | 1 (1-2) |  |  |  |  | 12 (2-45) |  |  |  |  |

*Note*: IQR: interquartile range. The p-value corresponds to the Kruskall-Wallis test. In orange: medium effect size.

# **Table A.10**: Number of publications and citations over time, stratified by authorship and sex, considering only rank 1 journals

| **Year** | **Number of publications by primary authorship** | | | | | **Number of publications by senior authorship** | | | | **Number of citations by primary authorship** | | | | **Number of citations by senior authorship** | | | |
| --- | --- | --- | --- | --- | --- | --- | --- | --- | --- | --- | --- | --- | --- | --- | --- | --- | --- |
|  | **Female** | **Male** |  |  | **Female** | | **Male** |  |  | **Female** | **Male** |  |  | **Female** | **Male** |  |  |
|  | Median  (IQR) | Median  (IQR) | **p-value** | **Cohen's d** | Median  (IQR) | | Median  (IQR) | **p-value** | **Cohen's d** | Median  (IQR) | Median  (IQR) | **p-value** | **Cohen's d** | Median  (IQR) | Median  (IQR) | **p-value** | **Cohen's d** |
| Overall | **1 (1-1)** | **1 (1-1)** | **0.04** | **-0.134 [-0.311; 0.042]** | 1 (1-1) | | 1 (1-2) | 0.11 | -0.169 [-0.367; 0.029] | 12 (3-36) | 15 (2.25-50) | 0.25 | -0.163 [-0.339; 0.0139] | **12.5 (3-39.2)** | **22.5 (4.25-62)** | **0.008** | **-0.235 [-0.433; -0.04]** |
| 2024 | 1 (1-1) | 1 (1-1) | 0.32 | -0.184 [-0.543; 0.176] | 1 (1-1) | | 1 (1-1) | 0.36 | -0.201 [-0.588; 0.186] | 0 (0-2) | 0 (0-2) | 0.936 | -0.0301 [-0.389; 0.329] | 0 (0-1) | 1 (0-2) | 0.181 | -0.103 [-0.49; 0.284] |
| 2023 | 1 (1-1) | 1 (1-1) | 0.93 | 0.0248 [-0.448; 0.498] | **1 (1-1)** | | **1 (1-1)** | **0.05** | **-0.425 [-0.933; 0.0867]** | 4 (1.75-8.25) | 3 (2-9) | 0.865 | -0.205 [-0.678; 0.271] | 2.5 (1-9) | 4 (2-8.25) | 0.434 | -0.295 [-0.801; 0.214] |
| 2022 | 1 (1-1) | 1 (1-1) | 0.45 | 0.15 [-0.24; 0.539] | 1 (1-1) | | 1 (1-1) | 0.97 | -0.0534 [-0.458; 0.351] | 10 (6-19.5) | 12 (6-24.5) | 0.54 | -0.241 [-0.631; 0.15] | 11 (6-25.5) | 12 (7-25) | 0.41 | -0.185 [-0.59; 0.221] |
| 2021 | 1 (1-1) | 1 (1-1) | 0.72 | 0.0881 [-0.386; 0.561] | 1 (1-1) | | 1 (1-1) | 0.94 | -0.0217 [-0.528; 0.484] | 17 (8-31.8) | 22 (15-41.2) | 0.083 | -0.354 [-0.829; 0.125] | 21.5 (12.8-40.5) | 19.5 (14.5-34) | 0.776 | 0.201 [-0.307; 0.707] |
| 2020 | 1 (1-1) | 1 (1-1) | 0.30 | 0.342 [-0.23; 0.91] | 1 (1-1) | | 1 (1-1) | 0.08 | 0.472 [-0.162; 1.1] | 19.5 (14.2-39.5) | 33 (11.8-90.2) | 0.12 | -0.5 [-1.07; 0.0772] | 25 (14.5-52) | 30 (15-48) | 1 | -0.115 [-0.739; 0.509] |
| 2019 | 1 (1-1) | 1 (1-1) | 0.61 | -0.264 [-0.872; 0.347] | 1 (1-1) | | 1 (1-1) | 0.74 | -0.167 [-0.855; 0.523] | 44 (16.5-70.5) | 33 (15-43) | 0.329 | 0.18 [-0.43; 0.787] | 19 (12-65) | 43 (17.5-83) | 0.383 | -0.332 [-1.02; 0.362] |
| 2018 | 1 (1-1) | 1 (1-1) | 0.32 | 0.354 [-0.309; 1.01] | 1 (1-1) | | 1 (1-1) | 0.27 | 0.413 [-0.288; 1.11] | 35 (22-66) | 53 (32-85.5) | 0.228 | -0.329 [-0.986; 0.332] | 51.5 (33.8-94.2) | 35 (22-61) | 0.117 | 0.641 [-0.0723; 1.34] |
| 2017 | 1 (1-1) | 1 (1-1) | 0.28 | -0.467 [-1.25; 0.327] | 1 (1-1) | | 1 (1-1) | 1 | 0 [0; 0] | 67 (36.5-126) | 50 (33.5-98.5) | 0.586 | 0.338 [-0.449; 1.12] | 46.5 (37.2-86.8) | 71 (28-120) | 0.771 | -0.227 [-1.07; 0.618] |
| 2016 | 1 (1-1) | 1 (1-1) | 1 | 0 [0; 0] | 1 (1-1) | | 1 (1-1) | 1 | 0 [0; 0] | 50 (34.2-78.2) | 52 (18-120) | 0.953 | -0.176 [-1.25; 0.903] | 34 (28-43) | 64 (35-117) | 0.147 | -0.961 [-2.1; 0.214] |
| 2015 | 1 (1-1.25) | 1 (1-1) | 0.64 | 0.083 [-1.08; 1.24] | 1 (1-1) | | 1 (1-1) | 0.48 | -0.527 [-1.84; 0.812] | 254 (199-305) | 87 (27-148) | 0.142 | 0.722 [-0.487; 1.9] | 14 (11-45.5) | 148 (62-262) | 0.06 | -0.998 [-2.35; 0.4] |
| 2014 | 1 (1-1.5) | 1 (1-1) | 0.30 | 1 [-0.57; 2.5] | 0 | | 1 (1-1) | NC | NC | 51 (30.5-250) | 43 (30-74) | 1 | 0.532 [-0.95; 1.97] | 0 | 51 (23.5-165) | NC | NC |
| 2013 | 1 (1-1) | 1 (1-1) | 1 | 0 [0; 0] | 0 | | 1 (1-1) | NC | NC | 42.5 (38.8-46.2) | 53 (46.5-67.5) | 0.4 | -0.852 [-2.7; 1.11] | 0 | 50 (40-53) | NC | NC |
| 2012 | 1 (1-1) | 1 (1-1) | 1 | 0 [0; 0] | 1 (1-1) | | 1 (1-1) | 1 | 0 [0; 0] | 31 (31-31) | 79.5 (43.5-280) | 0.8 | -0.586 [-2.78; 1.69] | 31 (31-31) | 111 (111-111) | 1 | 0 |
| 2011 | 0 | 1 (1-1) | NC | NC | 0 | | 1 (1-1) | NC | NC | 0 | 24 (24-24) | NC | NC | 0 | 45 (45-45) | NC | NC |
| 2010 | 0 | 1 (1-1) | NC | NC | 1 (1-1) | | 1 (1-2) | 0.11 | -0.169 [-0.367; 0.029] | 0 | 55.5 (50.2-60.8) | NC | NC | 12.5 (3-39.2) | 22.5 (4.25-62) | 0.008 | -0.235 [-0.433; -0.04] |
| 2005 | 0 | 1 (1-1) | NC | NC | 0 | | 1 | NC | NC | 0 | 1457 (1457-1457) | NC | NC | 0 (0-1) | 1 (0-2) | 0.181 | -0.103 [-0.49; 0.284] |

*Note*: IQR: interquartile range, NC: not calculated. The p-value corresponds to the Wilcoxon Mann-Whitney rank sum test. In green: small effect size, in orange: medium effect size, in red: large effect size.

# **Table A.11**: Total number of publications and citations by primary and senior authorship pairs, considering only rank 1 journals

| **Author pair** | **Number of publications** | | | | | **Number of citations** | | | | |
| --- | --- | --- | --- | --- | --- | --- | --- | --- | --- | --- |
|  | **median (IQR)** | **Chi-squared** | **df** | **p value** | **Cohen's d** | **median (IQR)** | **Chi-squared** | **df** | **p value** | **Cohen's d** |
| Female primary and senior  (129 publications, 2937 citations) | 1 (1-1) | 8.386 | 3 | 0.03867 | -0.25 [-0.43; -0.08] | 10 (3-33) | 13.007 | 3 | 0.004621 | -0.26 [-0.43; -0.1] |
| Female primary and male senior  (172 publications, 5657 citations) | 1 (1-2) |  |  |  |  | 19 (6-54.5) |  |  |  |  |
| Male primary and female senior  (81 publications, 2025 citations) | 1 (1-2) |  |  |  |  | 14.5 (5-42) |  |  |  |  |
| Male primary and senior  (186 publications, 6327 citations) | 1 (1-2) |  |  |  |  | 18 (3-57.2) |  |  |  |  |

*Note*: IQR: interquartile range. The p-value corresponds to the Kruskall-Wallis test. In orange: medium effect size.

# **Table A.12**: Number of publications and citations over time, stratified by authorship and sex, considering only journals with impact factor ≥ 5

| **Year** | **Number of publications by primary authorship** | | | | | **Number of publications by senior authorship** | | | | | **Number of citations by primary authorship** | | | | | **Number of citations by senior authorship** | | | | |
| --- | --- | --- | --- | --- | --- | --- | --- | --- | --- | --- | --- | --- | --- | --- | --- | --- | --- | --- | --- | --- |
|  | **Female** | **Male** |  |  | **Female** | | **Male** |  |  | **Female** | | **Male** |  |  | **Female** | | **Male** |  |  |  |
|  | Median  (IQR) | Median  (IQR) | **p-value** | **Cohen's d** | Median  (IQR) | | Median  (IQR) | **p-value** | **Cohen's d** | Median  (IQR) | | Median  (IQR) | **p-value** | **Cohen's d** | Median  (IQR) | | Median  (IQR) | **p-value** | **Cohen's d** |  |
| Overall | 1 (1-1) | 1 (1-1) | 0.3 | -0.087 [-0.287; 0.113] | 1 (1-1) | | 1 (1-2) | 0.0821 | -0.148 [-0.373; 0.0774] | 13 (4-39) | | 17 (3-52) | 0.319 | -0.20 [-0.401; -0.0009] | 17 (3.75-43.5) | | 20.5 (4-56.2) | 0.245 | -0.175 [-0.4; 0.0507] |  |
| 2024 | 1 (1-1) | 1 (1-1) | 0.99 | -0.005 [-0.436; 0.425] | 1 (1-1) | | 1 (1-1) | 0.511 | -0.161 [-0.637; 0.315] | 0.5 (0-2) | | 0 (0-2) | 0.828 | -0.0326 [-0.463; 0.398] | 0 (0-1) | | 1 (0-2) | 0.517 | 0.027 [-0.448; 0.503] |  |
| 2023 | 1 (1-1) | 1 (1-1) | 0.375 | -0.257 [-0.803; 0.292] | 1 (1-1) | | 1 (1-1) | 0.0714 | -0.506 [-1.12; 0.109] | 4 (1-9.5) | | 3.5 (1.75-10) | 0.832 | -0.262 [-0.809; 0.287] | 2 (1-11) | | 4.5 (2-10.8) | 0.517 | -0.288 [-0.892; 0.32] |  |
| 2022 | 1 (1-1) | 1 (1-1) | 0.11 | 0.35 [-0.0767; 0.774] | 1 (1-1) | | 1 (1-1) | 0.42 | -0.211 [-0.643; 0.223] | 11 (6-21.5) | | 14 (8-29) | 0.218 | -0.331 [-0.755; 0.095] | 12.5 (6.25-27.5) | | 13 (9-27) | 0.599 | -0.145 [-0.577; 0.288] |  |
| 2021 | 1 (1-1) | 1 (1-1) | 0.423 | 0.199 [-0.284; 0.68] | 1 (1-1) | | 1 (1-1) | 0.912 | -0.0677 [-0.583; 0.449] | 17 (9-34) | | 24 (15.5-41.5) | 0.126 | -0.314 [-0.797; 0.17] | 21.5 (13.2-41.5) | | 19.5 (12.2-33.8) | 0.581 | 0.25 [-0.269; 0.767] |  |
| 2020 | 1 (1-1) | 1 (1-1) | 0.538 | 0.258 [-0.357; 0.87] | 1 (1-1) | | 1 (1-1) | 0.0913 | 0.478 [-0.184; 1.13] | **19.5 (12.8-36.8)** | | **37 (22-97)** | **0.0215** | **-0.662 [-1.29; -0.0306]** | 23.5 (17.2-57) | | 30.5 (15.2-45.8) | 0.854 | -0.0709 [-0.72; 0.579] |  |
| 2019 | 1 (1-1) | 1 (1-1) | 0.684 | -0.308 [-1.02; 0.407] | 1 (1-1) | | 1 (1-1) | 0.818 | -0.17 [-0.95; 0.614] | 46 (17-72) | | 36 (21-47.5) | 0.549 | 0.114 [-0.597; 0.823] | 19 (13-69) | | 43 (23-79) | 0.541 | -0.314 [-1.1; 0.474] |  |
| 2018 | 1 (1-1) | 1 (1-1) | 1 | 0 [0; 0] | 1 (1-1) | | 1 (1-1) | 1 | 0 [0; 0] | 46 (31.8-70.2) | | 55 (33.8-95.8) | 0.41 | -0.418 [-1.19; 0.366] | 55 (35.2-102) | | 47.5 (33.2-70) | 0.355 | 0.468 [-0.349; 1.27] |  |
| 2017 | 1 (1-1) | 1 (1-1) | 1 | 0 [0; 0] | 1 (1-1) | | 1 (1-1) | 1 | 0 [0; 0] | 78 (45.5-128) | | 98.5 (70-114) | 0.887 | 0.102 [-1.06; 1.26] | 57 (45.5-126) | | 98.5 (78-122) | 0.897 | -0.0609 [-1.12; 0.999] |  |
| 2016 | 1 (1-1) | 1 (1-1) | 1 | 0 [0; 0] | 1 (1-1) | | 1 (1-1) | 1 | 0 [0; 0] | 57 (34-64) | | 52 (35-86) | 0.786 | 0.0354 [-1.4; 1.47] | 34 (26-45.5) | | 64 (52-120) | 0.25 | -1.13 [-2.65; 0.472] |  |
| 2015 | 1 (1-1) | 1 (1-1) | 0.752 | -0.5 [-2.14; 1.19] | 1 (1-1) | | 1 (1-1) | 1 | -0.447 [-2.59; 1.75] | 254 (250-259) | | 148 (77-188) | 0.381 | 0.639 [-1.07; 2.29] | 77 (77-77) | | 188 (148-245) | 0.667 | -0.946 [-3.13; 1.34] |  |
| 2014 | 1.5 (1.25-1.75) | 1 (1-1) | 0.617 | 1 [-1.25; 3.07] | 0 | | 1 (1-1) | NC | NC | 250 (151-350) | | 150 (96.5-204) | 0.667 | 0.444 [-1.61; 2.4] | 0 | | 256 (154-256) | NC | NC |  |
| 2013 | 1 (1-1) | 1 (1-1) | 1 | 0 [0; 0] | 0 | | 1 (1-1) | NC | NC | 50 (50-50) | | 53 (46.5-67.5) | 1 | -0.388 [-2.64; 1.95] | 0 | | 51.5 (47.5-60.2) | NC | NC |  |
| 2012 | 0 | 1 (1-1) | NC | NC | 0 | | 1 (1-1) | NC | NC | 0 | | 448 (280-617) | NC | NC | 0 | | 111 (111-111) | NC | NC |  |
| 2011 | 0 | 0 | NC | NC | 0 | | 0 | NC | NC | 0 | | 66 (66-66) | NC | NC | 0 | | 0 | NC | NC |  |
| 2010 | 0 | 1 (1-1) | NC | NC | 0 | | 0 | NC | NC | 0 | | 0 | NC | NC | 0 | | 0 | NC | NC |  |
| 2005 | 0 | 0 | NC | NC | 0 | | 0 | NC | NC | 13 (4-39) | | 17 (3-52) | 0.319 | -0.20 [-0.401; -0.0009] | 0 | | 0 | NC | NC |  |

*Note*: IQR: interquartile range, NC: not calculated. The p-value corresponds to the Wilcoxon Mann-Whitney rank sum test. In green: small effect size, in orange: medium effect size, in red: large effect size.

# **Table A.13**: Total number of publications and citations by primary and senior authorship pairs, considering only journals with impact factor ≥ 5

| **Author pair** | **Number of publications** | | | | | **Number of citations** | | | | |
| --- | --- | --- | --- | --- | --- | --- | --- | --- | --- | --- |
|  | **median (IQR)** | **Chi-squared** | **df** | **p value** | **Cohen's d** | **median (IQR)** | **Chi-squared** | **df** | **p value** | **Cohen's d** |
| Female primary and senior  (98 publications, 2510 citations) | 1 (1-1) | 9.3554 | 3 | 0.02492 | -0.25 [-0.46; -0.05] | 13 (3-40) | 4.4019 | 3 | 0.2212 | -0.17 [-0.37; 0.03] |
| Female primary and male senior  (141 publications, 3983 citations) | 1 (1-2) |  |  |  |  | 18 (6-45) |  |  |  |  |
| Male primary and female senior  (58 publications, 1794 citations) | 1 (1-2) |  |  |  |  | 22 (8-51) |  |  |  |  |
| Male primary and senior  (141 publications, 4991 citations) | 1 (1-2) |  |  |  |  | 20 (3-62) |  |  |  |  |

*Note*: IQR: interquartile range. The p-value corresponds to the Kruskall-Wallis test. In orange: medium effect size.

# **Table A.14**: Number of publications and citations over time, stratified by authorship and sex, considering only journals with impact factor ≥ 10

| **Year** | **Number of publications by primary authorship** | | | | | **Number of publications by senior authorship** | | | | **Number of citations by primary authorship** | | | | **Number of citations by senior authorship** | | | |
| --- | --- | --- | --- | --- | --- | --- | --- | --- | --- | --- | --- | --- | --- | --- | --- | --- | --- |
|  | **Female** | **Male** |  |  | **Female** | | **Male** |  |  | **Female** | **Male** |  |  | **Female** | **Male** |  |  |
|  | Median  (IQR) | Median  (IQR) | **p-value** | **Cohen's d** | Median  (IQR) | | Median  (IQR) | **p-value** | **Cohen's d** | Median  (IQR) | Median  (IQR) | **p-value** | **Cohen's d** | Median  (IQR) | Median  (IQR) | **p-value** | **Cohen's d** |
| Overall | 1 (1-1) | 1 (1-1) | 0.519 | -0.164 [-0.471; 0.144] | 1 (1-1) | | 1 (1-1) | 0.608 | -0.0726 [-0.42; 0.275] | **10.5 (1-31.2)** | **18 (2-55)** | **0.0438** | **-0.365 [-0.674; -0.055]** | 19 (8-40) | 18 (2-43.5) | 0.871 | -0.0728 [-0.42; 0.275] |
| 2024 | 1 (1-1) | 1 (1-1) | 0.413 | 0.269 [-0.346; 0.881] | 1 (1-1) | | 1 (1-1) | 0.758 | 0.124 [-0.593; 0.839] | 0 (0-1.25) | 1 (0-2) | 0.35 | -0.254 [-0.866; 0.362] | 0 (0-0.75) | 1 (0-2) | 0.325 | -0.134 [-0.85; 0.583] |
| 2023 | 1 (1-1) | 1 (1-1) | 1 | 0 [0; 0] | 1 (1-1) | | 1 (1-1) | 0.132 | -0.659 [-1.48; 0.176] | 2 (1-10.5) | 3 (1.5-18.5) | 0.645 | -0.422 [-1.14; 0.306] | 2 (1-14) | 5 (2-24) | 0.606 | -0.318 [-1.13; 0.499] |
| 2022 | 1 (1-1) | 1 (1-1) | 0.181 | 0.408 [-0.179; 0.991] | 1 (1-1) | | 1 (1-1) | 0.139 | -0.473 [-1.08; 0.144] | 13.5 (10.8-30.5) | 20.5 (12.2-34.8) | 0.266 | -0.345 [-0.926; 0.24] | 17 (12-34) | 17 (11-32) | 0.684 | -0.067 [-0.673; 0.539] |
| 2021 | 1 (1-1) | 1 (1-1) | 0.258 | -0.407 [-1.09; 0.28] | 1 (1-1) | | 1 (1-1) | 0.336 | 0.356 [-0.351; 1.06] | 26.5 (16.5-41.5) | 28 (18-42) | 0.674 | -0.142 [-0.818; 0.536] | 31 (16-43) | 24 (17.8-36.2) | 0.672 | 0.177 [-0.524; 0.875] |
| 2020 | 1 (1-1) | 1 (1-1) | 1 | 0 [0; 0] | 1 (1-1) | | 1 (1-1) | 1 | 0 [0; 0] | 38 (15-39) | 100 (97-109) | 0.0556 | -1.04 [-2.35; 0.323] | 100 (100-100) | 39 (32-97) | 0.6 | 0.0193 [-2.05; 2.08] |
| 2019 | 1 (1-1) | 1 (1-1) | 1 | 0 [0; 0] | 1 (1-1) | | 1 (1-1) | 1 | 0 [0; 0] | 104 (104-105) | 61 (61-61) | 0 | 0 | 104 (104-104) | 83 (72-94) | 1 | 0.675 [-1.97; 3.09] |
| 2018 | 0 | 1 (1-1) | NC | NC | 1 (1-1) | | 1 (1-1) | 1 | 0 [0; 0] | 0 | 136 (127-407) | NC | NC | 136 (136-136) | 118 (118-118) | 1 | 0 |
| 2017 | 1 (1-1) | 1 (1-1) | 1 | 0 [0; 0] | 1 (1-1) | | 1 (1-1) | 1 | 0 [0; 0] | 89 (67-146) | 130 (130-130) | 1 | -0.211 [-2.46; 2.08] | 124 (84.8-164) | 110 (99.2-120) | 1 | 0.183 [-1.8; 2.13] |
| 2016 | 0 | 1 (1-1) | NC | NC | 0 | | 1 (1-1) | NC | NC | 0 | 120 (120-120) | NC | NC | 0 | 120 (120-120) | 0 | NC |
| 2015 | 0 | 0 | NC | NC | 0 | | 0 | NC | NC | 0 | 0 | NC | NC | 0 | 0 | NC | NC |
| 2014 | 0 | 0 | NC | NC | 0 | | 0 | NC | NC | 0 | 0 | NC | NC | 0 | 0 | NC | NC |
| 2013 | 0 | 0 | NC | NC | 0 | | 0 | NC | NC | 0 | 0 | NC | NC | 0 | 0 | NC | NC |
| 2012 | 0 | 0 | NC | NC | 0 | | 0 | NC | NC | 0 | 0 | NC | NC | 0 | 0 | NC | NC |
| 2011 | 0 | 0 | NC | NC | 0 | | 0 | NC | NC | 0 | 0 | NC | NC | 0 | 0 | NC | NC |
| 2010 | 0 | 0 | NC | NC | 0 | | 0 | NC | NC | 0 | 0 | NC | NC | 0 | 0 | NC | NC |
| 2005 | 0 | 0 | NC | NC | 0 | | 0 | NC | NC | 0 | 0 | NC | NC | 0 | 0 | NC | NC |

*Note*: IQR: interquartile range, NC: not calculated. The p-value corresponds to the Wilcoxon Mann-Whitney rank sum test. In green: small effect size, in orange: medium effect size, in red: large effect size.

# **Table A.15**: Total number of publications and citations by primary and senior authorship pairs, considering only journals with impact factor ≥ 10

| **Author pair** | **Number of publications** | | | | | **Number of citations** | | | | |
| --- | --- | --- | --- | --- | --- | --- | --- | --- | --- | --- |
|  | **median (IQR)** | **Chi-squared** | **df** | **p value** | **Cohen's d** | **median (IQR)** | **Chi-squared** | **df** | **p value** | **Cohen's d** |
| Female primary and senior  (34 publications, 966 citations) | 1 (1-1) | 0.78462 | 3 | 0.8531 | -0.16 [-0.46; 0.15] | 23 (1-40) | 6.5624 | 3 | 0.08723 | 0.10 [-0.18; 0.47] |
| Female primary and male senior  (55 publications, 994 citations) | 1 (1-1) |  |  |  |  | 12 (2-30.5) |  |  |  |  |
| Male primary and female senior  (23 publications, 861 citations) | 1 (1-1) |  |  |  |  | 18 (12-37) |  |  |  |  |
| Male primary and senior  (61 publications, 2532 citations) | 1 (1-1) |  |  |  |  | 23 (2-61) |  |  |  |  |

*Note*: IQR: interquartile range. The p-value corresponds to the Kruskall-Wallis test. In orange: medium effect size.

# **Table A.16**: Top 20 of the most active institutions in the human exposome research field

| **Institution** | **Country** | **Publication start** | **Number of publications (%)** | **Total citation** | **Local citation (%)** | **Average citation**  **per year** | **Mean AGR** | **Fractionalized frequency (%)** | **h-index** | **g-index** | **m-index** |
| --- | --- | --- | --- | --- | --- | --- | --- | --- | --- | --- | --- |
| INSERM | France | 2014 | 132 (14.2) | 5038 | 543 (10.8) | 458.0 | 72.1 | 18.4 | 37 | 68 | 2.47 |
| CIBER | Spain | 2014 | 98 (10.5) | 3096 | 318 (10.3) | 281.5 | 3.04 | 9.32 | 29 | 54 | 1.93 |
| Pompeu Fabra University (UPF) | Spain | 2014 | 95 (10.2) | 2560 | 275 (10.8) | 232.7 | 24.8 | 9.47 | 25 | 48 | 1.67 |
| Isglobal | Spain | 2016 | 93 (9.99) | 2508 | 282 (11.3) | 278.7 | 38.7 | 8.58 | 25 | 48 | 1.67 |
| CNRS | France | 2010 | 80 (8.59) | 2720 | 311 (11.4) | 181.3 | 20.3 | 9.72 | 27 | 51 | 1.80 |
| Universite Grenoble Alpes (UGA) | France | 2010 | 75 (8.06) | 2191 | 200 (9.14) | 146.1 | 9.2 | 7.67 | 24 | 46 | 1.60 |
| Imperial College London | UK | 2012 | 74 (7.95) | 2710 | 427 (15.8) | 208.5 | 61.1 | 11.8 | 24 | 51 | 1.60 |
| Utrecht University | The Netherlands | 2015 | 60 (6.44) | 1575 | 172 (10.9) | 157.5 | 85.6 | 9.57 | 23 | 38 | 1.44 |
| Universite Paris Cite | France | 2015 | 54 (5.8) | 2582 | 299 (11.6) | 258.2 | 26.5 | 7.11 | 22 | 50 | 1.47 |
| Norwegian Institute of Public Health (NIPH) | Norway | 2014 | 52 (5.59) | 1291 | 153 (11.9) | 117.4 | 11.8 | 3.47 | 20 | 35 | 1.33 |
| Harvard University | US | 2012 | 50 (5.37) | 1770 | 191 (10.8) | 136.2 | 33.0 | 11.4 | 21 | 42 | 1.62 |
| University Of Southern California | US | 2013 | 50 (5.37) | 1262 | 152 (12.1) | 105.2 | 17.9 | 6.42 | 19 | 35 | 1.27 |
| Emory University | US | 2011 | 47 (5.05) | 1316 | 163 (12.4) | 94.0 | 27.7 | 17.2 | 19 | 36 | 1.19 |
| Icahn School Of Medicine At Mount Sinai | US | 2012 | 45 (4.83) | 1327 | 205 (15.4) | 102.1 | 92.8 | 16.0 | 20 | 36 | 1.67 |
| Vytautas Magnus University | Lithuania | 2014 | 44 (4.73) | 1193 | 134 (11.3) | 108.5 | 14.8 | 2.88 | 19 | 34 | 1.58 |
| University Of Crete | Greece | 2018 | 43 (4.62) | 1735 | 122 (7.04) | 247.9 | 9.4 | 3.11 | 19 | 41 | 1.27 |
| University Of California System | US | 2010 | 41 (4.4) | 1457 | 156 (10.7) | 97.1 | 8.42 | 10.7 | 18 | 38 | 1.50 |
| National Institutes of Health (NIH) | US | 2010 | 36 (3.87) | 1353 | 118 (8.72) | 90.2 | 46.5 | 11.3 | 20 | 36 | 1.67 |
| Maastricht University | The Netherlands | 2015 | 36 (3.87) | 755 | 48 (6.37) | 75.5 | 25.6 | 3.61 | 15 | 27 | 1.25 |
| Utrecht University Medical Center | The Netherlands | 2011 | 35 (3.76) | 954 | 118 (12.4) | 68.1 | 25.6 | 5.90 | 17 | 30 | 1.06 |
| INRAE | France | 2014 | 35 (3.76) | 717 | 54 (7.53) | 65.2 | 18.0 | 3.26 | 14 | 26 | 0.93 |

*Note*: AGR: annual growth rate, CNRS: Centre national de la recherche scientifique (French National Centre for Scientific Research), CIBERESP: Centro de Investigación Biomédica en Red (Spanish Biomedical Research Center), INRAE: Institut national de recherche pour l'agriculture, l'alimentation et l'environnement (French national research institute for agriculture, food and the environment), INSERM: Institut national de la santé et de la recherche médicale (French National Institute of Health and Medical Research), ISGlobal: Instituto de Salud Global de Barcelona (Global Health Institute of Barcelona).

Please refer to the beginning of the supplemental materials for the definition of the bibliometric indices.

# **Table A.17**: Top 20 of the most active funding bodies in the human exposome research field

| **Funding bodies** | **Country** | **Publication Period** | **Number of publications (%)** | **Total citation** | **Local citation (%)** | **Average citation**  **per year** | **Mean AGR** | **Fractionalized frequency (%)** | **h-index** | **g-index** | **m-index** |
| --- | --- | --- | --- | --- | --- | --- | --- | --- | --- | --- | --- |
| National Institutes Of Health (NIH) | US | 2010-2024 | 183 (19.7) | 9821 | 1007 (10.3) | 654.7 | 43.3 | 60.2 | 56 | 94 | 3.5 |
| National Institute Of Environmental Health Sciences (NIEHS) | US | 2010-2024 | 140 (15) | 6792 | 684 (10.1) | 452.8 | 56.7 | 50.6 | 47 | 79 | 2.94 |
| European Commission | Europe | 2012-2024 | 101 (10.8) | 4824 | 869 (18) | 371.1 | 41.0 | 22.3 | 38 | 68 | 2.71 |
| European Union | Europe | 2015-2024 | 96 (10.3) | 1991 | 239 (12) | 199.1 | 161.0 | 41.7 | 25 | 42 | 2.27 |
| UK Medical Research Council | UK | 2012-2024 | 58 (6.23) | 2485 | 471 (19) | 191.2 | 69.9 | 10.1 | 26 | 49 | 1.86 |
| National Natural Science Foundation | China | 2015-2024 | 47 (5.05) | 418 | 20 (4.78) | 41.8 | 17.6 | 19.2 | 12 | 20 | 1.09 |
| Generalitat De Catalunya | Spain | 2015-2024 | 45 (4.83) | 1393 | 330 (23.7) | 139.3 | 17.6 | 4.72 | 22 | 37 | 2 |
| UK Research and Innovation (UKRI) | UK | 2012-2023 | 37 (3.97) | 2169 | 442 (20.4) | 180.8 | 16.0 | 6.68 | 24 | 37 | 1.71 |
| CIBERESP | Spain | 2018-2024 | 31 (3.33) | 991 | 233 (23.5) | 141.6 | 36.4 | 2.00 | 15 | 31 | 1.88 |
| European Research Council (ERC) | Europe | 2015-2024 | 30 (3.22) | 1000 | 62 (6.26) | 100.0 | 11.7 | 7.53 | 14 | 30 | 1.27 |
| Instituto De Salud Carlos III | Spain | 2014-2022 | 28 (3.01) | 1290 | 276 (21.4) | 143.3 | 3.97 | 2.56 | 21 | 28 | 1.75 |
| Wellcome Trust | UK | 2014-2024 | 28 (3.01) | 750 | 89 (11.9) | 68.2 | 28.3 | 2.83 | 12 | 27 | 1 |
| Spanish Ministry Of Economy And Competitiveness | Spain | 2017-2024 | 27 (2.9) | 1275 | 267 (20.9) | 159.4 | 39.6 | 3.55 | 17 | 27 | 1.89 |
| Greek Ministry Of Health | Greece | 2018-2024 | 25 (2.69) | 1106 | 249 (22.5) | 158.0 | 34.3 | 1.53 | 17 | 25 | 2.12 |
| Lithuanian Agency For Science Innovation And Technology | Lithuania | 2018-2024 | 24 (2.58) | 1099 | 241 (21.9) | 157.0 | 4.76 | 1.55 | 17 | 24 | 2.12 |
| Norwegian Ministry Of Health And Care Services | Norway | 2018-2024 | 24 (2.58) | 774 | 166 (21.4) | 110.6 | 105.0 | 1.62 | 14 | 24 | 1.75 |
| French National Research Agency (ANR) | France | 2015-2024 | 23 (2.47) | 201 | 19 (9.45) | 20.1 | 116.0 | 9.32 | 7 | 14 | 0.636 |
| National Key RD Program Of China | China | 2018-2024 | 23 (2.47) | 171 | 17 (9.94) | 24.4 | 13.1 | 6.88 | 9 | 12 | 1.12 |
| Environmental Protection Agency (EPA) | US | 2010-2024 | 22 (2.36) | 711 | 78 (11) | 47.4 | 12.4 | 7.84 | 15 | 22 | 0.938 |
| Medline | US | 2011-2020 | 22 (2.36) | 1489 | 79 (5.31) | 148.9 | 30.0 | 7.70 | 19 | 22 | 1.27 |

*Note*: AGR: annual growth rate, CIBER: Centro de Investigación Biomédica en Red (Spanish Biomedical Research Center).

Please refer to the beginning of the supplemental materials for the definition of the bibliometric indices.

# **Table A.18**: Top 40 of the most frequent keywords in the human exposome publications

| **Keyword** | **First**  **appearance** | **Last**  **appearance** | **Number of**  **publications** | **Total**  **citation** | **Local**  **citation** | **Average citation**  **per year** | **Mean AGR** | **h-index** | **g-index** | **m-index** |
| --- | --- | --- | --- | --- | --- | --- | --- | --- | --- | --- |
| Exposome | 2005 | 2024 | 539 (57.9) | 16818 | 2029 (12.1) | 840.9 | 21.8 | 63 | 113 | 3 |
| Environment | 2005 | 2024 | 186 (20) | 6987 | 600 (8.59) | 349.4 | 28 | 43 | 80 | 2.05 |
| Chemicals | 2010 | 2024 | 140 (15) | 4471 | 389 (8.7) | 298.1 | 20.6 | 34 | 64 | 2.12 |
| Exposure | 2005 | 2024 | 128 (13.7) | 5857 | 615 (10.5) | 292.9 | 46.7 | 33 | 75 | 1.57 |
| Metabolomics | 2012 | 2024 | 101 (10.8) | 3184 | 513 (16.2) | 244.9 | 33.6 | 33 | 54 | 2.36 |
| Children | 2013 | 2024 | 81 (8.7) | 2200 | 428 (19.5) | 183.3 | 40.9 | 24 | 45 | 1.85 |
| Biomarkers | 2005 | 2024 | 73 (7.84) | 4841 | 656 (13.6) | 242.1 | 6.31 | 30 | 69 | 1.43 |
| Reproductive disorders | 2011 | 2024 | 70 (7.52) | 1380 | 119 (8.62) | 98.6 | 20.4 | 19 | 35 | 1.27 |
| Omics | 2013 | 2024 | 68 (7.3) | 2472 | 460 (18.6) | 206.0 | 31.7 | 24 | 49 | 1.85 |
| Pregnancy | 2011 | 2024 | 68 (7.3) | 1847 | 272 (14.7) | 131.9 | 14.1 | 24 | 42 | 1.6 |
| Air pollution | 2014 | 2024 | 68 (7.3) | 2633 | 90 (3.42) | 239.4 | 11.6 | 22 | 51 | 1.83 |
| Epidemiology | 2005 | 2024 | 66 (7.09) | 4248 | 595 (14) | 212.4 | 5.7 | 25 | 65 | 1.19 |
| Health | 2010 | 2024 | 66 (7.09) | 1122 | 137 (12.2) | 74.8 | 28.2 | 19 | 32 | 1.19 |
| Respiratory diseases | 2010 | 2024 | 60 (6.44) | 2015 | 157 (7.79) | 134.3 | 56.1 | 20 | 44 | 1.25 |
| Cancer | 2011 | 2024 | 56 (6.02) | 1948 | 370 (19) | 139.1 | 46.2 | 18 | 44 | 1.2 |
| Diet | 2013 | 2024 | 52 (5.59) | 1961 | 176 (8.98) | 163.4 | 53.7 | 18 | 44 | 1.38 |
| Human biomonitoring (HBM) | 2015 | 2024 | 52 (5.59) | 1126 | 169 (15) | 112.6 | 44.8 | 18 | 32 | 1.64 |
| High resolution mass spectrometry (HRMS) | 2016 | 2024 | 46 (4.94) | 649 | 140 (21.6) | 72.1 | 93.5 | 14 | 24 | 1.4 |
| Mental health disorders | 2015 | 2024 | 42 (4.51) | 573 | 39 (6.81) | 57.3 | 31.8 | 13 | 23 | 1.18 |
| Microbiome | 2012 | 2024 | 41 (4.4) | 1609 | 70 (4.35) | 123.8 | 24.7 | 20 | 40 | 1.43 |
| Artificial intelligence (AI) | 2013 | 2024 | 41 (4.4) | 425 | 58 (13.6) | 35.4 | 55.2 | 12 | 19 | 0.923 |
| Exposome-wide association study (EWAS) | 2012 | 2024 | 40 (4.3) | 1519 | 244 (16.6) | 116.8 | 57.5 | 19 | 38 | 1.36 |
| Socio-economic status (SES) | 2014 | 2024 | 39 (4.19) | 564 | 72 (12.8) | 51.3 | 40.8 | 14 | 23 | 1.17 |
| Occupation | 2010 | 2024 | 39 (4.19) | 538 | 78 (14.6) | 35.9 | 10.1 | 13 | 22 | 0.812 |
| Exposomics | 2013 | 2024 | 39 (4.19) | 426 | 52 (12.2) | 35.5 | 16.4 | 12 | 20 | 0.923 |
| Machine learning (ML) | 2013 | 2024 | 39 (4.19) | 319 | 36 (11.3) | 26.6 | 55.3 | 12 | 16 | 0.923 |
| Mass spectrometry (MS) | 2012 | 2024 | 35 (3.76) | 1145 | 154 (13.5) | 88.1 | 19.5 | 17 | 33 | 1.21 |
| Cardiovascular diseases (CVDs) | 2016 | 2024 | 35 (3.76) | 653 | 92 (14.1) | 72.6 | 40.9 | 13 | 25 | 1.3 |
| Diseases | 2011 | 2024 | 34 (3.65) | 1322 | 63 (4.79) | 94.4 | 27.9 | 15 | 34 | 1 |
| Database | 2011 | 2024 | 32 (3.44) | 1378 | 175 (12.7) | 98.4 | 58.3 | 17 | 32 | 1.13 |
| Pesticides | 2016 | 2024 | 31 (3.33) | 613 | 50 (8.16) | 68.1 | 56.7 | 14 | 24 | 1.4 |
| Data | 2011 | 2024 | 30 (3.22) | 891 | 69 (7.74) | 63.6 | 5.36 | 12 | 29 | 0.8 |
| Built environment | 2017 | 2024 | 30 (3.22) | 660 | 76 (11.5) | 82.5 | 43.3 | 11 | 25 | 1.22 |
| Cohorts | 2011 | 2024 | 28 (3.01) | 726 | 165 (22.7) | 51.9 | -10.2 | 13 | 26 | 0.867 |
| Epigenetics | 2015 | 2024 | 28 (3.01) | 675 | 33 (4.89) | 67.5 | 76.2 | 13 | 25 | 1.18 |
| Mixtures | 2017 | 2024 | 28 (3.01) | 353 | 53 (15) | 44.1 | 16.7 | 12 | 18 | 1.33 |
| Asthma | 2010 | 2024 | 27 (2.9) | 979 | 74 (7.56) | 65.3 | 30.3 | 13 | 27 | 0.812 |
| Non-targeted analysis (NTA) | 2015 | 2024 | 27 (2.9) | 397 | 71 (17.9) | 39.7 | 3.33 | 11 | 19 | 1 |
| Endocrine disrupting chemicals (EDCs) | 2015 | 2024 | 26 (2.79) | 801 | 41 (5.12) | 80.1 | 73.3 | 14 | 26 | 1.27 |
| Metals | 2016 | 2024 | 26 (2.79) | 473 | 45 (9.51) | 52.6 | 65.9 | 12 | 21 | 1.2 |
| Neurodegenerative diseases | 2016 | 2024 | 26 (2.79) | 491 | 18 (3.67) | 54.6 | 35.2 | 9 | 22 | 0.9 |

*Note*: AGR: annual growth rate. Please refer to the beginning of the supplemental materials for the definition of the bibliometric indices.

# **References**

[1] Bienert. I.R., Oliveira, R.C., Andrade, P.B., Caramori, C.A. Bibliometric indexes, databases and impact factors in cardiology. *Rev. Bras. Cir. Cardiovasc.* **30** (2), 254–259 (2015). <https://doi.org/10.5935/1678-9741.20150019>.

[2] Bihari, A., Tripathi, S., Deepak, A. A review on h-index and its alternative indices. *J. Inf. Sci.* **49** (3), 624–665 (2023). <https://doi.org/10.1177/0165551521101447>.

[3] Shao, Y., Chien, T.W., Jang, F.L. The use of radar plots with the Yk-index to identify which authors contributed the most to the journal of Medicine in 2020 and 2021: A bibliometric analysis. *Medicine (Baltimore).* **101** (45): e31033 (2022). <https://doi.org/10.1097%2FMD.0000000000031033>.

[4] Kumar, S., Kumar, S. Trends of collaborative research in journals of oilseeds research (India), 1993-2004. *Indian. J. Agric. Library. Inf. Services.* **24**, 80–90 (2008).

[5] Wu, T., Duan, Y., Zhang, T., Tian, W., Liu, H., Deng, Y. Research Trends in the Application of Artificial Intelligence in Oncology: A Bibliometric and Network Visualization Study. *Front. Biosci. (Landmark Ed)* **27** (9), 254 (2022). <https://doi.org/10.31083/j.fbl2709254>.

[6] Wild, C.P. Complementing the genome with an "exposome": the outstanding challenge of environmental exposure measurement in molecular epidemiology. *Cancer. Epidemiol. Biomarkers. Prev.* **14** (8), 1847–1850 (2005). <https://doi.org/10.1158/1055-9965.EPI-05-0456>.

[7] Wild, C.P. The exposome: from concept to utility. *Int. J. Epidemiol.* **41** (1), 24–32 (2012). <https://doi.org/10.1093/ije/dyr236>.

[8] Nikolich-Žugich, J. The twilight of immunity: emerging concepts in aging of the immune system. *Nat. Immunol.* **19** (1), 10–19 (2018). <https://doi.org/10.1038/s41590-017-0006-x>.

[9] Vermeulen, R., Schymanski, E.L., Barabási, A.L., Miller, G.W. The exposome and health: Where chemistry meets biology. *Science.* **367** (6476), 392–396 (2020). <https://doi.org/10.1126/science.aay3164>.

[10] Krutmann, J., Bouloc, A., Sore, G., Bernard, B.A., Passeron, T. The skin aging exposome. *J. Dermatol. Sci.* **85** (3), 152–161 (2017). <https://doi.org/10.1016/j.jdermsci.2016.09.015>.

[11] Rappaport, S.M. Implications of the exposome for exposure science. *J. Expo. Sci. Environ. Epidemiol.* **21** (1), 5–9 (2011). <https://doi.org/10.1038/jes.2010.50>.

[12] Gacesa, R., Kurilshikov, A., Vich Vila, A., Sinha, T., Klaassen, M. A. Y., Bolte, L. A., Andreu-Sánchez, S., Chen, L., Collij, V., Hu, S., Dekens, J. A. M., Lenters, V. C., Björk, J. R., Swarte, J. C., Swertz, M. A., Jansen, B. H., Gelderloos-Arends, J., Jankipersadsing, S., Hofker, M., Vermeulen, R. C. H., Sanna, S., Harmsen, H. J. M., Wijmenga, C., Fu, J., Zhernakova, A., Weersma, R. K. Environmental factors shaping the gut microbiome in a Dutch population. *Nature*. **604** (7907), 732-739 (2022). <https://doi.org/10.1038/s41586-022-04567-7>.

[13] Davis, A. P., Wiegers, T. C., Johnson, R. J., Sciaky, D., Wiegers, J., Mattingly, C. J. Comparative Toxicogenomics Database (CTD): update 2023. *Nucleic. Acids. Res.* **51** (D1), D1257-D1262 (2023). <https://doi.org/10.1093/nar/gkac833>.

[14] Go, Y.M., Chandler, J.D., Jones, D.P. The cysteine proteome. *Free. Radic. Biol. Med.* **84**, 227–245 (2015). <https://doi.org/10.1016/j.freeradbiomed.2015.03.022>.

[15] Miller, D.B., O'Callaghan, J.P. Biomarkers of Parkinson's disease: present and future. *Metabolism.* **64** (3 Suppl 1), S40–S46 (2015). <https://doi.org/10.1016/j.metabol.2014.10.030>.

[16] Rappaport, S.M., Barupal, D.K., Wishart, D., Vineis, P., Scalbert, A. The blood exposome and its role in discovering causes of disease. *Environ. Health. Perspect.* **122** (8), 769–774 (2014). <https://doi.org/10.1289/ehp.1308015>.

[17] Vrijheid, M., Slama, R., Robinson, O., Chatzi, L., Coen, M., van den Hazel, P., Thomsen, C., Wright, J., Athersuch, T. J., Avellana, N., Basagaña, X., Brochot, C., Bucchini, L., Bustamante, M., Carracedo, A., Casas, M., Estivill, X., Fairley, L., van Gent, D., Gonzalez, J. R., Granum, B., Grazuleviciene, R., Gutzkow, K. B., Julvez, J., Keun, H. C., Kogevinas, M., McEachan, R. R. C., Meltzer, H. M., Sabido, E., Schwarze, P. E., Siroux, V., Sunyer, J., Want, E. J., Zeman, F., Nieuwenhuijsen, M. J. The human early-life exposome (HELIX): project rationale and design. *Environ. Health. Perspect.* **122** (6), 535–544 (2014). <https://doi.org/10.1289/ehp.1307204>.

[18] Vrijens, K., Bollati, V., Nawrot, T.S. MicroRNAs as potential signatures of environmental exposure or effect: a systematic review. *Environ. Health. Perspect.* **123** (5), 399–411 (2015). <https://doi.org/10.1289/ehp.1408459>.

[19] Burbank, A.J., Sood, A.K., Kesic, M.J., Peden, D.B., Hernandez, M.L. Environmental determinants of allergy and asthma in early life. *J. Allergy. Clin. Immunol.* **140** (1), 1–12 (2017). <https://doi.org/10.1016/j.jaci.2017.05.010>.

[20] Jones, D.P., Park, Y., Ziegler, T.R. Nutritional metabolomics: progress in addressing complexity in diet and health. *Annu. Rev. Nutr.* **32**, 183–202 (2012). <https://doi.org/10.1146/annurev-nutr-072610-145159>.

[21] Vrijheid, M. The exposome: a new paradigm to study the impact of environment on health. *Thorax.* **69** (9), 876–878 (2014). <https://doi.org/10.1136/thoraxjnl-2013-204949>.

[22] Wishart, D., Arndt, D., Pon, A., Sajed, T., Guo, A. C., Djoumbou, Y., Knox, C., Wilson, M., Liang, Y., Grant, J., Liu, Y., Goldansaz, S. A., Rappaport, S. M. T3DB: the toxic exposome database. *Nucleic. Acids. Res.* **43** (Database issue), D928–934 (2015). <https://doi.org/10.1093/nar/gku1004>.

[23] Vineis, P., Chadeau-Hyam, M., Gmuender, H., Gulliver, J., Herceg, Z., Kleinjans, J., Kogevinas, M., Kyrtopoulos, S., Nieuwenhuijsen, M., Phillips, D. H., Probst-Hensch, N., Scalbert, A., Vermeulen, R., Wild, C. P., EXPOsOMICS Consortium. The exposome in practice: Design of the EXPOsOMICS project. *Int. J. Hyg. Environ. Health.* **220** (2 Pt A), 142–151 (2017). <https://doi.org/10.1016/j.ijheh.2016.08.001>.

[24] Ugai, T., Sasamoto, N., Lee, H. Y., Ando, M., Song, M., Tamimi, R. M., Kawachi, I., Campbell, P. T., Giovannucci, E. L., Weiderpass, E., Rebbeck, T. R., Ogino, S. Is early-onset cancer an emerging global epidemic? Current evidence and future implications. *Nat. Rev. Clin. Oncol.* **19** (10), 656-673 (2022). <https://doi.org/10.1038/s41571-022-00672-8>.

[25] Celebi Sozener, Z., Özbey Yücel, Ü., Altiner, S., Ozdel Oztürk, B., Cerci, P., Türk, M., Gorgülü Akin, B., Akdis, M., Yilmaz, I., Ozdemir, C., Mungan, D., Akdis, C. A. The External Exposome and Allergies: From the Perspective of the Epithelial Barrier Hypothesis. *Front. Allergy.* **3**, 887672 (2022). <https://doi.org/10.3389/falgy.2022.887672>.

[26] Kumar, M., Sarma, D. K., Shubham, S., Kumawat, M., Verma, V., Prakash, A., Tiwari, R. Environmental Endocrine-Disrupting Chemical Exposure: Role in Non-Communicable Diseases. *Front. Public. Health.* **8**, 553850 (2020). <https://doi.org/10.3389/fpubh.2020.553850>.

[27] Uppal, K., Walker, D. I., Liu, K., Li, S., Go, Y. M., Jones, D. P. Computational Metabolomics: A Framework for the Million Metabolome. *Chem. Res. Toxicol.* **29** (12), 1956-1975 (2016). <https://doi.org/10.1021/acs.chemrestox.6b00179>.

[28] Go, Y. M., Walker, D. I., Liang, Y., Uppal, K., Soltow, Q. A., Tran, V., Strobel, F., Quyyumi, A. A., Ziegler, T. R., Pennell, K. D., Miller, G. W., Jones, D. P. Reference Standardization for Mass Spectrometry and High-resolution Metabolomics Applications to Exposome Research. *Toxicol. Sci.* **148** (2), 531–543 (2015). <https://doi.org/10.1093/toxsci/kfv198>.

[29] Rappaport, S.M. Genetic Factors Are Not the Major Causes of Chronic Diseases. *PLoS. One.* **11** (4), e0154387 (2016). <https://doi.org/10.1371/journal.pone.0154387>.

[30] Wild, C.P., Scalbert, A., Herceg, Z. Measuring the exposome: a powerful basis for evaluating environmental exposures and cancer risk. *Environ. Mol. Mutagen.* **54** (7), 480–499 (2013). <https://doi.org/10.1002/em.21777>.

[31] Maitre, L., de Bont, J., Casas, M., Robinson, O., Aasvang, G. M., Agier, L., Andrušaitytė, S., Ballester, F., Basagaña, X., Borràs, E., Brochot, C., Bustamante, M., Carracedo, A., de Castro, M., Dedele, A., Donaire-Gonzalez, D., Estivill, X., Evandt, J., Fossati, S., Giorgis-Allemand, L., Gonzalez, J. R., Granum, B., Grazuleviciene, R., Gutzkow, K. B., Haug, L. S., Hernandez-Ferrer, C., Heude, B., Ibarluzea, J., Julvez, J., Karachaliou, M., Keun, H. C., Krog, N. H., Lau, C. H. E., Leventakou, V., Lyon-Caen, S., Manzano, C., Mason, D., McEachan R., Meltzer, H. M., Petraviciene, I., Quentin, J., Roumeliotaki, T., Sabido, E., Saulnier, P. J., Siskos, A. P., Siroux, V., Sunyer, J., Tamayo, I., Urquiza, J., Vafeiadi, M., van Gent, D., Vives-Usano, M., Waiblinger, D., Warembourg, C., Chatzi, L., Coen, M., van der Hazel, P., Nieuwenhuijsen, M. J., Slama, R., Thomsen, C., Wright, J., Vrijheid, M. Human Early Life Exposome (HELIX) study: a European population-based exposome cohort. *BMJ. Open.* **8** (9), e021311 (2018). <https://doi.org/10.1136/bmjopen-2017-021311>.

[32] Lochhead, P., Chan, A. T., Nishihara, R., Fuchs, C. S., Beck, A. H., Giovannucci, E., Ogino, S. Etiologic field effect: reappraisal of the field effect concept in cancer predisposition and progression. *Mod. Pathol.* **28** (1), 14-29 (2015). <https://doi.org/10.1038/modpathol.2014.81>.

[33] Janssen, B. G., Byun, H. M., Gyselaers, W., Lefebvre, W., Baccarelli, A. A., Nawrot, T. S. Placental mitochondrial methylation and exposure to airborne particulate matter in the early life environment: An ENVIRONAGE birth cohort study. *Epigenetics*. **10** (6), 536-544 (2015). <https://doi.org/10.1080/15592294.2015.1048412>.

[34] Dennis, K. K., Marder, E., Balshaw, D. M., Cui, Y., Lynes, M. A., Patti, G. J., Rappaport, S. M., Shaughnessy, D. T., Vrijheid, M., Barr, D. B. Biomonitoring in the era of the exposome. *Environ. Health. Perspect.* **125** (4), 502–510 (2017). <https://doi.org/10.1289/EHP474>.

[35] Jones, D.P. Redox theory of aging. *Redox. Biol.* **5**, 71-79 (2015). <https://doi.org/10.1016/j.redox.2015.03.004>.
